# Integrative analysis reveals pathways associated with sex reversal in *Cynoglossus semilaevis*

**DOI:** 10.7717/peerj.8801

**Published:** 2020-03-19

**Authors:** Zhan Ye, Weifeng Wang, Yaqun Zhang, Liping Wang, Yu Cui, Hengde Li

**Affiliations:** 1 National Demonstration Center for Experimental Fisheries Science Education, Shanghai Ocean University, Shanghai, China; 2 Key Laboratory of Aquatic Genomics, Ministry of Agriculture and rural affairs, Beijing Key Laboratory of Fishery Biotechnology, Chinese Academy of Fishery Sciences, Beijing, China; 3 College of Fisheries, Huazhong Agricultural University, Wuhan, China

**Keywords:** *Cynoglossus semilaevis (C. semilaevis)*, Transcriptome, Proteome, Integrative analysis, Sex reversal

## Abstract

Sex reversal is a complex biological phenomenon exhibited by* Cynoglossus semilaevis*. Some genetic females may irreversibly convert to pseudomales, thus increasing aquaculture costs because males grow much more slowly than females. In this study, an integrative analysis of transcriptome and proteome was performed to compare differences in gene and protein expression in females and pseudomales after gonad differentiation in *C. semilaevis*. Based on RNA-Seq results, 1893 genes showed differences in expression at the transcript level between females and pseudomales. Of these differentially expressed genes (DEGs), zona pellucida sperm-binding protein 4-like (LOC103393374 , ZP4), zona pellucida sperm-binding protein 4-like (LOC103396071, ZP4) and forkhead box L2 (*foxl2*) were highly expressed in females and doublesex and mab-3 related transcription factor 1(*dmrt1*) and doublesex and mab-3 related transcription factor 3 (*dmrt3*) were highly expressed in pseudomales. GO enrichment analysis results indicate that wnt signaling pathways and oocyte maturation are two terms enriched in female. At the protein level, Tandem Mass Tags analysis revealed that 324 proteins differed in their relative abundance between pseudomales and females. KEGG analysis found that pseudo-highly expressed proteins were enriched in the ubiquitin mediated proteolysis pathway. For integrative analysis, the Spearman correlation coefficient between the transcriptome and proteome was 0.59. Among 52 related genes, 46 DEGs (88%) were well matched in their levels of change in protein abundance. These findings reveal major active pathways in female and pseudomale gonads after sex reversal and provide new insights into molecular mechanisms associated with sex reversal regulatory network.

## Introduction

*Cynoglossus semilaevis* is a valuable commercial fish and is distributed along the coast of the Yellow Sea and the Bohai Sea of China (Jiang & Li, 2017). *C. semilaevis* is of ZW-type sex determination, with females being heterogametic (ZW♀) and males being homogametic (ZZ♂). Females have considerably higher growth rates than males, so that adult females are two to four times larger than adult males (Chen et al., 2009), and female fish are therefore more desirable in *C. semilaevis* aquaculture. However, during dph 50 to 100 some genetic females can develop into pseudomales, which grow just as slowly as normal males. Due to sex reversal, the cost of breeding *C. semilaevis* has increased and increasing and maintaining the level of females in a population has become an urgent and important issue in *C. semilaevis* aquaculture. In fact, sex reversal is so common that the percentage of females has gradually decreased to below 20% on average in recent years. Several reports have shown that sex reversal ratios are affected by temperature, but their conclusion are not consistent (Tian et al., 2011; Wang et al., 2014). Therefore, whether sex reversal ratios are affected by environmental factors remained uncertain. Our previous studies have elucidated the genetic architecture underlying sex reversal in *C. semilaevis*. We identified two loci associated with sex reversal of *C. semilaevis* by genome-wide association study (GWAS). These two loci are located on the Z chromosome in the third intron of the F-box and leucine-rich repeat protein 17 (*fbxl17*) gene (Cyn_Z_6676874) and the third intron of the *dmrt1* gene (Cyn_Z_8564889) (Jiang & Li, 2017; Cui et al., 2018). Genetic females that contain both the T allele of the Cyn_Z_6676874 locus and the A allele of the Cyn_Z_8564889 locus reverse into pseudomales and have provided scientific guidance for breeding and management of *C. semilaevis* aquaculture.

A set of sex-biased genes potentially associated with growth and reproduction in tongue sole were identified by brain transcriptome analysis (Wang et al., 2016) and transcriptomic analysis revealed candidate networks and genes for sexual dimorphism of body size for *C. semilaevis* (Wang et al., 2018). The process of sex reversal is accompanied with many changes in transcription and translation of certain genes. However, few studies have been published to identify sex-biased proteins involved in sex reversal in *C. semilaevis*. The integrative analysis of the transcriptome and proteome is an efficient tool for identifying differential expressed genes at the whole-genome level. Comparative transcriptomic and proteomic analysis was successfully used to study the development of the mouse stomach and its relationship with the development of gastric cancer at the whole-genome level (Li et al., 2018). However, the relationship between mRNA and protein levels did not follow a simple correlation in which higher mRNA levels always associate with higher protein levels (Becker et al., 2018). In addition, previous studies have demonstrated poor correlation between the transcriptome and the proteome (Anatole et al., 2011; Gygi et al., 1999; Kramer et al., 2014). This divergence might be due to post-transcriptional and translational processes regulating the location, quantity, and efficiency of proteins in the cell. In this study, the comparison of main active pathways in female and pseudomale gonads after sex reversal in *C. semilaevis* were explored through an integrative analysis of the transcriptome and proteome.

## Materials & Methods

### Ethics statement

All procedures involving the handling and treatment of fish used in this study were conducted with the approval of the animal care and use committee of Chinse Academy of Fishery Sciences. All animal procedures were carried out according to the guide for the care and use of laboratory animals and the animal welfare in China.

### Tissue materials and sex identification

In July 2018, 100 *C. semilaevis* of 120 days post-hatch (dph) were randomly selected from Tianjin Haisheng Aquaculture Company and their fins and gonads sampled. In our study, experimental fish are taken from the same pond, the temperature of which was 22 °C–23 °C, which will exclude all environmental impacts. Fins were stored in 100% ethanol and the DNA was extracted using the TIANamp marine animal DNA kit following the manufacturer’s protocol (TIANGEN^^®^^ Biotech Co., Ltd., Beijing, China). Gonads were frozen in liquid nitrogen then stored at −80 °C and RNA extracted using the TIANamp marine animal RNA kit following the manufacturer’s protocol (TIANGEN^^®^^ Biotech Co., Ltd., Beijing, China). The genetic sex was then determined with fin DNA using a previously described sex-specific marker (Liu et al., 2014). Phenotypic sex was determined by sequencing the Cyn_Z_6676874 and Cyn_Z_8564889 loci and verified by tissue sectioning of gonads (Figs. S1 and S2). The details of tissue sectioning are described in our previous study (Jiang & Li, 2017). Finally, gonad tissues from 4 pseudomales and 4 females were selected for transcriptomic and proteomic analysis.

### RNA-Seq analysis

Gonad samples of 4 pseudomales and 4 females were subjected to RNA-Seq using an Illumina HiSeq Xten at the Novogene Biotechnology Co. Ltd (Beijing, China). For RNA-Seq, 3 µg total RNA from each sample was used to enrich mRNA for cDNA library construction. The cDNA library was generated using the NEB Next^®^ UltraTM RNA Prep Kit for Illumina^®^ (NEB, USA) following the manufacturer’s protocol. Raw reads were filtered by removing low-quality reads with ambiguous nucleotides and adapter sequences of “AGATCGGAAGAGC” using FastQC software. Clean data were then aligned using Hisat2 (https://ccb.jhu.edu/software/hisat2/index.shtml) and mapped to *C. semilaevis* reference genome (NCBI: GCF_000523025.1_Cse_v1.0_genomic). Sequences were assembled based on alignment results and used for further analysis. Gene expression levels were further normalized using the fragments per kilobase of transcript per million mapped reads (FPKM) method to eliminate the influence of differences in gene lengths and the amount of sequencing data on calculations of gene expression (Wang et al., 2018). Differentially expressed genes (DEGs) between females and pseudomales were identified with an absolute log2FC ≥ 2 and a false discovery rate-adjusted P (*q* value) <0.05 using the ballgown package (https://www.rdocumentation.org/packages/ballgown). The raw data of transcriptomes and proteomics obtained in this bioproject (PRJNA586726), have been deposited in Genbank-SRA under the accession numbers (SRR10492824 –SRR10492831).

### qRT-PCR verification

To validate the results of high-throughput transcriptome sequencing, 16 DEGs were selected for real-time quantitative reverse transcription PCR (qRT-PCR). All genes used for quantification were selected based on factors such as PCR efficiency, gene expression level, and expression difference level. *C. semilaevis* β*-actin* gene was used as an internal reference (Li et al., 2010). Primers for the 16 DEGs were designed using Primer-blast based on the gene sequences from the RNA-Seq datasets (Table 1). Total RNA (1 µg) was reverse transcribed using the TOYOBO Reverse Transcription Kit then qRT-PCR conducted using SYBR^®^ Green Reagents with 15 µL reactions, each containing 9 µL SYBR^®^ Green, 0.3 µL forward primer, 0.3 µL reverse primer, 0.6 µL cDNA, and 4.8 µL ddH_2_O. The following PCR amplification procedure was used: 95 °C for 2 min, followed by 40 cycles of 95 °C for 15 s, 60 °C for 30 s, and 72 °C for 30 s. All reactions were repeated 3 times (Livak & Schmittgen, 2001) and disassociation curve analysis performed using an ABI 7500 (Applied Biosystems, Foster City, CA, USA) fast real-time PCR system. Relative expression levels for genes of interest in females and pseudomales were then calculated with the 2^−^^△△^^Ct^ (Livak & Schmittgen, 2001) method and the log2 fold change obtained from qRT-PCR and RNA-Seq was compared.

**Table 1:** Primers used for quantitative qRT-PCR.

**Gene**	**Primers**	**Sequence (5′∼3′)**
β-actin	For	GCTGTGCTGTCCCTGTA
	Rev	GAGTAGCCACGCTCTGTC
LOC103393374	For	GTAAGGTGGCGTACGTCGAA
	Rev	GAGAAGCAACCACACCGAGT
LOC103396071	For	GTTGTGCCTGTGACTGTGTCC
	Rev	CCGTTTGCATTTGGGGTTCC
gtf3a	For	GCTGCGAAGCTGAGAAAAAC
	Rev	AAACAAAACCAAATCCGTGTTCA
LOC103391323	For	CCGCCCTGTCCTTTCCAAT
	Rev	AGAGCACATGCTCACATCTCA
sbspon	For	GAGTTGACCTGTTTGCTCCTGG
	Rev	CAGTAGCACGTCCCATAAACGC
cystm1	For	TCTGGAACGTGCTCTTTCGT
	Rev	CCCCGTTCTTCCTGTGGTTA
LOC103392682	For	CCGACGTCAAAGGAGGGAA
	Rev	TGAAGACGTGTGTGTGTCCATA
LOC103394689	For	CAGGCCAAGCACAAAGACTG
	Rev	CCTCTCTTTCTTGAGCGCGA
LOC103391404	For	AGAAAGGAGTGTTGACCACAGTTG
	Rev	TACCAGCACAACCTTTGGACTGT
poc1a	For	CTTTCCGGAGTTTTGGATGGAA
	Rev	CCGTGCTTTAGCGATCCA
LOC103392072	For	ACAGAGGCCCGTAATGCCA
	Rev	TTTCTCAAGGACCTCAGGCTTC
LOC103379620	For	GAAGAGCCATGTTCAGCCAGA
	Rev	GGGTCAGTTCTACTCAAAGCCT
dmrt1	For	CCACAACATGCCCTCTCAGTA
	Rev	CCAGGTTGCAGATGGAAGGAA
dmrt3	For	CAACCTGAGGAGAGCCGATA
	Rev	GGAGCTGACGGGATACTTCT
foxl2	For	GAAGTCACCAAGTCTCCGGG
	Rev	AGCACTGGCTAAGTACAGCG
wnt9b	For	CGCAGTACGGACCTGGTCTT
	Rev	CATGGCCGTGTTGTAACCCC

### Protein extraction and labeling

Frozen samples of pseudomale and female gonad tissues were ground in liquid nitrogen then the 100 mg of powdered sample transferred into a 1.5 mL tube containing 600 µL phenol extract and protease inhibitor. Samples were then ultrasonicated on ice then mixed with a phenol-tris-HCL saturated solution of equal volume for 30 min at 4 °C. After centrifugation at 15,000 × g for 15 min at 4 °C, the upper phenol layer was transferred to a new tube and proteins precipitated using 0.1 M ammonium acetate-methanol at −20 ° C. Proteins were quantified using the BCA protein concentration assay (Smith et al., 1985). Next, 100 µg of protein was reduced with 120 µL of reducing agent buffer (10 mM DTT, 8 M urea, 100 mM TEAB, pH 8.0) at 60 °C for 1 h. Reduced proteins were then alkylated for 40 min at room temperature in the dark by the addition of IAA to a final concentration of 50 mM. Proteins were then digested with 2 µL of 1 µg/µL trypsin per sample for 12 h at 37 °C (Wisniewski et al., 2009). Digested peptides were collected, mixed with 50 µL of 200 mM TEAB buffer, then centrifuged at 12,000 rpm for 20 min and the resulting lower layer collected and lyophilized. Samples were then resuspended in 200 mM triethylammonium bicarbonate (TEAB) and the peptides labeled using the TMT labeling reagent for 1.5 h at room temperature (Xiao et al., 2016; Guerreiro et al., 2014).

### Proteomic analysis

After labeling, samples were mixed in a tube and dried with a vacuum pump. Pooled mixtures were fractionated using an Agilent 1100 HPLC with an ultraviolet (UV) absorption detector (210 nm and 280 nm) and a flow rate of 300 µL/min. Eluted peptides were grouped into 10 final fractions, desalted on an Agilent Zorbax Extend C18 column (2. 1 ×150 mm), and vacuum dried. LC–MS/MS analysis of each fraction was then performed on an EASY-nLC 1200 liquid chromatograph and peptides eluted using a gradient of 2–80% acetonitrile with 0.1% formic acid for 120 min on a Q-Exactive mass spectrometer (Thermo, USA). The MS scanning range was 300–1,600 m/z and the top 10 most intense signals were selected for further MS/MS analysis. The resolutions of the MS and the MS/MS scans were 70,000 and 17,500, respectively, and the dynamic exclusion set to 15 s. Experimental data was analyzed by Proteome Discoverer ™ 2.2 (Thermo, USA) software. The protein database used was Acanthomorphata uniprot and the false discovery rate of peptide identification was below 1%. After mass spectrometry analysis, results were retrieved from the database and relevant proteins separated. Differentially expressed proteins (DEPs) were identified based on fold changes (FC) calculated by comparing pseudomale and female groups and *p*-values were obtained by t-tests. Proteins that were significantly differentially expressed were defined by FC >1.5 or FC <2/3 and a *p*-value <0.05.

### GO and KEGG analysis

Gene Ontology (GO) functional enrichment analysis was used to determine biological functions significantly associated with DEGs or DEPs, including relevant molecular functions, cell components, and biological processes. Kyoto Encyclopedia of Genes and Genomes (KEGG) pathway enrichment analysis was used to aid biological interpretation of DEGs and DEPs. GO functional enrichment analysis for DEGs and DEPs was conducted using the online tool DAVID (https://david.ncifcrf.gov/) and KEGG pathway enrichment analysis performed with the KOBAS 3.0 online tool (http://kobas.cbi.pku.edu.cn/). Since *C. semilaevis* is a non-model species, genes were mapped to the zebrafish function database for analysis.

### Correlations between the transcriptome and proteome

The Spearman correlation coefficient between mRNA and protein expression was calculated for genes differentially expressed in both transcriptome and proteome analysis to avoid masking effects of random variation. DEG sequences were extracted from RNA-Seq data and used as a nucleic acid database for alignment. Amino acid sequences of peptides from DEPs were aligned as queries to the DEGs database using the tblastn (https://blast.ncbi.nlm.nih.gov/Blast.cgi) command, then the genes differentially expressed at both the transcriptome and proteome levels extracted. An alignment result of identity ≥ 80%, e-value ≤ 1, and gap = 0 indicated a perfect matched between mRNA and protein sequences. Spearman correlation coefficients were calculated based on mean fold changes for mRNA and protein expression.

## Results

### Characteristics of high-throughput sequencing data

A total of 194 Gb of sequence was generated using eight *C. semilaevis* gonadal tissue libraries. After quality control and adapter removal, 150.7 Gb of clean reads were obtained, 85.14%–89.43% of which were mapped to *C. semilaevis* genome. The assembled transcriptome consists of 46,317 transcripts belonging to 25,761 genes. A total of 2,172 (FDR < 1%) reliable proteins were identified by combining high-throughput TMT technology with high-resolution LC-MS/MS technology (Table 2).

**Table 2:** The statistics of transcriptome and proteomic data.

**Type of data**	**Number**
Raw data	194.0 (Gb)
Clean data	150.7 (Gb)
Transcripts	46,317
Genes	25,761
Mapped rate	85.14%∼89.43%
Reliable protein	2172 (FDR <1%)

### Differentially expressed genes between females and pseudomales

**Figure 1:**
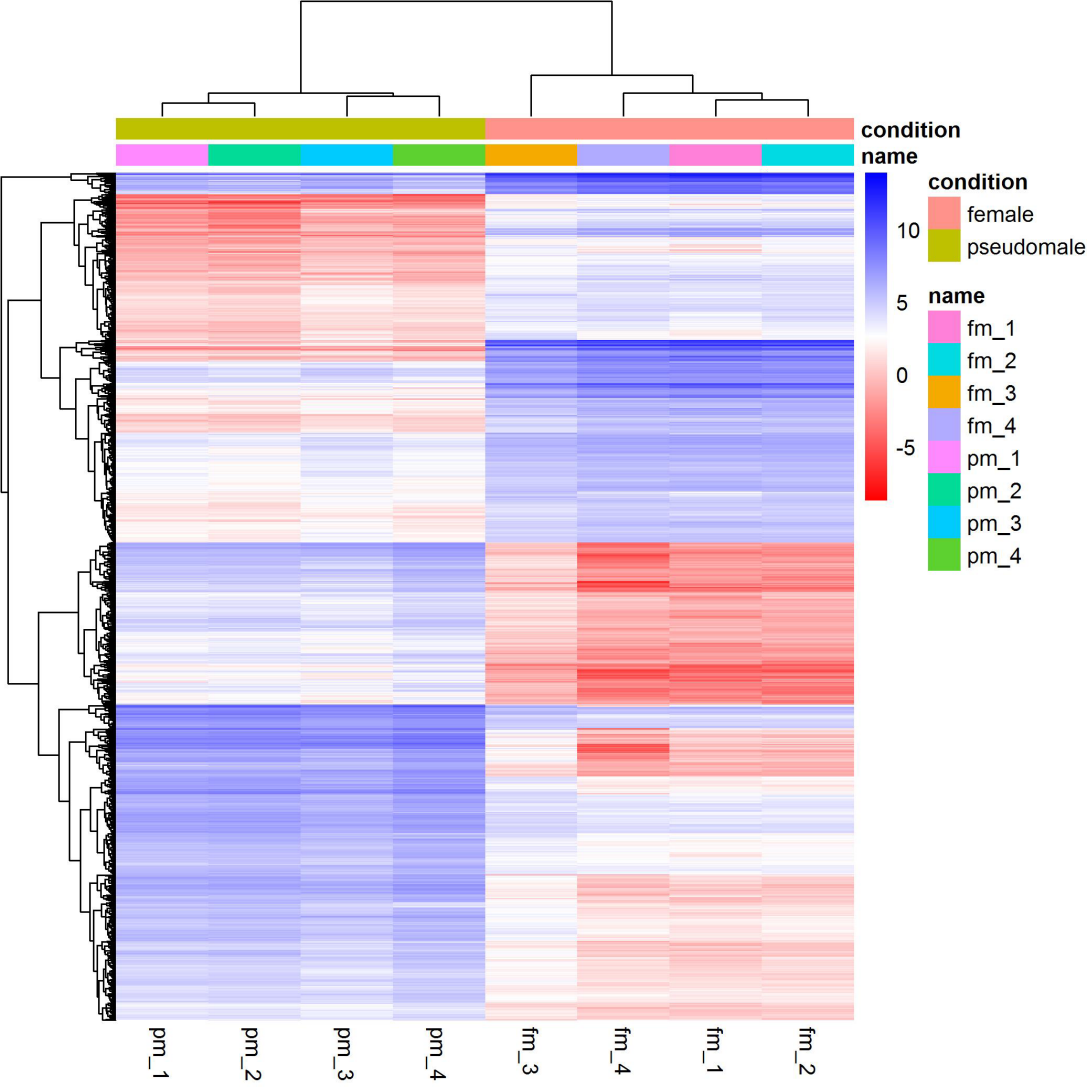
Cluster analysis of RNA-Seq datasets. The cluster analysis was performed with all genes, and FPKM was taken as the expression level of all genes. “name” is sample ID, fm: female, pm: pseudomale.

We identified a total of 1,893 differentially expressed genes between females and pseudomales (Table S1). Of all the differentially expressed genes (DEGs), 785 genes were more highly expressed in females while 1,108 genes were more highly expressed in pseudomales (Fig. 1). These differentially expressed genes included several sex-related genes, such as *dmrt1*, *dmrt3* and *foxl2*. The expression levels of *dmrt1* and *dmrt3* in pseudomales were 5.09 FC and 16.82 FC higher than in females, respectively. The expression level of *foxl2* in females was 44.21 FC higher than in pseudomales.

**Figure 2:**
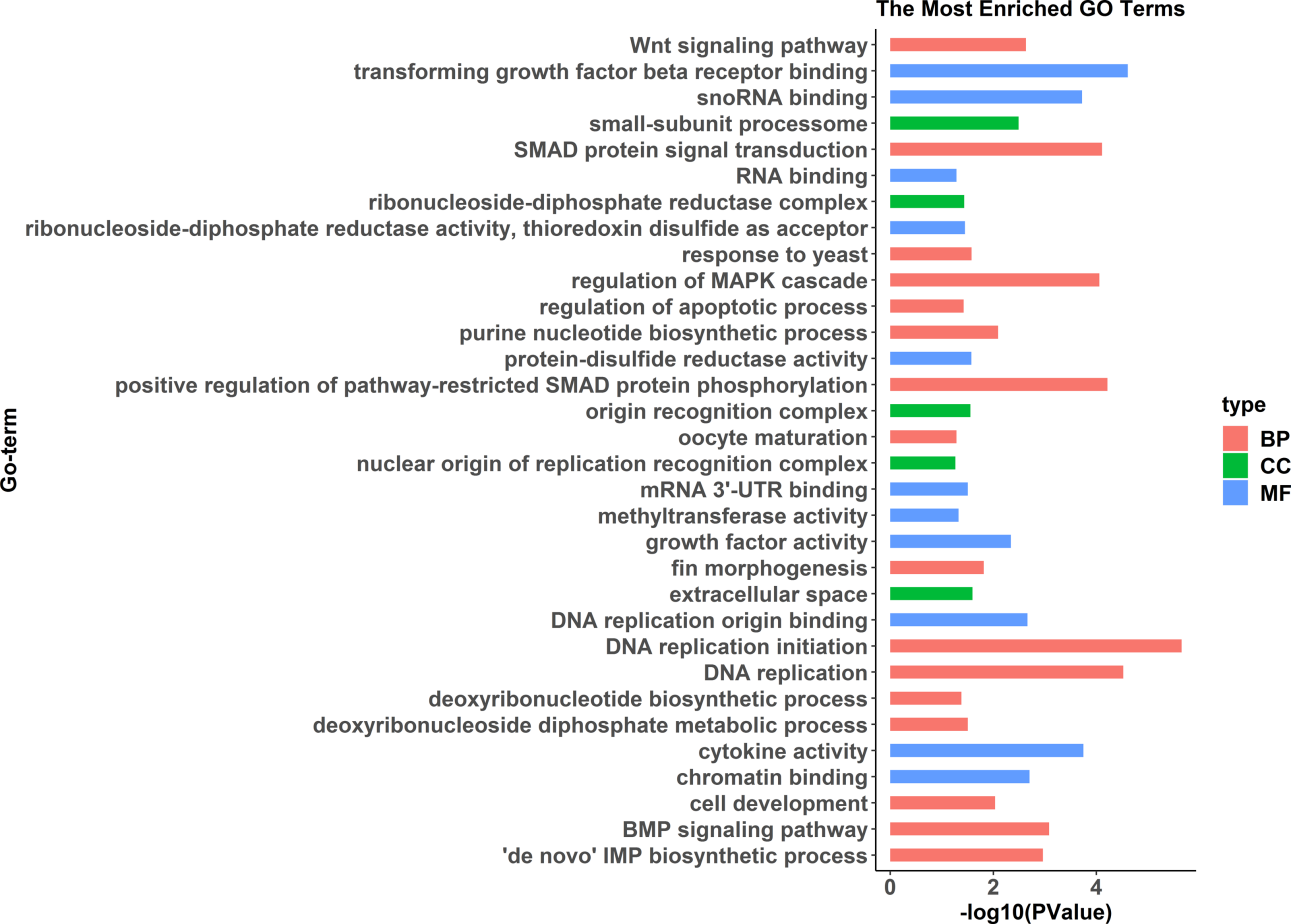
All Gene Ontology (GO) enrichment terms of all female highly expressed genes. The DEGs were categorized based on GO annotation, and the number of each category was displayed based for biological processes (BP), molecular functions (MF), and cellular components (CC).

**Figure 3:**
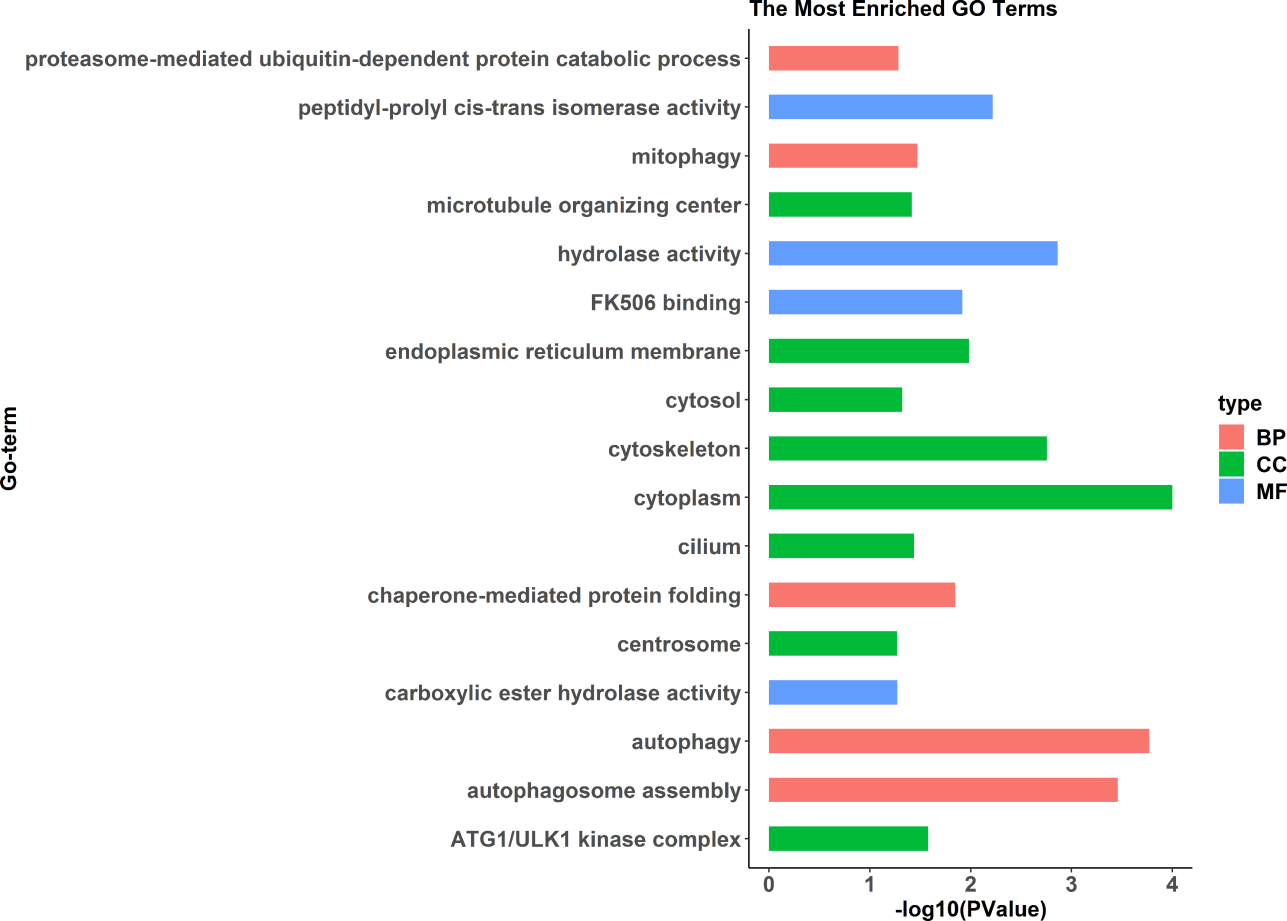
All Gene Ontology (GO) enrichment terms of all pseudomale highly expressed genes. The DEGs were categorized based on GO annotation, and the number of each category was displayed based for biological processes (BP), molecular functions (MF), and cellular components (CC).

GO enrichment analysis indicated significant enrichment of 49 GO terms (*p* < 0.05). Among them, 32 GO terms were identified from 785 genes highly expressed in females (Fig. 2) and 17 GO terms were identified from 1,108 genes highly expressed in pseudomales (Fig. 3). Of these 32 GO terms highly expressed in females, DNA replication initiation is most significant. In addition, biological processes including wnt signaling pathways, cell development, and oocyte maturation are enriched. Cellular component category extracellular space is enriched with the largest number of differential genes. For molecular functions classification, transforming growth factor beta receptor binding was most significantly enriched. Moreover, the top three categories were growth factor activity, methyltransferase activity, and cytokine activity. Among the 17 GO terms highly expressed in pseudomales, autophagy enrichment was the most significant and cytoplasm enriched with the largest number of differential genes.

**Figure 4:**
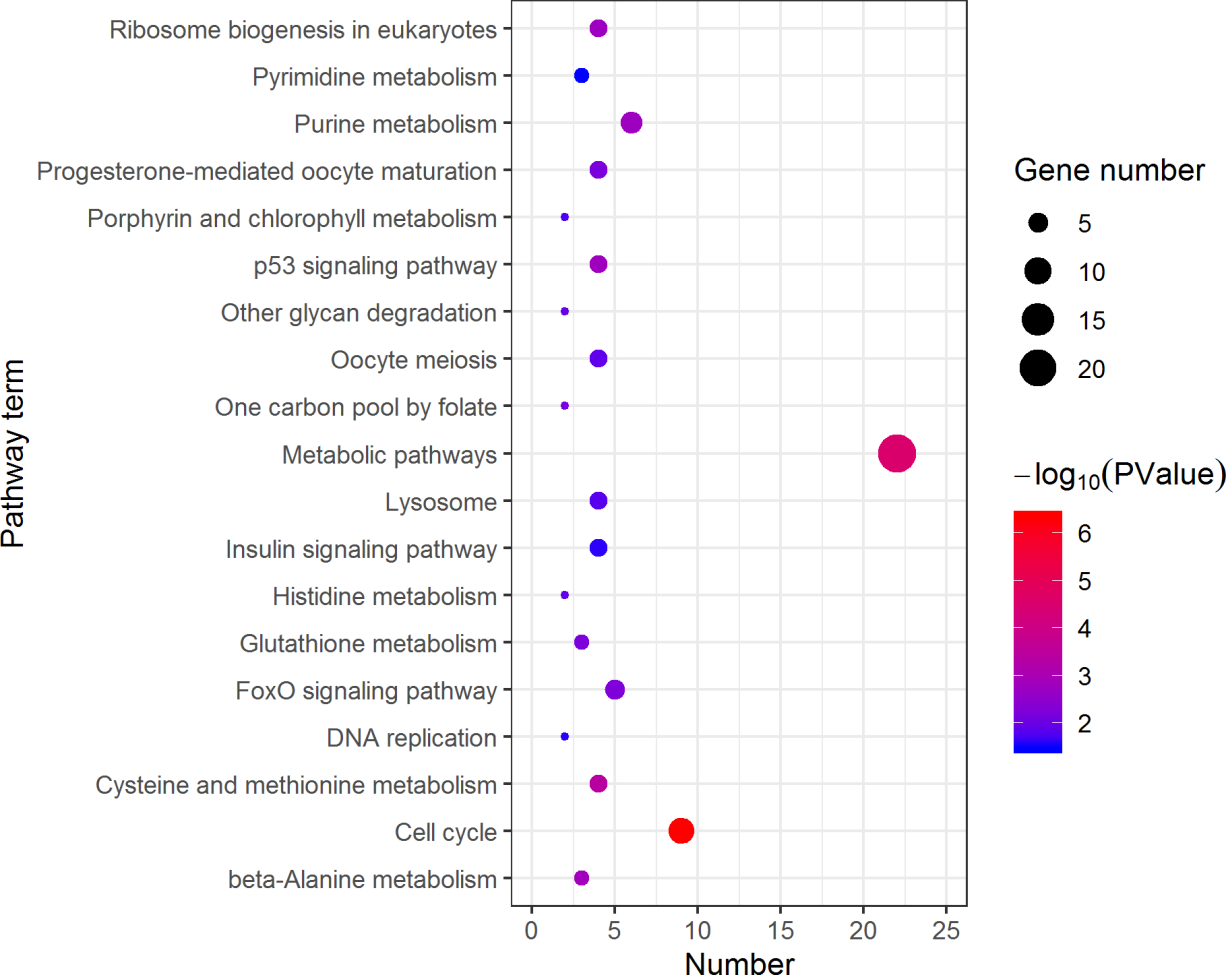
Kyoto Encyclopedia of Genes and Genomes (KEGG) pathway classification of the 785 female highly expressed genes. The larger the size of the circle, the more genes were enriched, −log10 (*P* Value) represents the significance of enrichment, red means high significance, blue means low significance.

**Figure 5:**
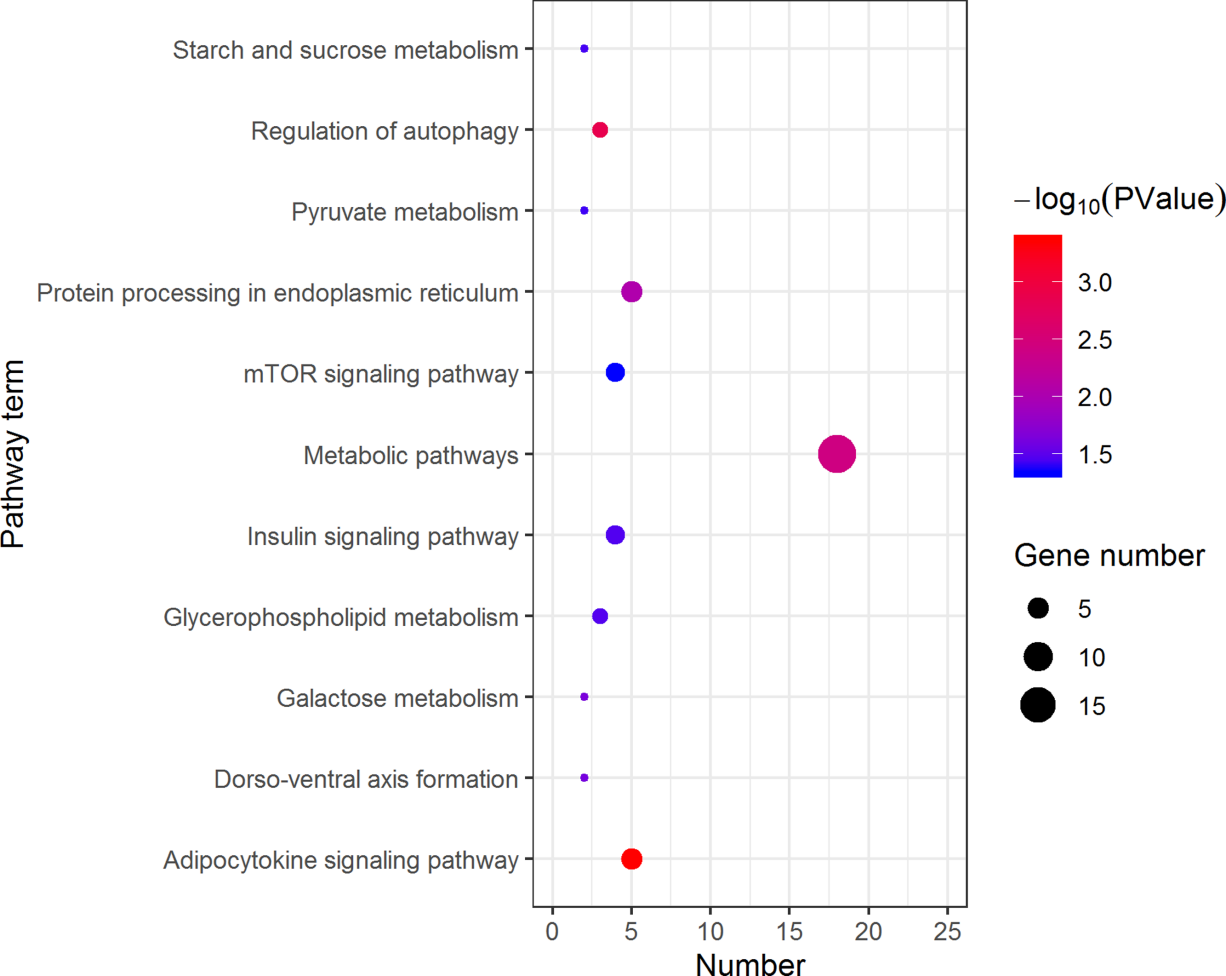
Kyoto Encyclopedia of Genes and Genomes (KEGG) pathway classification of the 1,108 pseudomale highly expressed genes. The larger the size of the circle, the more genes were enriched, −log10 (*P* Value) represents the significance of enrichment, red means high significance, blue means low significance.

KEGG analysis assigned the 1893 DEGs to 30 pathways, in which 19 pathways of highly expressed in females were enriched (Fig. 4), and 11 pathways that are highly expressed in pseudomales are enriched (Fig. 5). Of the 19 pathways, cell cycle was the most significantly enriched. In addition, progesterone-mediated oocyte maturation and oocyte meiosis closely related to female germ cells were enriched. Of the 11 pathways, adipocytokine signaling pathway is the most significant enriched.

### The validation of RNA-Seq

**Figure 6:**
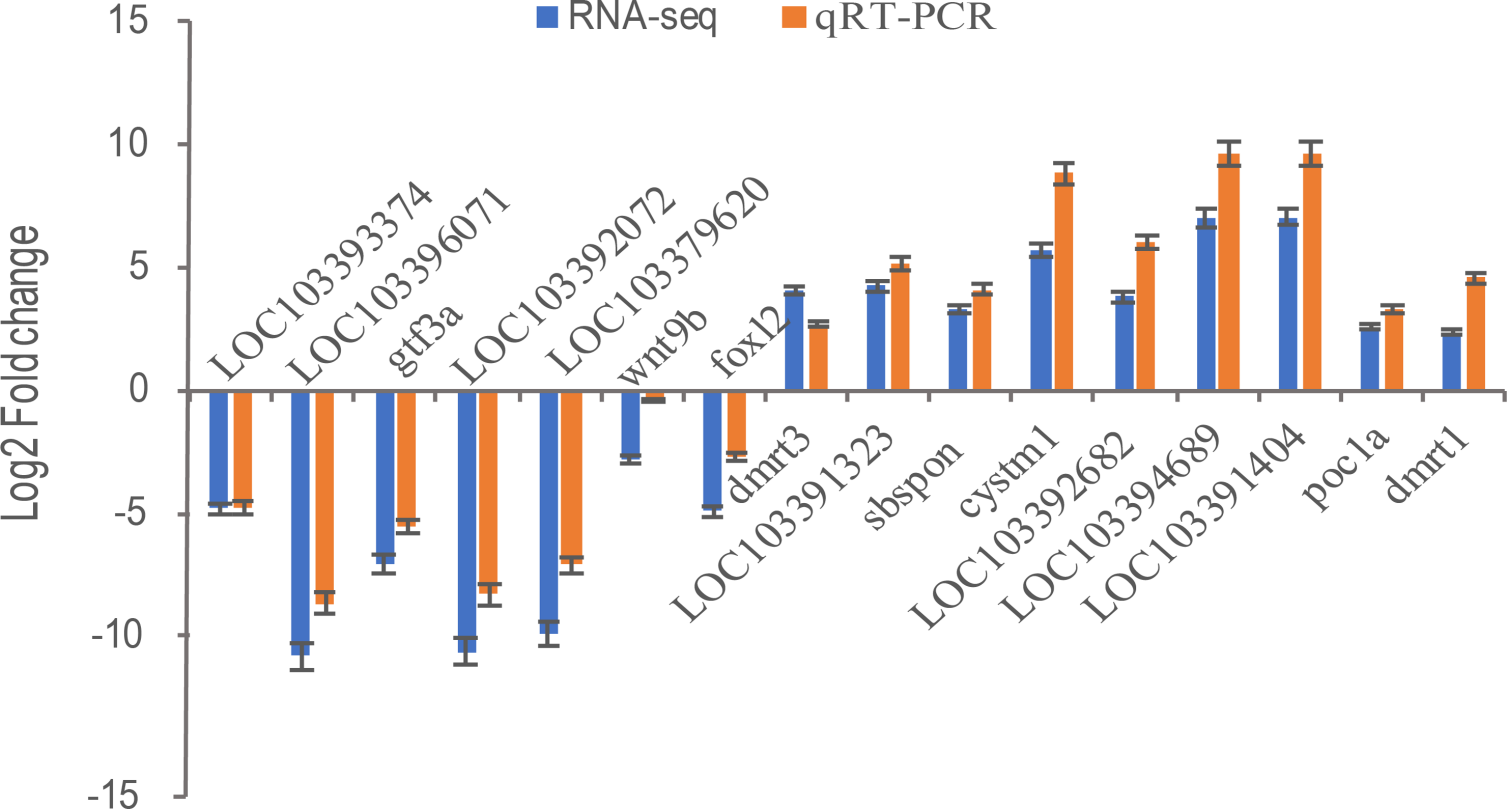
The relative expression levels of 16 selected DEGs by qRT-PCR and RNA-Seq. Sixteen DEGs including nine up-regulated genes were highly expressed in pseudomale (*dmrt1*,* dmrt3*, *sbspon*, *cystm1*, *poc1a*, LOC103391323, LOC103392682, LOC103394689, LOC103391404) and seven down-regulated genes (*foxl2*, *wnt9b*, *gtf3a*, LOC103393374, LOC103396071, LOC103392072, LOC103379620) were selected for qRT-PCR. β-Actin was used for the internal reference. The expression fold changes of 16 genes in pseuomale versus female gonads detected by qRT-PCR and RNA-Seq were calculated by 2^−ΔΔCt^ and FPKM, respectively. And, these genes’ log2 fold change values of qRT-PCR and RNA-Seq are shown in red and blue, respectively.

Transcriptional regulation revealed by RNA-Seq data was confirmed in a biologically independent experiment using qRT-PCR. A total of 16 genes, including general transcription factor IIIA (*gtf3a*), somatomedin B and thrombospondin type 1 domain containing (*sbspon*), cysteine rich transmembrane module containing 1 (*cystm1*)*,* POC1 centriolar protein A (*poc1a*), doublesex and mab-3 related transcription factor 1 (*dmrt1*), doublesex and mab-3 related transcription factor 3 (*dmrt3*), wingless-type MMTV integration site family, member 9B (*wnt9b*), forkhead box L2 (*foxl2*), zona pellucida sperm-binding protein 4-like (LOC103393374, ZP4), zona pellucida sperm-binding protein 4-like (LOC103396071, ZP4), charged multivesicular body protein 1b-like (LOC103391323), LOC103392682, cilia- and flagella-associated protein 157-like (LOC103394689), tubulin alpha-1C chain-like (LOC103391404), NAD(P)H dehydrogenase [quinone] 1-like (LOC103392072), and LOC103379620 were selected for qRT-PCR analysis. 16 DEGs including nine up-regulated genes were highly expressed in pseudomale (*dmrt1*, *dmrt3*, *sbspon*, *cystm1*, *poc1a*, LOC103391323, LOC103392682, LOC103394689, LOC103391404) and others were down-regulated in pseudomale. Except for the gene of *dmrt3*, other genes were more quantitative than RNA-Seq, but similar up-regulation or down-regulation patterns of these genes were observed in qRT-PCR and RNA-Seq results (Fig. 6). Further Pearson correlation analysis of qRT-PCR and RNA-Seq showed that their correlation was *ρ* = 0.98. This indicates that qRT-PCR can effectively validate the results of RNA-Seq.

### Proteomic analysis

**Figure 7:**
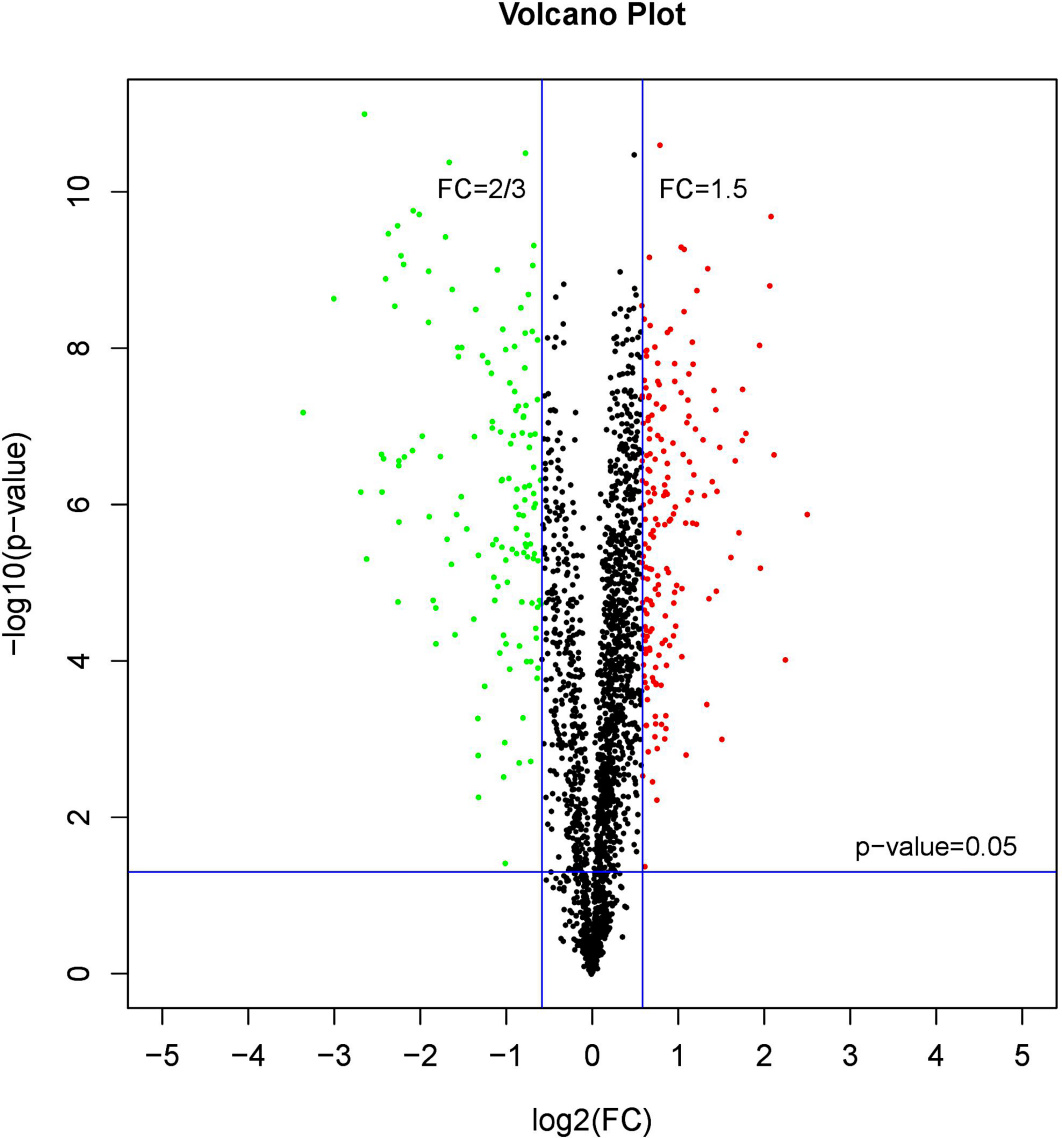
A volcano plots of DEPs under female compare with pseudomale. The red indicated up-regulated proteins (*p*-value < 0.05 and FC > 1.5), green indicated down-regulated proteins (*p*-value < 0.05 and FC < 2/3), black indicated no significant change, the further the log2(FC) and −log10 (*p*-value) are from 0, the greater the difference in protein expression.

**Figure 8:**
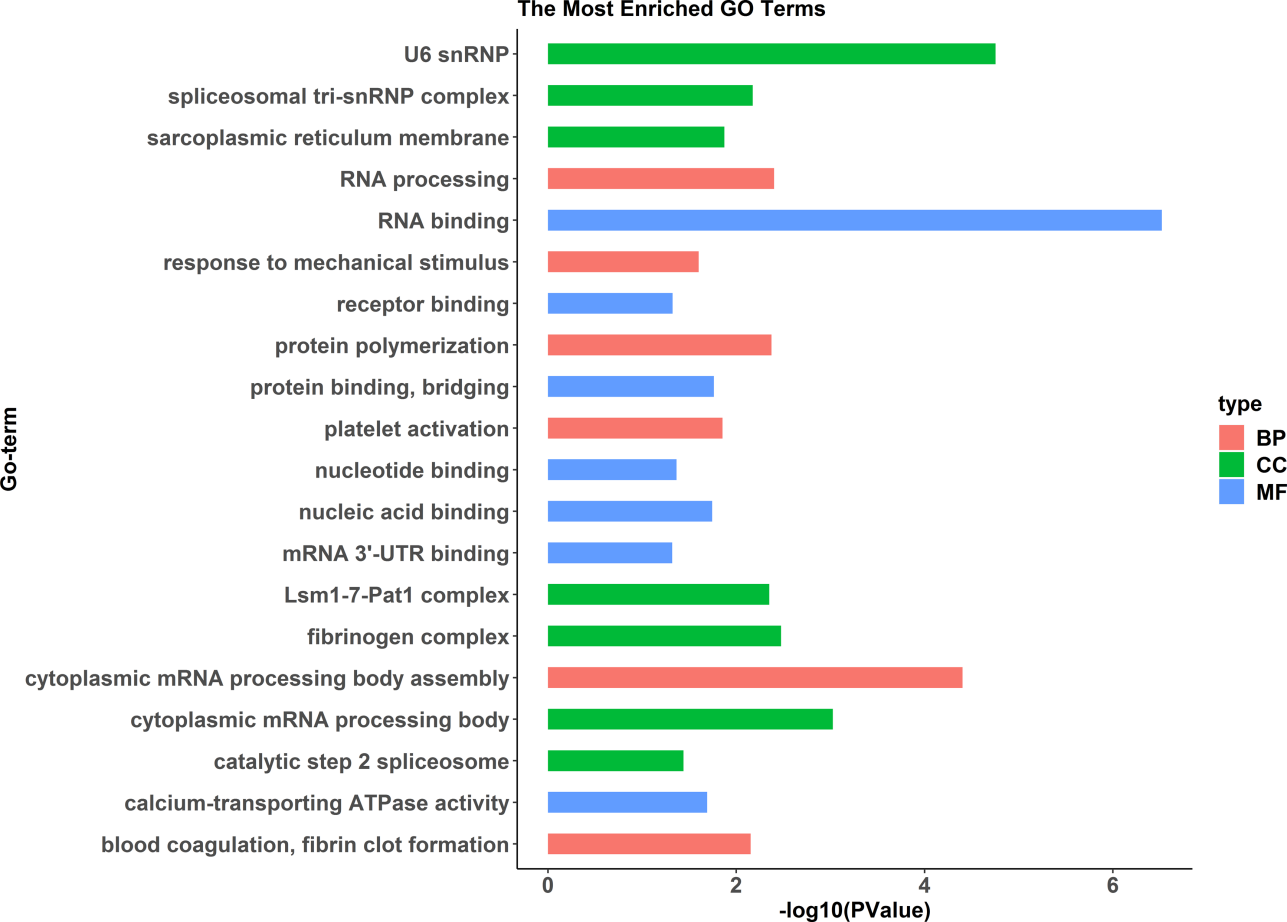
GO functional annotation histogram of the female highly expressed proteins. The *Y* axes represents the annotation of the GO term, and the *X* axes is −log10 (*P* Value), representing the significance of enrichment. BP, Biological Process; CC, Cellular Component; MF, Molecular Function.

**Figure 9:**
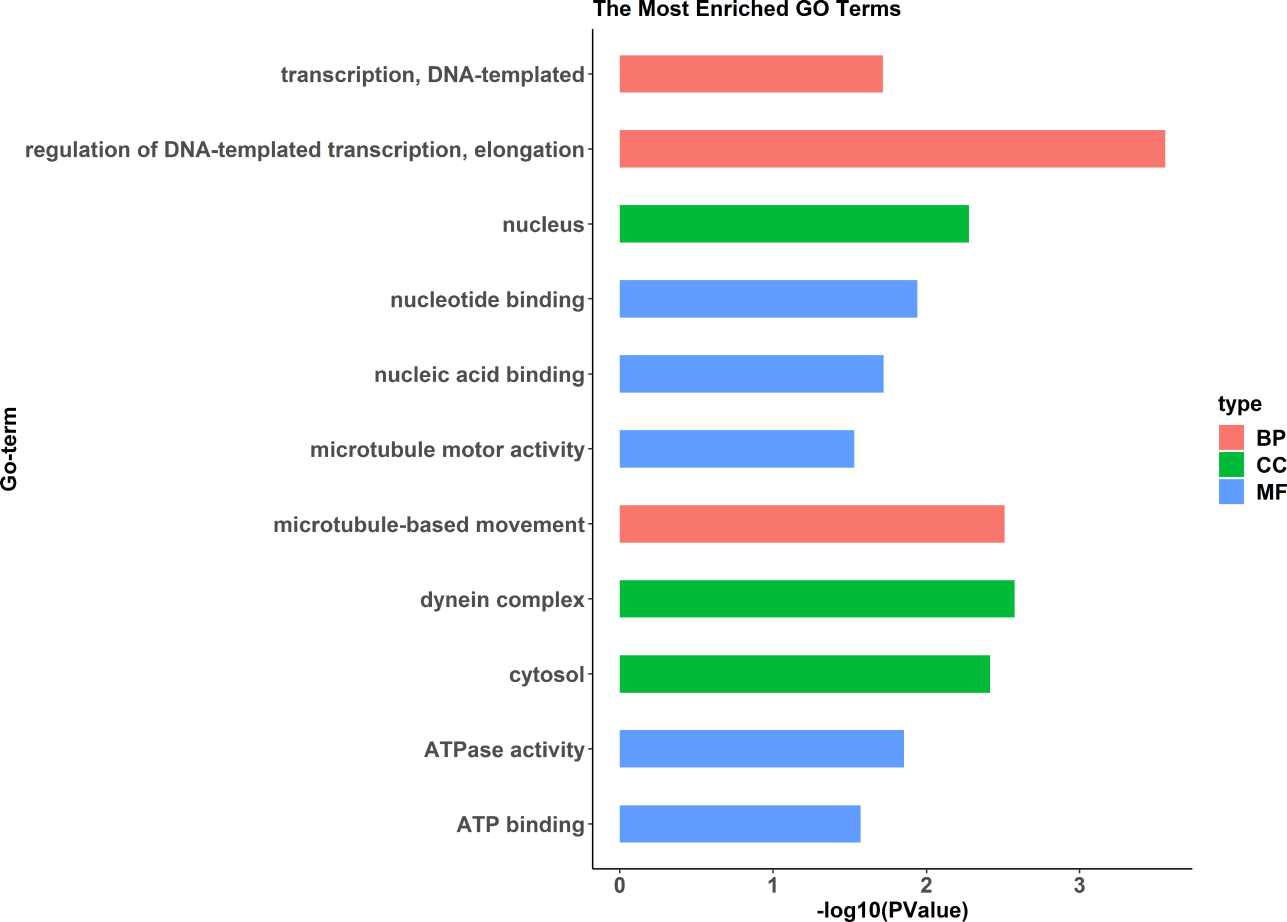
GO functional annotation histogram of the pseudomale highly expressed proteins. The *Y* axes represents the annotation of the GO term, and the *X* axes is −log10 (*P* Value), representing the significance of enrichment. BP, Biological Process; CC, Cellular Component; MF, Molecular Function.

A comparative proteome survey was performed of females and pseudomales using the TMT technique to complement the transcriptome experiments. Out of 2172 reliable proteins, 184 up-regulated proteins and 140 down-regulated proteins were identified in pseudomales compared to females (Fig. 7 and Table S2). Enrichment analysis of DEP’s biological process showed that cytoplasmic mRNA processing body assembly, RNA processing and protein polymerization are the top three females with high expression of DEP enrichment (Fig. 8), while regulation of DNA-templated transcription, elongation is the high expression of DEPs in pseudomales enrich the most significant term (Fig. 9). In addition, U6 snRNP and nucleus are two terms in which female and pseudo-male highly expressed DEPs are enriched in cell component categories, respectively. RNA binding and nucleotide binding are the two main categories of high expression DEPs in females and pseudomales under molecular function, respectively.

**Figure 10:**
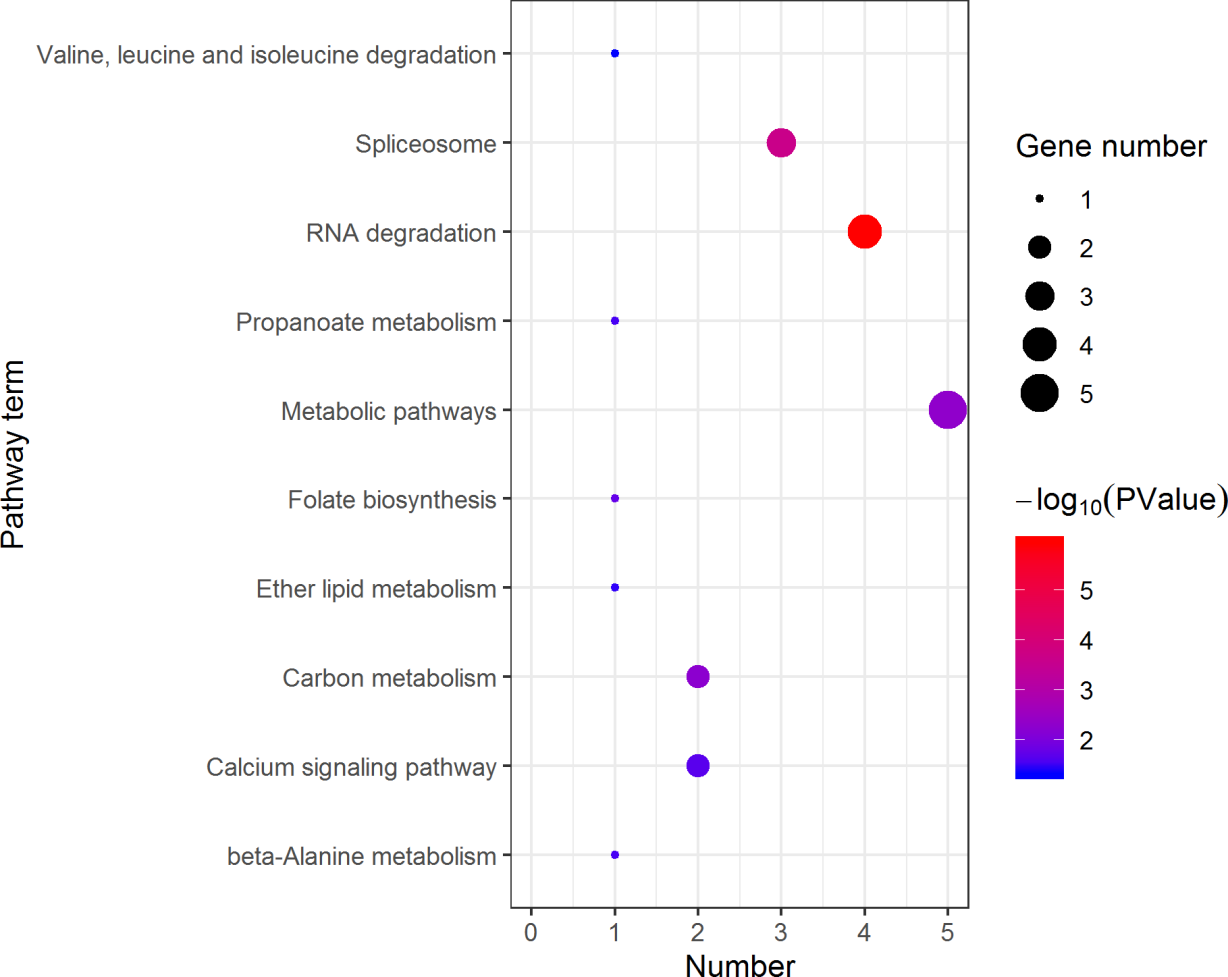
Distribution of KEGG pathway mapped by 140 female overexpressing proteins. The larger the size of the circle, the more genes were enriched, −log10 (*P* Value) represents the significance of enrichment, red means high significance, blue means low significance.

**Figure 11:**
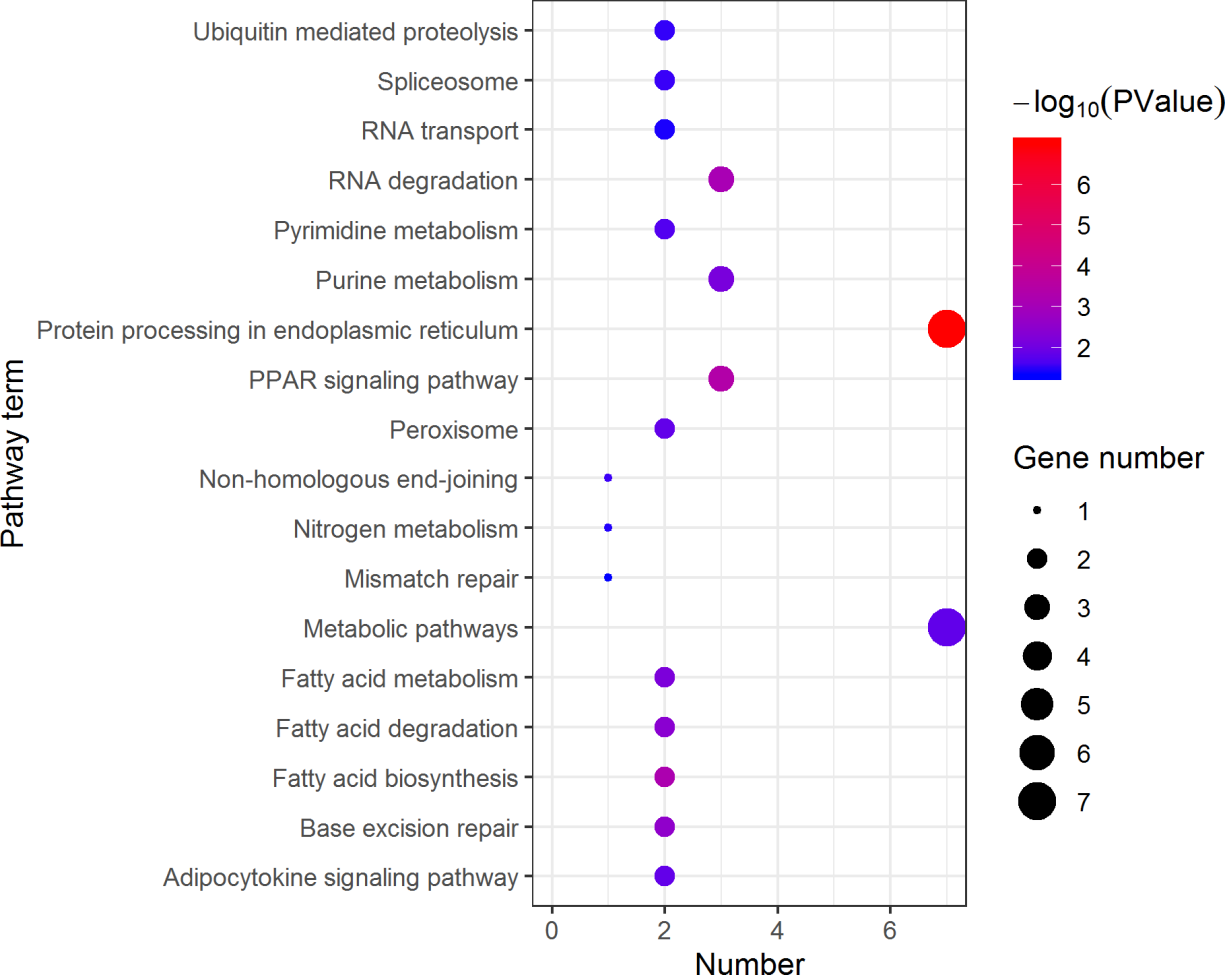
Distribution of KEGG pathway mapped by 184 pseudomale overexpressing proteins. The larger the size of the circle, the more genes were enriched, −log10 (*P* Value) represents the significance of enrichment, red means high significance, blue means low significance.

KEGG pathway analysis of the female highly expressed proteins revealed that RNA degradation was the most significantly pathway, and the following is spliceosome and metabolic pathways (Fig. 10). However, KEGG pathway analysis of the pseudomale highly expressed proteins founded that protein processing in endoplasmic reticulum, PPAR signaling pathway and fatty acid biosynthesis were the top three pathways (Fig. 11).

**Table 3:** Genes co-expressed at the transcriptome and proteome levels.

**Regulation**	**Gene**	**Protein**
	arl1	A0A146NN45
	cdh15	A0A096MDF5
	dusp23	A0A146ZXK5
	gabarap	H2TXA4
	ipo4	E6ZID3
	LOC103377360	A0A087XAG8
	LOC103377360	H2TXA1
	LOC103377360	P83299
	LOC103378288	A0A0C5I1M5
	LOC103382184	A0A087XC31
Up Genes	LOC103382184	Q4SP49
	LOC103383427	E6ZII0
Up Proteins	LOC103385166	G3P7M3
	LOC103386869	D9I8D0
	LOC103386869	H2U6U7
	LOC103394224	A0A146MKB7
	LOC103394643	A0A087XC31
	LOC103394643	Q4SP49
	LOC103394879	A0A146MPK8
	LOC103396142	A0A1A8D0E5
	LOC103397615	A0A146MPK8
	LOC103397682	A0A087XS77
	LOC103397682	A0A0F8CHP8
	map1lc3a	A0A1A8ANY3
	mindy3	A0A146X619
	prkar2a	H3D398
	rcn2	A0A1A7WGQ0
	rsph1	H2SC39
	trim33	G3NML3
	ublcp1	Q4RRW7
	ufd1	A0A146QYH4
	xrn2	G3PDN6
	yod1	A0A0F8AP91
	sh3glb2	A0A087XGK3
	arhgdia	A0A0F8AR61
	LOC103392079	A0A146NUM7
	LOC103395384	A0A146UX93
Down Genes	eif4enif1	A0A146ZY27
	elavl2	A0A1A8LH22
Down Proteins	aldh6a1	H2M098
	LOC103386097	H2MEF1
	gpt	H3CRT8
	LOC107988558	H3CVU7
	zar1	I3J4P9
	ybx2	I3KRX0
	LOC103377398	Q4RMZ0
Up Genes	LOC103383309	A0A1A7WHV4
Down Proteins	LOC103377896	A0A1A8EVF9
	hspd1	A0ELV8
Down Genes	LOC103393907	D9I8D0
Up Proteins	LOC103393908	D9I8D0
	LOC103376887	E6ZII0

**Notes.**

Up and down refers to the expression in pseudomales compared with females.

### Integrative analysis of transcriptomic and proteomic results

A total of 52 genes were differentially expressed between females and pseudomales at both the transcriptomic and proteomic levels. Further correlation analysis for these 52 genes revealed that *ρ* = 0.59 (*p* < 0.05) for the mRNA–protein correlations. Among these 52 related genes, 46 DEGs (88%) were well matched in their levels of protein abundance change. Additionally, compared to females, 33 genes were up-regulated in pseudomales, based on both transcriptomic and proteomic analysis, including YOD1 deubiquitinase (*yod1*), ubiquitin recognition factor in ER associated degradation 1 (*ufd1*), MINDY lysine 48 deubiquitinase 3 (*mindy3*), ubiquitin like domain containing CTD phosphatase 1 (*ublcp1*) and ADP ribosylation factor like GTPase 1 (*arl1*), importin 4 (*ipo4*), microtubule associated protein 1 light chain 3 alpha (*map1lc3a*). Transcriptomic and proteomic analysis both indicated the down-regulation of 13 genes, including Rho GDP dissociation inhibitor alpha (*arhgdia*), neural cell adhesion molecule 1-like (LOC103395384), eukaryotic translation initiation factor 4E nuclear import factor 1 (*eif4enif1*), aldehyde dehydrogenase 6 family member A1 (*aldh6a1*), and zygote arrest 1 (*zar1*). Six genes displayed decoupling patterns between the transcriptome and the proteome. Both LOC103376887 and heat shock 60 protein 1 (*hspd1*) were up-regulated in proteome analysis while the corresponding DEGs were down-regulated at transcript level. Creatine kinase M-type (LOC103377896) and sarcoplasmic/endoplasmic reticulum calcium ATPase 2-like (LOC103383309) were both down-regulated at the translation level but up-regulated at the transcriptomic level (Table 3). GO function analysis found that *arl1*, *map1lc3a*, and *yod1* were enriched in cytosolic component while *arl1*, *ipo4*, *hspd1*, *arhgdia*, and *zar1* were enriched in the cytoplasm component.

## Discussion

Sex development is a complex process, consisting of sex determination, initiation, differentiation and maintenance, ultimately producing sperm or eggs for germline transmission (Zhang et al., 2016). In this study, we performed integrative analysis of the transcriptomes and proteomes of gonadal tissues of *C. semilaevis* females and pseudomales. Our findings suggested that *dmrt1*, *dmrt3*, and *foxl2* showed differential expression after *C. semilaevis* reversal, which may be related to the maintenance of the gonad phenotype after sexual reversion. Studies have shown that males with *dmrt1* mutations form ovary-like testes while expression of the female-related gene *foxl2* was significantly increased in the gonads of these mutants (Cui et al., 2017). In addition, abundant *dmrt3* expression has been detected in adult *Takifugu rubripes* testes, with lower levels of ovarian expression (Yamaguchi et al., 2006). Expression of *dmrt3* has been detected in developing sperm cells in the testes and in developing oocytes in the ovaries of *Monopterus albus* (Sheng et al., 2014). This evidences collectively demonstrates that *dmrt1* and *dmrt3* may be associated with gonadal development in *C. semilaevis*, which was consistent with previous studies (Wang et al., 2019; Dong, Chen & Ji, 2010). *Foxl2* is a critical factor essential for ovary differentiation and maintenance, which is also a depressor for the genetic program for somatic testis determination (Uhlenhaut et al., 2009). In other studies, *foxl2* expression was significantly higher in ovaries compared to other tissues, so it was deduced that the *foxl2* regulates early differentiation of the ovaries in reproduction via the hypothalamus-pituitary-gonad axis and its function is conserved (Lyu et al., 2019). ZP4 is component of the zona pellucida, an extracellular matrix surrounding oocytes which mediates sperm binding, may act as a sperm receptor. Down-regulation *foxl2* and ZP4, such “female-specific” genes in pseudomales, also showed the sex-reversal from female to male occured at transcript level. Recent study reported that the methylation level of *cyp19a1a* promoter was higher in gonads in females than in males and pseudomales, suggested that *cyp19a1a* might be the mechanism for the temperature induced masculinization in *C. semilaevis* (Liu et al., 2019). However, the *cyp19a1a* gene was not found in our data, which might be due to its low expression level at certain conditions.

The *wnt* signaling pathway is a conservative signaling network involved in embryonic development, cell differentiation and proliferation, and growth regulation. Our findings indicate that female highly expressed genes are enriched to the *wnt* signaling pathway. *Wnt* is a secreted glycoprotein, and of which different subtypes have been found in animals (Gao et al., 2014). *Wnt4* is regarded as a sex determination gene that plays a key role in the morphological development of female mammals (Jordan et al., 2001). *Wnt4* regulates müllerian duct formation and the generation of ovarian steroids (Biason-Lauber et al., 2004). Due to our strict differential genetic selection criteria, *wnt4* was not identified as a differentially expressed gene in our results. However, querying its expression in transcriptome data found that it is only expressed in females and that *wnt4* mRNA levels in pseudomales are close to zero. In this study, *wnt9b* was differentially expressed based on our transcriptome data is clearly enriched in the *wnt* signaling pathway, indicating a regulatory role. Additionally, *wnt9b* plays a role of organizing signal in the regulation of the mammalian urogenital system (Carroll et al., 2005). Further research is required to elucidate the roles of these genes in the reproductive process of *C. semilaevis*.

The most important biological processes in the testis and ovaries are spermatogenesis and oogenesis, respectively. As expected, differentially expressed genes we identified include genes associated with oocyte generation stage. The enrichment of progesterone-mediated oocyte maturation and oocyte meiosis pathways in KEGG pathway analysis of the transcriptomes indicated that the gonad function has changed after sex reversal. Previously, sectioning ZW fish revealed chimeric gonads before completion of sex reversal was completed, with normal male gonads present after reversal was complete (Jiang & Li, 2017).

In this study, four ubiquitin-related genes *yod1*, *ufd1*, *mindy3* and *ublcp1* were found to be up-regulated in pseudomales and the corresponding proteins were also up-regulated. In addition, pseudo-highly expressed proteins were also significantly enriched in the ubiquitin-mediated proteolytic pathway. Ubiquitin is a small molecular weight protein commonly found in eukaryotic cells. It participates in the selective degradation of most proteins, also plays an important role in various cell life activities, such as signal transduction, immunity response, transcription and translation (Hershko & Ciechanover, 1998). Therefore, we speculated that protein ubiquitination may be related to some changes in gonad development after sexual reversal. Several studies have analyzed several sperm quality measurements and confirmed their association with ubiquitination (Sutovsky, Terada & Schatten, 2001; Peter et al., 2003; Christopher, Rex & Gary, 2004; Muratori et al., 2005; Ozanon, Chouteau & Sutovsky, 2005; Lovercamp et al., 2008). In addition, the number of ubiquitinated sperm in semen samples is related to infertility or poor sperm quality in humans (Sutovsky, Terada & Schatten, 2001; Ozanon, Chouteau & Sutovsky, 2005), stallions (Peter et al., 2003), bulls (Peter, Evelyn & Gerald, 2002) and boar (Christopher, Rex & Gary, 2004; Lovercamp et al., 2008). The potential effect of ubiquitination on sexual reversal in *C. semilaevis* needs to be investigated in the future.

Recently, genomics and proteomics studies have shown that although proteins are translated from mRNA, the levels of protein and mRNA are not well correlated (Greenbaum et al., 2003);). Therefore, protein levels cannot be reliably predicted from gene expression. Consistent with previous findings, mRNA and protein correlations were also moderate in this study (Spearman correlation coefficients were 0.59). As previously discussed, only a small fraction of corresponding mRNA and protein pairs were identified in transcriptome and proteome correlation analysis, moreover, 6 genes showed inconsistencies in transcription and protein levels. These results suggest that changes in mRNA levels provide limited insight into changes in protein expression, and this result builds an evidence that mRNA expression is not sufficient to fully understand expression changes. The discrepancies between mRNA and protein may be related to complex post-transcriptional regulatory mechanisms, different kinetics of RNA or protein degradation, and detection limits or biases of instruments that measure mRNA and protein, among other origins (Li et al., 2018).

## Conclusions

RNA-Seq revealed that 1893 genes, including the sex-related genes *dmrt1*, *dmrt3* and *foxl2* were found to be differentially expressed between pseudomales and females. At the transcription level, female high-expression genes are mainly enriched in terms of DNA replication initiation, wnt signaling pathways, cell development and oocyte maturation, and enriched in pathways of cell cycle, progesterone-mediated oocyte maturation and oocyte meiosis. The pseudomale-highly expressed genes are mainly enriched in autophagy and cytoplasm term, and adipocytokine signaling pathway and metabolic pathway. At the protein level, cytoplasmic mRNA processing body assembly, RNA processing and protein polymerization are the top three terms female with high expression of DEP enrichment. RNA degradation, spliceosome and metabolic pathways are the top three pathways female with high expression of DEP enrichment. However, regulation of DNA-templated transcription, elongation is the high expression of proteins in pseudomales enrich the most significant term. Protein processing in endoplasmic reticulum, PPAR signaling pathway and fatty acid biosynthesis were the top three pathways of the pseudomale highly expressed proteins.

##  Supplemental Information

10.7717/peerj.8801/supp-1 Figure S1 Female gonadal section pictures

10.7717/peerj.8801/supp-2 Figure S2 Pseudomale gonadal section pictures

10.7717/peerj.8801/supp-3 Table S1 List of all differentially expressed genes

10.7717/peerj.8801/supp-4 Table S2 List of all differentially expressed proteins

10.7717/peerj.8801/supp-5 Supplemental Information 1 Running code in Linux

10.7717/peerj.8801/supp-6 Supplemental Information 2 Running code in R

10.7717/peerj.8801/supp-7 Supplemental Information 3 Sample ID

## Additional Information and Declarations

### DNA Deposition

The following information was supplied regarding the deposition of DNA sequences: The raw data of transcriptomes and proteomics obtained in this bioproject (PRJNA586726) are available at Genbank: SRR10492824, SRR10492825, SRR10492826, SRR10492827, SRR10492828, SRR10492829, SRR10492830, SRR10492831.

### Data Availability

The following information was supplied regarding data availability: The data is available at Zenodo: Ye, Zhan. (2020). sex reversal transcriptomic analysis in Chinese tongue sole [Data set]. Zenodo. http://doi.org/10.5281/zenodo.3507269.
