# Supplementary figures and images for: Integrative analysis reveals pathways associated with sex reversal in Cynoglossus semilaevis
Source: PeerJ. 2020 Mar 19;8:e8801. doi: 10.7717/peerj.8801 (PMC7085895; doi:10.7717/peerj.8801)

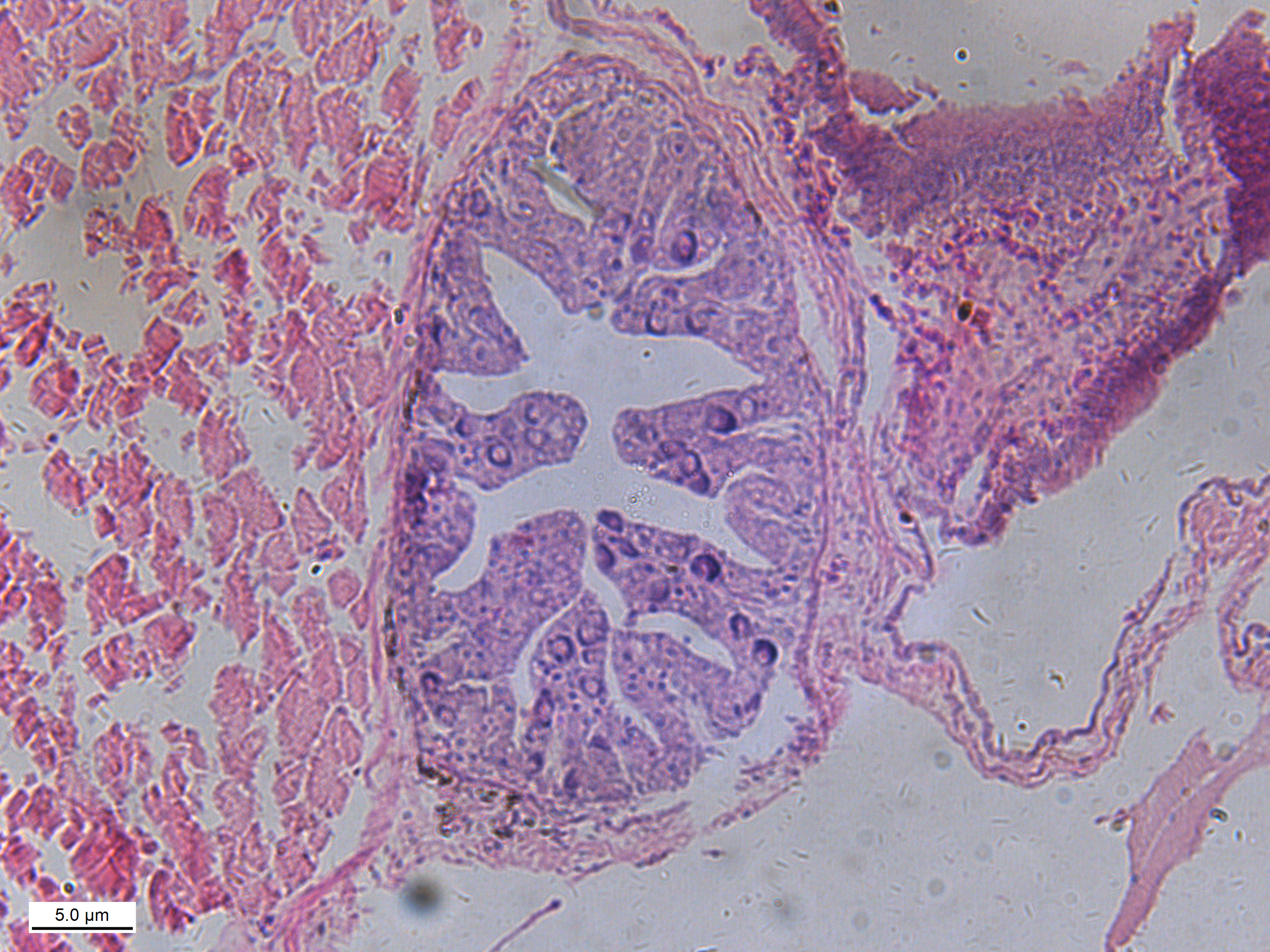

Supplement: Figure S1 [file peerj-08-8801-s001.png]

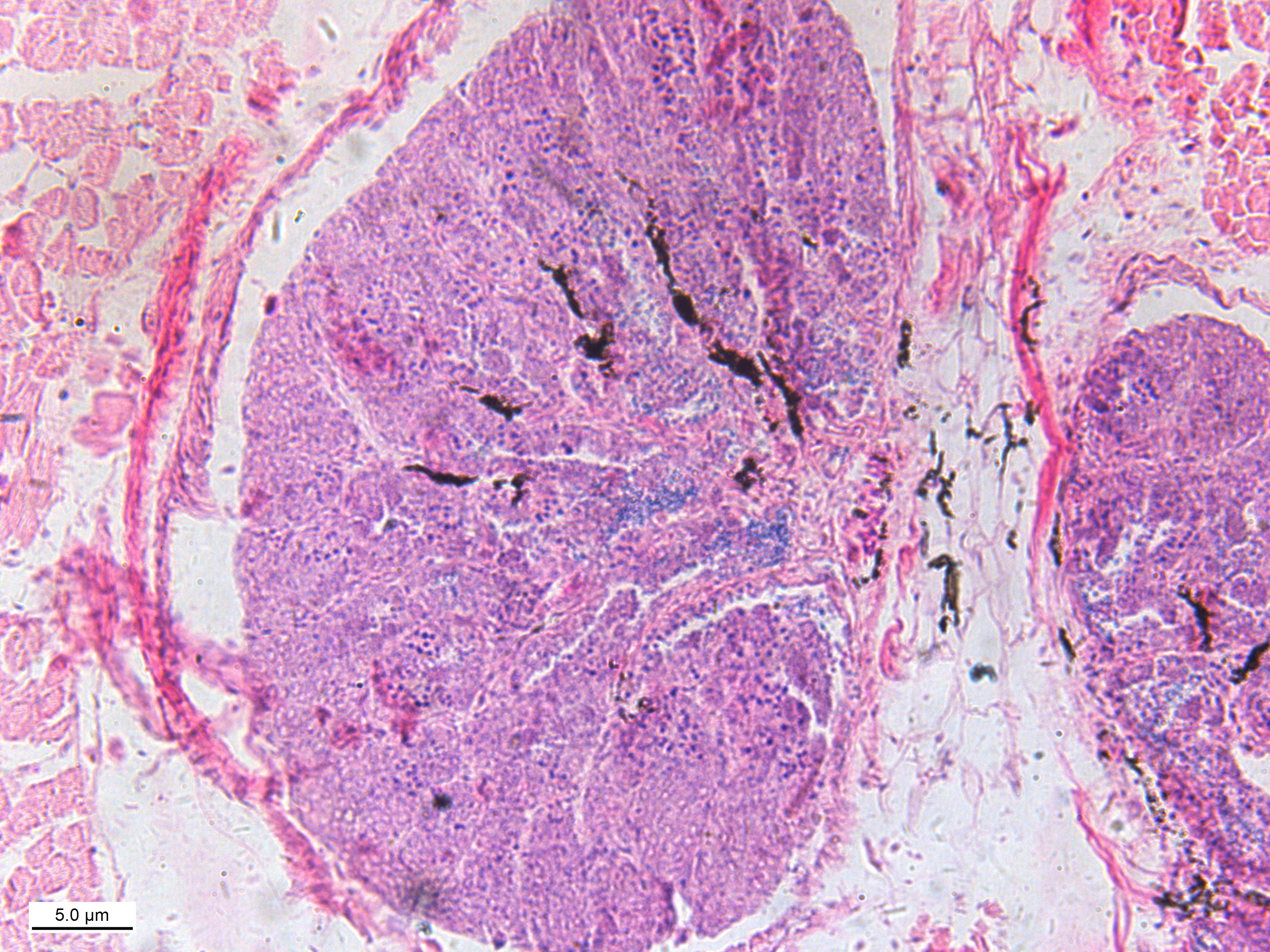

Supplement: Figure S2 [file peerj-08-8801-s002.png]
