# Supplementary material for: Integrative analysis reveals pathways associated with sex reversal in Cynoglossus semilaevis
Source: PeerJ. 2020 Mar 19;8:e8801. doi: 10.7717/peerj.8801 (PMC7085895; doi:10.7717/peerj.8801)
Supplement: Table S1 [file peerj-08-8801-s003.docx]

**Supplement Table S1. List of all differentially expressed genes.**

| Gene.name | fc | pval | qval | ABSlog2fc |
| --- | --- | --- | --- | --- |
| LOC103393374 | 0.035886 | 3.06E-09 | 7.29E-05 | 4.800431 |
| LOC103396071 | 0.000562 | 1.13E-08 | 0.000135 | 10.7964 |
| LOC103382284 | 0.000607 | 4.29E-08 | 0.000159 | 10.68654 |
| LOC103379620 | 0.001034 | 3.15E-08 | 0.000159 | 9.918079 |
| LOC103396600 | 0.001675 | 4.11E-08 | 0.000159 | 9.221361 |
| LOC103382285 | 0.002032 | 4.35E-08 | 0.000159 | 8.942774 |
| LOC103377497 | 0.068807 | 4.66E-08 | 0.000159 | 3.86131 |
| LOC112487260 | 0.000344 | 5.40E-08 | 0.000161 | 11.50511 |
| LOC112487259 | 0.001358 | 6.29E-08 | 0.000167 | 9.524678 |
| LOC103396601 | 0.001484 | 1.00E-07 | 0.000239 | 9.395808 |
| LOC103391153 | 0.061707 | 1.20E-07 | 0.00026 | 4.018429 |
| LOC103392072 | 0.000623 | 1.82E-07 | 0.00031 | 10.64926 |
| LOC103396044 | 0.001464 | 1.75E-07 | 0.00031 | 9.41545 |
| LOC103396043 | 0.002294 | 1.65E-07 | 0.00031 | 8.768068 |
| LOC103396037 | 0.014494 | 2.24E-07 | 0.000334 | 6.108432 |
| LOC103389233 | 0.018 | 2.22E-07 | 0.000334 | 5.795865 |
| LOC103391323 | 18.87425 | 2.43E-07 | 0.000341 | 4.238347 |
| LOC103393113 | 0.000796 | 3.16E-07 | 0.000416 | 10.29569 |
| tmem144 | 0.011368 | 3.31E-07 | 0.000416 | 6.458934 |
| LOC103390914 | 0.000159 | 4.33E-07 | 0.00048 | 12.62275 |
| LOC103391742 | 0.000908 | 6.97E-07 | 0.00048 | 10.10514 |
| LOC103393015 | 0.0013 | 5.16E-07 | 0.00048 | 9.586993 |
| LOC103387799 | 0.003643 | 6.74E-07 | 0.00048 | 8.100671 |
| LOC103392807 | 0.00691 | 5.87E-07 | 0.00048 | 7.177064 |
| LOC103377069 | 0.007134 | 5.88E-07 | 0.00048 | 7.131173 |
| gtf3a | 0.007389 | 5.50E-07 | 0.00048 | 7.080316 |
| LOC103394278 | 0.009208 | 5.16E-07 | 0.00048 | 6.762866 |
| LOC103386097 | 0.012401 | 6.35E-07 | 0.00048 | 6.333363 |
| LOC103380434 | 0.015795 | 7.65E-07 | 0.00048 | 5.984395 |
| LOC103398248 | 0.02877 | 7.59E-07 | 0.00048 | 5.119273 |
| LOC103376815 | 25.98761 | 7.55E-07 | 0.00048 | 4.699752 |
| LOC103391588 | 0.051733 | 7.55E-07 | 0.00048 | 4.272765 |
| LOC103380615 | 18.62011 | 6.85E-07 | 0.00048 | 4.218789 |
| LOC112488247 | 0.057473 | 6.95E-07 | 0.00048 | 4.120968 |
| trim67 | 0.067714 | 5.94E-07 | 0.00048 | 3.884401 |
| LOC103383947 | 0.068204 | 4.65E-07 | 0.00048 | 3.874001 |
| draxin | 0.118552 | 5.70E-07 | 0.00048 | 3.076409 |
| LOC103378200 | 5.216184 | 5.17E-07 | 0.00048 | 2.382995 |
| gtf2a1l | 0.013928 | 8.95E-07 | 0.000547 | 6.165833 |
| LOC103391346 | 0.014618 | 9.68E-07 | 0.000577 | 6.09608 |
| LOC103388694 | 0.153444 | 1.06E-06 | 0.000614 | 2.704212 |
| LOC103394674 | 0.000565 | 1.16E-06 | 0.000641 | 10.79002 |
| LOC103396322 | 0.01638 | 1.16E-06 | 0.000641 | 5.931886 |
| LOC103387536 | 0.127932 | 1.21E-06 | 0.000641 | 2.966546 |
| LOC103381485 | 0.037086 | 1.32E-06 | 0.000687 | 4.752986 |
| trnav-uac | 95.52378 | 1.47E-06 | 0.00073 | 6.577788 |
| LOC103397468 | 0.131913 | 1.66E-06 | 0.000807 | 2.922344 |
| ca7 | 0.006606 | 1.74E-06 | 0.000816 | 7.241968 |
| sbspon | 9.937084 | 1.74E-06 | 0.000816 | 3.312823 |
| cystm1 | 53.09867 | 1.80E-06 | 0.000825 | 5.730604 |
| LOC103382154 | 0.001364 | 2.21E-06 | 0.00093 | 9.517774 |
| LOC103396073 | 0.019355 | 2.28E-06 | 0.00093 | 5.691135 |
| LOC103378872 | 0.021197 | 2.11E-06 | 0.00093 | 5.56002 |
| LOC103389891 | 0.024482 | 2.15E-06 | 0.00093 | 5.352157 |
| LOC103397495 | 0.024505 | 2.30E-06 | 0.00093 | 5.350796 |
| LOC103389383 | 0.077207 | 2.26E-06 | 0.00093 | 3.695116 |
| LOC103384831 | 0.109971 | 2.45E-06 | 0.000973 | 3.184811 |
| LOC103390711 | 0.007606 | 2.64E-06 | 0.001029 | 7.038642 |
| LOC103381141 | 0.076141 | 2.67E-06 | 0.001029 | 3.715176 |
| LOC103383647 | 0.007277 | 2.83E-06 | 0.001029 | 7.10242 |
| LOC103395842 | 0.007695 | 2.87E-06 | 0.001029 | 7.021778 |
| LOC103394689 | 127.3721 | 2.84E-06 | 0.001029 | 6.992905 |
| LOC103392682 | 14.00136 | 2.74E-06 | 0.001029 | 3.807496 |
| LOC103381286 | 0.083214 | 2.89E-06 | 0.001029 | 3.587037 |
| LOC103386630 | 0.011176 | 3.08E-06 | 0.001053 | 6.483463 |
| LOC103386267 | 0.056162 | 3.05E-06 | 0.001053 | 4.154251 |
| LOC103397210 | 0.066323 | 3.09E-06 | 0.001053 | 3.914341 |
| LOC103397350 | 0.190148 | 3.55E-06 | 0.001195 | 2.394805 |
| LOC103395009 | 0.001594 | 4.34E-06 | 0.001248 | 9.292784 |
| LOC103380918 | 0.007468 | 4.34E-06 | 0.001248 | 7.065049 |
| LOC103391404 | 131.2274 | 4.26E-06 | 0.001248 | 7.035926 |
| LOC103396640 | 0.01013 | 3.84E-06 | 0.001248 | 6.625161 |
| LOC103391819 | 92.89076 | 4.26E-06 | 0.001248 | 6.537463 |
| sybu | 0.018036 | 4.08E-06 | 0.001248 | 5.792978 |
| LOC103395027 | 0.018218 | 4.20E-06 | 0.001248 | 5.778515 |
| LOC107989869 | 12.91284 | 3.93E-06 | 0.001248 | 3.690735 |
| LOC103399914 | 0.113933 | 3.98E-06 | 0.001248 | 3.133746 |
| poc1a | 5.894542 | 3.88E-06 | 0.001248 | 2.55938 |
| LOC103391713 | 0.204054 | 4.25E-06 | 0.001248 | 2.292976 |
| LOC103384727 | 22.4836 | 4.57E-06 | 0.001297 | 4.490801 |
| LOC103377398 | 0.001728 | 4.67E-06 | 0.00131 | 9.17629 |
| LOC103384281 | 17.09385 | 4.82E-06 | 0.001311 | 4.095406 |
| pkd1l1 | 0.173602 | 4.78E-06 | 0.001311 | 2.526145 |
| LOC103387155 | 0.024239 | 5.11E-06 | 0.001369 | 5.366542 |
| LOC103399958 | 0.073187 | 5.22E-06 | 0.001383 | 3.772275 |
| LOC103397775 | 87.2105 | 5.36E-06 | 0.001386 | 6.44643 |
| LOC103395363 | 0.103971 | 5.31E-06 | 0.001386 | 3.265746 |
| tmem45a | 29.93468 | 5.53E-06 | 0.001404 | 4.903746 |
| LOC103392929 | 0.00187 | 5.70E-06 | 0.001409 | 9.062492 |
| LOC103378409 | 5.715361 | 5.73E-06 | 0.001409 | 2.514845 |
| cd80 | 5.622797 | 5.70E-06 | 0.001409 | 2.491288 |
| LOC103382600 | 0.107425 | 5.85E-06 | 0.001424 | 3.218598 |
| LOC107989798 | 56.54811 | 5.98E-06 | 0.001441 | 5.821407 |
| LOC103383374 | 47.71095 | 6.43E-06 | 0.001462 | 5.576248 |
| zglp1 | 0.11959 | 6.20E-06 | 0.001462 | 3.063833 |
| LOC103392376 | 0.020811 | 6.82E-06 | 0.001509 | 5.586494 |
| LOC103378060 | 0.03507 | 6.83E-06 | 0.001509 | 4.833623 |
| gpx3 | 0.087416 | 6.80E-06 | 0.001509 | 3.515967 |
| LOC103395618 | 86.17235 | 7.29E-06 | 0.001595 | 6.429153 |
| LOC103384567 | 69.02418 | 7.56E-06 | 0.001625 | 6.10903 |
| LOC103377052 | 106.632 | 8.16E-06 | 0.001645 | 6.736497 |
| pck2 | 0.018615 | 8.15E-06 | 0.001645 | 5.747388 |
| LOC103396379 | 0.106958 | 7.90E-06 | 0.001645 | 3.22489 |
| LOC103393777 | 6.151787 | 8.19E-06 | 0.001645 | 2.621006 |
| LOC103392083 | 0.242714 | 8.13E-06 | 0.001645 | 2.042671 |
| LOC107988995 | 110.0421 | 8.49E-06 | 0.001687 | 6.781912 |
| LOC103383835 | 0.045013 | 8.56E-06 | 0.001687 | 4.473512 |
| acsl5 | 0.006326 | 8.89E-06 | 0.001704 | 7.304473 |
| ndp | 0.190417 | 8.93E-06 | 0.001704 | 2.392766 |
| LOC103395381 | 0.230336 | 8.82E-06 | 0.001704 | 2.118188 |
| LOC103384121 | 0.002582 | 9.04E-06 | 0.001712 | 8.597386 |
| LOC112488713 | 7.833052 | 9.52E-06 | 0.001774 | 2.969575 |
| LOC103382844 | 43.59831 | 9.94E-06 | 0.001778 | 5.4462 |
| LOC103389361 | 0.066188 | 9.80E-06 | 0.001778 | 3.917278 |
| penk | 0.080593 | 9.81E-06 | 0.001778 | 3.633203 |
| LOC103388987 | 115.5769 | 1.02E-05 | 0.001783 | 6.852709 |
| arg2 | 4.50103 | 1.01E-05 | 0.001783 | 2.170255 |
| LOC103385943 | 20.19108 | 1.02E-05 | 0.001785 | 4.335646 |
| LOC103396761 | 53.72597 | 1.04E-05 | 0.001785 | 5.747548 |
| LOC103391083 | 33.44333 | 1.05E-05 | 0.001785 | 5.063647 |
| LOC103391230 | 0.106251 | 1.09E-05 | 0.001841 | 3.234448 |
| LOC103390804 | 8.422561 | 1.13E-05 | 0.001869 | 3.074259 |
| LOC103390506 | 87.33144 | 1.23E-05 | 0.001921 | 6.448429 |
| galnt17 | 0.032323 | 1.24E-05 | 0.001921 | 4.951294 |
| LOC103381359 | 0.088435 | 1.23E-05 | 0.001921 | 3.499246 |
| slc5a11 | 10.3153 | 1.23E-05 | 0.001921 | 3.366714 |
| LOC103391040 | 0.108724 | 1.26E-05 | 0.001932 | 3.201258 |
| LOC103388389 | 0.005264 | 1.29E-05 | 0.001965 | 7.569585 |
| LOC103385655 | 116.8622 | 1.36E-05 | 0.001981 | 6.868664 |
| aqp1 | 82.5014 | 1.42E-05 | 0.001981 | 6.366347 |
| LOC103392997 | 75.48844 | 1.43E-05 | 0.001981 | 6.238184 |
| LOC103379210 | 67.1077 | 1.44E-05 | 0.001981 | 6.068406 |
| LOC103388935 | 46.22654 | 1.35E-05 | 0.001981 | 5.530649 |
| LOC103380033 | 0.041392 | 1.37E-05 | 0.001981 | 4.59449 |
| LOC103395698 | 20.61387 | 1.43E-05 | 0.001981 | 4.365544 |
| enkd1 | 13.32549 | 1.33E-05 | 0.001981 | 3.736117 |
| rchy1 | 8.436187 | 1.40E-05 | 0.001981 | 3.076591 |
| LOC112487247 | 0.013573 | 1.50E-05 | 0.001996 | 6.203108 |
| LOC103390503 | 68.87503 | 1.47E-05 | 0.001996 | 6.105909 |
| LOC103378763 | 41.82564 | 1.48E-05 | 0.001996 | 5.386316 |
| LOC103388692 | 0.023914 | 1.49E-05 | 0.001996 | 5.385972 |
| LOC103391903 | 0.029821 | 1.51E-05 | 0.001996 | 5.067514 |
| dpp7 | 6.255813 | 1.51E-05 | 0.001996 | 2.645197 |
| mpv17l | 5.530075 | 1.51E-05 | 0.001996 | 2.467299 |
| LOC103388350 | 46.81425 | 1.55E-05 | 0.002034 | 5.548876 |
| tex36 | 60.25745 | 1.61E-05 | 0.002078 | 5.913068 |
| LOC103386644 | 0.08277 | 1.65E-05 | 0.002078 | 3.594756 |
| tbx1 | 6.837 | 1.65E-05 | 0.002078 | 2.773363 |
| zar1 | 0.003823 | 1.69E-05 | 0.002089 | 8.031043 |
| s100b | 34.42086 | 1.70E-05 | 0.002089 | 5.105211 |
| LOC103390446 | 0.038844 | 1.68E-05 | 0.002089 | 4.686151 |
| LOC103379533 | 0.070273 | 1.69E-05 | 0.002089 | 3.830879 |
| rec8 | 46.92164 | 1.73E-05 | 0.00212 | 5.552182 |
| LOC103395318 | 19.63837 | 1.76E-05 | 0.002138 | 4.295603 |
| LOC103380998 | 39.4235 | 1.77E-05 | 0.002147 | 5.300984 |
| LOC112487099 | 18.53232 | 1.78E-05 | 0.002147 | 4.211971 |
| LOC103388986 | 54.20964 | 1.80E-05 | 0.002158 | 5.760478 |
| LOC103381617 | 6.082873 | 1.82E-05 | 0.00217 | 2.604753 |
| LOC103382861 | 65.75596 | 1.84E-05 | 0.002178 | 6.03905 |
| LOC103380559 | 64.56626 | 1.86E-05 | 0.002182 | 6.012708 |
| LOC103391796 | 24.23186 | 1.86E-05 | 0.002182 | 4.598833 |
| mkrn2os | 13.21753 | 1.87E-05 | 0.002182 | 3.724381 |
| pus3 | 0.088815 | 1.87E-05 | 0.002182 | 3.49305 |
| LOC103383786 | 0.012471 | 1.91E-05 | 0.002199 | 6.325266 |
| LOC107988500 | 21.94124 | 1.91E-05 | 0.002199 | 4.455573 |
| LOC103384013 | 0.008479 | 2.01E-05 | 0.0022 | 6.881826 |
| LOC107989065 | 108.9862 | 1.95E-05 | 0.0022 | 6.768002 |
| LOC103393514 | 101.2977 | 2.02E-05 | 0.0022 | 6.662458 |
| LOC103391143 | 64.8092 | 2.04E-05 | 0.0022 | 6.018127 |
| LOC103388884 | 64.13222 | 1.98E-05 | 0.0022 | 6.002978 |
| LOC103398791 | 49.81001 | 1.92E-05 | 0.0022 | 5.638364 |
| LOC103384137 | 43.72187 | 1.98E-05 | 0.0022 | 5.450283 |
| LOC103397579 | 0.033649 | 1.97E-05 | 0.0022 | 4.893307 |
| LOC112486367 | 23.13292 | 2.03E-05 | 0.0022 | 4.531876 |
| LOC103389105 | 7.654147 | 2.01E-05 | 0.0022 | 2.936242 |
| LOC103391428 | 80.85678 | 2.05E-05 | 0.002202 | 6.337297 |
| LOC107989043 | 37.11425 | 2.07E-05 | 0.002202 | 5.213901 |
| LOC103389561 | 12.19251 | 2.10E-05 | 0.002213 | 3.607924 |
| LOC103396063 | 0.037676 | 2.12E-05 | 0.002228 | 4.730216 |
| LOC103386482 | 51.11595 | 2.14E-05 | 0.002232 | 5.675702 |
| LOC103396348 | 15.13195 | 2.19E-05 | 0.002271 | 3.919526 |
| tnmd | 0.1615 | 2.21E-05 | 0.00228 | 2.630393 |
| LOC103390521 | 126.4416 | 2.26E-05 | 0.002288 | 6.982327 |
| LOC103393151 | 55.87986 | 2.24E-05 | 0.002288 | 5.804256 |
| LOC103391672 | 28.62742 | 2.26E-05 | 0.002288 | 4.839326 |
| ppm1b | 6.738011 | 2.26E-05 | 0.002288 | 2.752323 |
| LOC103396760 | 79.83047 | 2.28E-05 | 0.002294 | 6.318868 |
| LOC107989417 | 146.7568 | 2.30E-05 | 0.002297 | 7.197284 |
| LOC103394101 | 0.12415 | 2.31E-05 | 0.002297 | 3.009841 |
| LOC103389210 | 0.019913 | 2.34E-05 | 0.002302 | 5.650139 |
| igf2bp3 | 0.035197 | 2.37E-05 | 0.002319 | 4.828392 |
| LOC103397452 | 0.047627 | 2.38E-05 | 0.00232 | 4.392072 |
| LOC103398059 | 0.005758 | 2.43E-05 | 0.002335 | 7.440112 |
| LOC103377641 | 0.012007 | 2.43E-05 | 0.002335 | 6.380019 |
| kctd6 | 0.156059 | 2.45E-05 | 0.00234 | 2.679836 |
| map1lc3c | 0.00409 | 2.52E-05 | 0.002349 | 7.933767 |
| LOC103388164 | 0.005327 | 2.61E-05 | 0.002349 | 7.552552 |
| LOC103394325 | 95.45225 | 2.59E-05 | 0.002349 | 6.576707 |
| LOC103399210 | 57.8085 | 2.59E-05 | 0.002349 | 5.85321 |
| LOC103378054 | 0.018799 | 2.54E-05 | 0.002349 | 5.733204 |
| LOC103395333 | 50.34365 | 2.49E-05 | 0.002349 | 5.653738 |
| LOC112488011 | 41.98072 | 2.53E-05 | 0.002349 | 5.391655 |
| cabcoco1 | 26.6619 | 2.52E-05 | 0.002349 | 4.736708 |
| LOC103382299 | 12.28852 | 2.61E-05 | 0.002349 | 3.61924 |
| tmed6 | 9.579937 | 2.50E-05 | 0.002349 | 3.260016 |
| lnp1 | 4.554922 | 2.55E-05 | 0.002349 | 2.187426 |
| LOC103391454 | 115.9185 | 2.67E-05 | 0.002361 | 6.856967 |
| pabpn1l | 0.010702 | 2.68E-05 | 0.002361 | 6.545991 |
| LOC103393178 | 88.05918 | 2.65E-05 | 0.002361 | 6.460402 |
| LOC103397700 | 0.0322 | 2.68E-05 | 0.002361 | 4.956781 |
| LOC103388713 | 0.215196 | 2.70E-05 | 0.00237 | 2.216274 |
| LOC103378000 | 0.120403 | 2.71E-05 | 0.00237 | 3.05406 |
| LOC103378861 | 144.3938 | 2.84E-05 | 0.002382 | 7.173865 |
| LOC103382200 | 124.199 | 2.77E-05 | 0.002382 | 6.95651 |
| LOC103384835 | 94.84113 | 2.79E-05 | 0.002382 | 6.567441 |
| LOC103386538 | 80.46379 | 2.78E-05 | 0.002382 | 6.330268 |
| LOC103378930 | 51.02689 | 2.76E-05 | 0.002382 | 5.673186 |
| LOC103395317 | 39.80312 | 2.83E-05 | 0.002382 | 5.31481 |
| aspdh | 15.04708 | 2.75E-05 | 0.002382 | 3.911411 |
| LOC103385488 | 0.090167 | 2.81E-05 | 0.002382 | 3.471262 |
| tmem44 | 0.165038 | 2.80E-05 | 0.002382 | 2.599128 |
| cnih1 | 0.230499 | 2.83E-05 | 0.002382 | 2.11717 |
| LOC103393902 | 26.06627 | 2.85E-05 | 0.002388 | 4.704112 |
| LOC103395003 | 0.18086 | 2.88E-05 | 0.002402 | 2.467058 |
| LOC103380964 | 67.89284 | 2.90E-05 | 0.002405 | 6.085188 |
| LOC103387780 | 75.63297 | 2.92E-05 | 0.002413 | 6.240943 |
| LOC107988307 | 60.49943 | 2.96E-05 | 0.002433 | 5.91885 |
| fam221a | 49.01546 | 2.99E-05 | 0.002443 | 5.615165 |
| LOC103394960 | 11.87693 | 3.02E-05 | 0.002449 | 3.57009 |
| LOC103387360 | 21.67258 | 3.07E-05 | 0.002482 | 4.437799 |
| LOC103391468 | 9.548453 | 3.08E-05 | 0.002482 | 3.255267 |
| cidec | 0.022115 | 3.13E-05 | 0.002486 | 5.498863 |
| LOC103377438 | 6.9073 | 3.11E-05 | 0.002486 | 2.788122 |
| pcf11 | 8.263136 | 3.16E-05 | 0.002502 | 3.046689 |
| LOC103389267 | 0.138173 | 3.17E-05 | 0.002502 | 2.855447 |
| LOC103395369 | 44.26914 | 3.19E-05 | 0.002504 | 5.468229 |
| LOC103389481 | 0.030169 | 3.23E-05 | 0.002504 | 5.050812 |
| LOC103382308 | 27.50544 | 3.22E-05 | 0.002504 | 4.781645 |
| LOC103379373 | 8.536803 | 3.23E-05 | 0.002504 | 3.093696 |
| LOC103384852 | 11.93978 | 3.28E-05 | 0.002529 | 3.577704 |
| LOC107990206 | 0.164768 | 3.30E-05 | 0.002536 | 2.601491 |
| LOC103393531 | 20.88656 | 3.33E-05 | 0.002541 | 4.384503 |
| LOC103383083 | 19.1424 | 3.33E-05 | 0.002541 | 4.2587 |
| LOC103396789 | 70.58124 | 3.36E-05 | 0.002545 | 6.141213 |
| LOC103383017 | 17.52733 | 3.38E-05 | 0.002546 | 4.131535 |
| LOC103387404 | 0.090993 | 3.38E-05 | 0.002546 | 3.458104 |
| LOC103383427 | 27.96453 | 3.42E-05 | 0.002569 | 4.805526 |
| LOC103377291 | 88.10796 | 3.47E-05 | 0.002576 | 6.4612 |
| LOC103384383 | 24.13695 | 3.46E-05 | 0.002576 | 4.593172 |
| uhrf1bp1l | 4.67514 | 3.46E-05 | 0.002576 | 2.22501 |
| LOC103392871 | 23.47989 | 3.48E-05 | 0.002577 | 4.553354 |
| dmrt3 | 16.82237 | 3.53E-05 | 0.002599 | 4.072309 |
| LOC103382727 | 8.966882 | 3.53E-05 | 0.002599 | 3.164606 |
| fbxo21 | 0.212547 | 3.55E-05 | 0.002599 | 2.234149 |
| LOC103385171 | 0.244346 | 3.54E-05 | 0.002599 | 2.033004 |
| hoxa5 | 0.106838 | 3.57E-05 | 0.002603 | 3.226501 |
| LOC103395749 | 35.40451 | 3.58E-05 | 0.002607 | 5.145861 |
| LOC107988993 | 84.21601 | 3.62E-05 | 0.002608 | 6.396023 |
| pck1 | 55.46132 | 3.65E-05 | 0.002608 | 5.79341 |
| LOC112487628 | 15.60357 | 3.64E-05 | 0.002608 | 3.963804 |
| LOC103397885 | 0.163083 | 3.62E-05 | 0.002608 | 2.616326 |
| dapk3 | 0.174795 | 3.63E-05 | 0.002608 | 2.516268 |
| LOC103391375 | 23.59246 | 3.68E-05 | 0.002616 | 4.560254 |
| LOC103388668 | 22.13649 | 3.68E-05 | 0.002616 | 4.468355 |
| LOC103394432 | 27.572 | 3.71E-05 | 0.002624 | 4.785132 |
| LOC112487149 | 197.4173 | 3.73E-05 | 0.002631 | 7.625105 |
| LOC103394193 | 0.088173 | 3.76E-05 | 0.002632 | 3.503517 |
| LOC103384893 | 8.639309 | 3.76E-05 | 0.002632 | 3.110916 |
| hhipl2 | 6.783427 | 3.75E-05 | 0.002632 | 2.762014 |
| LOC103387438 | 133.7456 | 3.82E-05 | 0.002662 | 7.063348 |
| gdf3 | 0.022461 | 3.83E-05 | 0.002662 | 5.476427 |
| LOC103397273 | 0.096541 | 3.85E-05 | 0.002667 | 3.372713 |
| LOC103390513 | 41.3223 | 3.95E-05 | 0.002703 | 5.368849 |
| LOC103389846 | 16.61653 | 3.97E-05 | 0.002703 | 4.054548 |
| LOC103391923 | 0.108602 | 3.97E-05 | 0.002703 | 3.202874 |
| LOC103376786 | 0.11458 | 3.96E-05 | 0.002703 | 3.125578 |
| LOC103379479 | 0.049542 | 4.01E-05 | 0.002716 | 4.335192 |
| ccno | 0.061816 | 4.00E-05 | 0.002716 | 4.015866 |
| LOC103396002 | 0.229973 | 4.02E-05 | 0.002716 | 2.120464 |
| LOC103376855 | 131.6343 | 4.05E-05 | 0.00273 | 7.040392 |
| LOC103394264 | 0.009506 | 4.16E-05 | 0.002739 | 6.716974 |
| LOC103384035 | 91.3211 | 4.17E-05 | 0.002739 | 6.512876 |
| LOC103388805 | 62.00084 | 4.11E-05 | 0.002739 | 5.954216 |
| LOC103386553 | 38.76721 | 4.12E-05 | 0.002739 | 5.276765 |
| LOC103391082 | 30.95683 | 4.16E-05 | 0.002739 | 4.952186 |
| LOC103390389 | 29.96007 | 4.11E-05 | 0.002739 | 4.904969 |
| cntd2 | 0.046475 | 4.13E-05 | 0.002739 | 4.427387 |
| LOC103387688 | 14.45794 | 4.09E-05 | 0.002739 | 3.853791 |
| amz2 | 14.25652 | 4.18E-05 | 0.002739 | 3.83355 |
| LOC103389006 | 21.28455 | 4.21E-05 | 0.002754 | 4.411734 |
| LOC112486759 | 16.62461 | 4.25E-05 | 0.002769 | 4.055248 |
| LOC103384863 | 43.20435 | 4.27E-05 | 0.002774 | 5.433105 |
| LOC103383426 | 23.17567 | 4.29E-05 | 0.002779 | 4.534539 |
| LOC103395664 | 0.018996 | 4.41E-05 | 0.002798 | 5.718174 |
| LOC103385240 | 32.68043 | 4.41E-05 | 0.002798 | 5.030355 |
| LOC103396029 | 0.035221 | 4.35E-05 | 0.002798 | 4.827405 |
| fscn1 | 0.175701 | 4.38E-05 | 0.002798 | 2.508804 |
| LOC103384525 | 0.169291 | 4.47E-05 | 0.002823 | 2.562421 |
| LOC112487134 | 55.34794 | 4.49E-05 | 0.002826 | 5.790458 |
| LOC103397272 | 0.155826 | 4.50E-05 | 0.002826 | 2.681993 |
| znf511 | 7.013911 | 4.53E-05 | 0.002836 | 2.810219 |
| hsd17b12 | 0.07779 | 4.54E-05 | 0.002837 | 3.684267 |
| LOC103379808 | 12.00054 | 4.57E-05 | 0.00285 | 3.585028 |
| LOC103395150 | 0.045177 | 4.70E-05 | 0.002906 | 4.468264 |
| LOC103392036 | 0.089851 | 4.70E-05 | 0.002906 | 3.476323 |
| LOC103386352 | 55.40178 | 4.85E-05 | 0.002972 | 5.79186 |
| col16a1 | 0.230876 | 4.85E-05 | 0.002972 | 2.114807 |
| cep162 | 0.08749 | 4.90E-05 | 0.002982 | 3.514736 |
| timm29 | 0.196528 | 4.91E-05 | 0.002982 | 2.347194 |
| LOC103391398 | 71.73959 | 4.96E-05 | 0.003001 | 6.164698 |
| foxl2 | 0.032555 | 4.97E-05 | 0.003001 | 4.940962 |
| LOC103398372 | 23.23192 | 4.99E-05 | 0.003001 | 4.538036 |
| col22a1 | 0.211171 | 4.99E-05 | 0.003001 | 2.243513 |
| dgat2 | 0.047485 | 5.06E-05 | 0.003024 | 4.396397 |
| LOC107988994 | 88.13137 | 5.08E-05 | 0.003029 | 6.461584 |
| LOC112486458 | 27.42496 | 5.14E-05 | 0.003056 | 4.777418 |
| trnad-guc | 38.2337 | 5.19E-05 | 0.003079 | 5.256773 |
| LOC103387396 | 8.283798 | 5.23E-05 | 0.003082 | 3.050292 |
| LOC103398062 | 6.491034 | 5.27E-05 | 0.0031 | 2.698448 |
| LOC107988876 | 25.34686 | 5.34E-05 | 0.003125 | 4.663735 |
| chd1l | 12.5641 | 5.40E-05 | 0.00315 | 3.651236 |
| LOC103394213 | 0.040164 | 5.43E-05 | 0.003153 | 4.637937 |
| asb7 | 8.123317 | 5.55E-05 | 0.003205 | 3.022069 |
| LOC103379554 | 0.008531 | 5.70E-05 | 0.003221 | 6.873152 |
| LOC103383425 | 63.0881 | 5.61E-05 | 0.003221 | 5.979296 |
| LOC103386335 | 36.01769 | 5.89E-05 | 0.003221 | 5.170634 |
| LOC103377801 | 30.16846 | 5.74E-05 | 0.003221 | 4.914969 |
| LOC103392952 | 21.79759 | 5.76E-05 | 0.003221 | 4.446097 |
| LOC103391245 | 18.21403 | 5.85E-05 | 0.003221 | 4.186978 |
| ybx2 | 0.060873 | 5.86E-05 | 0.003221 | 4.038058 |
| trnam-cau | 13.32863 | 5.79E-05 | 0.003221 | 3.736457 |
| LOC103389324 | 0.105548 | 5.60E-05 | 0.003221 | 3.244026 |
| LOC103390715 | 0.118648 | 5.91E-05 | 0.003221 | 3.075243 |
| LOC103377193 | 7.744194 | 5.77E-05 | 0.003221 | 2.953115 |
| LOC107988508 | 7.545877 | 5.90E-05 | 0.003221 | 2.915689 |
| cdkn2c | 5.953442 | 5.91E-05 | 0.003221 | 2.573724 |
| ppp2r5b | 0.181517 | 5.79E-05 | 0.003221 | 2.461825 |
| LOC103393799 | 4.929117 | 5.81E-05 | 0.003221 | 2.301329 |
| LOC112488398 | 4.813312 | 5.83E-05 | 0.003221 | 2.26703 |
| LOC103382723 | 0.211819 | 5.80E-05 | 0.003221 | 2.239099 |
| LOC103382354 | 0.127561 | 5.96E-05 | 0.003231 | 2.970737 |
| LOC103384067 | 37.01906 | 5.98E-05 | 0.003231 | 5.210197 |
| LOC107989655 | 31.20324 | 6.01E-05 | 0.003231 | 4.963624 |
| LOC107988710 | 20.67634 | 6.01E-05 | 0.003231 | 4.369909 |
| lbh | 0.061451 | 5.99E-05 | 0.003231 | 4.02443 |
| LOC103397476 | 53.5828 | 6.03E-05 | 0.003235 | 5.743698 |
| LOC107989396 | 18.46732 | 6.09E-05 | 0.003259 | 4.206903 |
| LOC112487158 | 25.59026 | 6.11E-05 | 0.003263 | 4.677523 |
| elavl2 | 0.096653 | 6.15E-05 | 0.00327 | 3.37104 |
| pih1d3 | 46.26564 | 6.22E-05 | 0.003289 | 5.531869 |
| cfap99 | 30.9532 | 6.26E-05 | 0.003306 | 4.952017 |
| LOC103385265 | 11.81339 | 6.28E-05 | 0.003307 | 3.562351 |
| LOC103381374 | 16.47855 | 6.30E-05 | 0.003309 | 4.042517 |
| sdk2 | 0.166078 | 6.40E-05 | 0.003351 | 2.590064 |
| dusp10 | 0.037382 | 6.53E-05 | 0.00338 | 4.741506 |
| LOC103394222 | 0.050527 | 6.52E-05 | 0.00338 | 4.306808 |
| LOC103395384 | 0.136352 | 6.51E-05 | 0.00338 | 2.874596 |
| lef1 | 0.190931 | 6.53E-05 | 0.00338 | 2.388879 |
| mospd1 | 6.853017 | 6.62E-05 | 0.00341 | 2.776739 |
| LOC103389872 | 43.9468 | 6.68E-05 | 0.00343 | 5.457686 |
| LOC103394696 | 25.02409 | 6.69E-05 | 0.00343 | 4.645245 |
| col26a1 | 10.47327 | 6.72E-05 | 0.003442 | 3.38864 |
| LOC103393168 | 30.67574 | 6.85E-05 | 0.003466 | 4.939026 |
| LOC103389616 | 27.69062 | 6.85E-05 | 0.003466 | 4.791326 |
| khdrbs3 | 0.056547 | 6.87E-05 | 0.003466 | 4.144411 |
| homer2 | 0.068994 | 6.84E-05 | 0.003466 | 3.857377 |
| LOC103393761 | 13.94873 | 6.89E-05 | 0.003466 | 3.802061 |
| LOC103396602 | 0.07308 | 6.87E-05 | 0.003466 | 3.774372 |
| trhde | 4.409618 | 6.94E-05 | 0.003485 | 2.140654 |
| LOC103393186 | 19.59863 | 7.03E-05 | 0.003508 | 4.292681 |
| LOC103393545 | 62.81204 | 7.17E-05 | 0.003527 | 5.972969 |
| LOC103380361 | 62.0814 | 7.20E-05 | 0.003527 | 5.956089 |
| LOC103384344 | 58.47198 | 7.18E-05 | 0.003527 | 5.869674 |
| LOC107990213 | 42.06429 | 7.23E-05 | 0.003527 | 5.394524 |
| LOC103384885 | 0.177995 | 7.22E-05 | 0.003527 | 2.490091 |
| LOC103393532 | 5.617297 | 7.11E-05 | 0.003527 | 2.489876 |
| LOC103381564 | 0.035251 | 7.30E-05 | 0.003553 | 4.826191 |
| LOC103383075 | 0.00273 | 7.36E-05 | 0.003566 | 8.517068 |
| LOC103386281 | 109.5011 | 7.40E-05 | 0.003566 | 6.774802 |
| LOC107989506 | 26.97916 | 7.38E-05 | 0.003566 | 4.753774 |
| nptn | 0.223337 | 7.36E-05 | 0.003566 | 2.162704 |
| ppil6 | 26.14054 | 7.45E-05 | 0.003569 | 4.708217 |
| LOC103379607 | 4.818674 | 7.45E-05 | 0.003569 | 2.268636 |
| LOC103388973 | 0.066208 | 7.46E-05 | 0.003569 | 3.916846 |
| LOC103378806 | 0.161436 | 7.58E-05 | 0.003612 | 2.630969 |
| LOC103377847 | 0.066794 | 7.65E-05 | 0.003634 | 3.904138 |
| survivin | 0.032208 | 7.78E-05 | 0.003646 | 4.956422 |
| LOC103381809 | 0.058095 | 7.80E-05 | 0.003646 | 4.10545 |
| LOC103397682 | 4.98527 | 7.75E-05 | 0.003646 | 2.317672 |
| nhp2 | 0.227789 | 7.71E-05 | 0.003646 | 2.134228 |
| LOC103382995 | 0.24104 | 7.77E-05 | 0.003646 | 2.052658 |
| LOC103388724 | 21.66051 | 7.85E-05 | 0.003651 | 4.436995 |
| LOC103386141 | 0.071584 | 7.85E-05 | 0.003651 | 3.804226 |
| LOC103388885 | 24.13062 | 7.90E-05 | 0.003666 | 4.592793 |
| LOC103387398 | 63.39856 | 7.98E-05 | 0.003679 | 5.986378 |
| LOC103389030 | 54.20012 | 8.08E-05 | 0.003679 | 5.760224 |
| LOC103391559 | 53.21479 | 8.21E-05 | 0.003679 | 5.733755 |
| LOC103380050 | 31.40987 | 8.16E-05 | 0.003679 | 4.973146 |
| LOC112487256 | 0.033655 | 8.14E-05 | 0.003679 | 4.893014 |
| iqub | 28.44852 | 8.07E-05 | 0.003679 | 4.830282 |
| LOC112486456 | 28.24373 | 8.02E-05 | 0.003679 | 4.819859 |
| LOC103392862 | 24.39321 | 8.22E-05 | 0.003679 | 4.608408 |
| plk3 | 0.04687 | 8.22E-05 | 0.003679 | 4.415184 |
| ccdc89 | 19.57437 | 8.00E-05 | 0.003679 | 4.290894 |
| katnal2 | 19.185 | 8.20E-05 | 0.003679 | 4.261907 |
| wdr66 | 9.898545 | 8.16E-05 | 0.003679 | 3.307216 |
| LOC103379866 | 0.149398 | 8.11E-05 | 0.003679 | 2.742765 |
| LOC103396428 | 39.97506 | 8.38E-05 | 0.003698 | 5.321028 |
| LOC103382184 | 26.019 | 8.33E-05 | 0.003698 | 4.701494 |
| LOC103395088 | 16.90078 | 8.38E-05 | 0.003698 | 4.079018 |
| LOC103395826 | 0.096515 | 8.38E-05 | 0.003698 | 3.37311 |
| gadd45b | 9.690809 | 8.38E-05 | 0.003698 | 3.276617 |
| c11h12orf10 | 0.218833 | 8.48E-05 | 0.003731 | 2.192096 |
| LOC103386283 | 141.1594 | 8.56E-05 | 0.003755 | 7.141182 |
| ropn1l | 45.90473 | 8.81E-05 | 0.003755 | 5.520571 |
| LOC103384229 | 37.02414 | 8.63E-05 | 0.003755 | 5.210394 |
| LOC103390592 | 23.05779 | 8.76E-05 | 0.003755 | 4.527182 |
| LOC103390820 | 19.6551 | 8.72E-05 | 0.003755 | 4.296832 |
| jhy | 13.64086 | 8.70E-05 | 0.003755 | 3.769862 |
| LOC103392554 | 11.70826 | 8.60E-05 | 0.003755 | 3.549455 |
| LOC103386321 | 0.085806 | 8.69E-05 | 0.003755 | 3.542784 |
| LOC103377501 | 11.30312 | 8.68E-05 | 0.003755 | 3.498649 |
| parvb | 0.091274 | 8.82E-05 | 0.003755 | 3.453651 |
| LOC103380984 | 6.923949 | 8.61E-05 | 0.003755 | 2.791595 |
| pet100 | 4.992126 | 8.73E-05 | 0.003755 | 2.319654 |
| LOC112487454 | 4.831006 | 8.69E-05 | 0.003755 | 2.272324 |
| LOC103379822 | 4.63909 | 8.68E-05 | 0.003755 | 2.213842 |
| LOC103391372 | 75.60216 | 8.85E-05 | 0.003755 | 6.240355 |
| LOC112487874 | 0.133261 | 8.91E-05 | 0.003755 | 2.907672 |
| LOC103390519 | 33.79442 | 8.95E-05 | 0.003761 | 5.078713 |
| eif4enif1 | 0.205661 | 8.98E-05 | 0.003766 | 2.281661 |
| klhl36 | 7.574725 | 9.01E-05 | 0.003772 | 2.921193 |
| LOC103385254 | 4.334273 | 9.14E-05 | 0.003795 | 2.11579 |
| barhl2 | 4.126787 | 9.14E-05 | 0.003795 | 2.045019 |
| LOC103388753 | 32.56647 | 9.21E-05 | 0.003797 | 5.025315 |
| LOC103382983 | 32.52268 | 9.20E-05 | 0.003797 | 5.023374 |
| LOC103382335 | 0.165185 | 9.25E-05 | 0.003804 | 2.597845 |
| LOC107989479 | 41.40974 | 9.30E-05 | 0.003806 | 5.371898 |
| LOC103395140 | 16.68662 | 9.31E-05 | 0.003806 | 4.06062 |
| LOC103397910 | 5.830557 | 9.32E-05 | 0.003806 | 2.543634 |
| LOC103382241 | 0.155167 | 9.40E-05 | 0.00382 | 2.688108 |
| nub1 | 5.431341 | 9.42E-05 | 0.003821 | 2.441309 |
| LOC103381772 | 25.47023 | 9.46E-05 | 0.003826 | 4.67074 |
| LOC103382459 | 0.01651 | 9.73E-05 | 0.003879 | 5.920487 |
| LOC103388124 | 11.99485 | 9.73E-05 | 0.003879 | 3.584344 |
| spire2 | 11.43871 | 9.69E-05 | 0.003879 | 3.515852 |
| LOC103392664 | 0.092296 | 9.73E-05 | 0.003879 | 3.43758 |
| LOC103383486 | 4.698639 | 9.70E-05 | 0.003879 | 2.232243 |
| LOC103397915 | 0.174597 | 9.88E-05 | 0.003917 | 2.517899 |
| LOC112487522 | 33.84077 | 9.94E-05 | 0.003927 | 5.080691 |
| tmem132e | 0.214698 | 9.94E-05 | 0.003927 | 2.219618 |
| LOC103394953 | 0.127853 | 9.99E-05 | 0.003935 | 2.967443 |
| LOC103382212 | 35.7718 | 0.000102 | 0.003982 | 5.160751 |
| ipo4 | 7.124054 | 0.000102 | 0.003982 | 2.832698 |
| LOC103390510 | 47.81836 | 0.000102 | 0.003987 | 5.579493 |
| LOC103387743 | 5.107628 | 0.000102 | 0.003988 | 2.352654 |
| LOC103392569 | 26.89472 | 0.000104 | 0.004031 | 4.749251 |
| usp43 | 0.190538 | 0.000104 | 0.004031 | 2.391853 |
| LOC112488256 | 56.11699 | 0.000105 | 0.004037 | 5.810366 |
| LOC103387381 | 29.04714 | 0.000106 | 0.004037 | 4.860324 |
| LOC103388305 | 0.131391 | 0.000105 | 0.004037 | 2.92806 |
| LOC112487717 | 6.171377 | 0.000105 | 0.004037 | 2.625592 |
| LOC103382907 | 4.811181 | 0.000105 | 0.004037 | 2.266391 |
| emx2 | 0.116116 | 0.000106 | 0.004044 | 3.106358 |
| LOC103382478 | 23.15746 | 0.000107 | 0.004053 | 4.533405 |
| LOC112487827 | 14.43465 | 0.000107 | 0.004053 | 3.851464 |
| ldlrad2 | 8.408435 | 0.000107 | 0.004053 | 3.071837 |
| LOC103381032 | 4.686072 | 0.000107 | 0.004053 | 2.228379 |
| LOC103395122 | 0.068161 | 0.000108 | 0.004077 | 3.874907 |
| LOC103397674 | 0.037861 | 0.000109 | 0.00409 | 4.723129 |
| LOC103384618 | 5.385286 | 0.00011 | 0.004094 | 2.429023 |
| LOC103390540 | 0.211225 | 0.00011 | 0.004094 | 2.243148 |
| LOC103384707 | 4.524926 | 0.000109 | 0.004094 | 2.177894 |
| chpt1 | 0.214911 | 0.00011 | 0.004112 | 2.218189 |
| LOC103377500 | 93.28607 | 0.000111 | 0.004127 | 6.54359 |
| LOC103397732 | 0.154688 | 0.000112 | 0.004127 | 2.692564 |
| LOC103396872 | 0.126583 | 0.000112 | 0.00413 | 2.981845 |
| LOC103381721 | 60.25637 | 0.000113 | 0.004144 | 5.913042 |
| frrs1l | 9.37471 | 0.000113 | 0.004144 | 3.228774 |
| stmnd1 | 21.24049 | 0.000114 | 0.004169 | 4.408745 |
| LOC103381722 | 8.938527 | 0.000114 | 0.004169 | 3.160037 |
| LOC103384891 | 4.310315 | 0.000114 | 0.004169 | 2.107793 |
| cdrt1 | 35.58126 | 0.000117 | 0.004234 | 5.153046 |
| LOC103383327 | 4.993813 | 0.000118 | 0.004244 | 2.320142 |
| tpo | 30.41845 | 0.000119 | 0.004246 | 4.926875 |
| cfap300 | 30.26154 | 0.000119 | 0.004246 | 4.919414 |
| LOC103380619 | 0.082749 | 0.000119 | 0.004246 | 3.595109 |
| LOC103385830 | 9.755484 | 0.000118 | 0.004246 | 3.286213 |
| LOC103399836 | 0.108002 | 0.000119 | 0.004246 | 3.210868 |
| elob | 4.466222 | 0.000119 | 0.004246 | 2.159055 |
| tent5c | 0.050001 | 0.00012 | 0.004268 | 4.321894 |
| mid1ip1 | 8.322358 | 0.00012 | 0.004268 | 3.056992 |
| itm2c | 7.313813 | 0.000121 | 0.004272 | 2.870624 |
| LOC103393554 | 19.84147 | 0.000121 | 0.004276 | 4.310447 |
| nop58 | 0.19861 | 0.000122 | 0.004278 | 2.331989 |
| cblb | 0.151955 | 0.000122 | 0.004279 | 2.718287 |
| LOC103394643 | 44.02996 | 0.000123 | 0.004287 | 5.460414 |
| LOC103393397 | 0.083995 | 0.000123 | 0.004296 | 3.573556 |
| LOC103389406 | 0.087751 | 0.000124 | 0.004304 | 3.510443 |
| LOC103399842 | 0.113735 | 0.000124 | 0.004304 | 3.136253 |
| LOC107990035 | 16.40836 | 0.000125 | 0.004304 | 4.036359 |
| rrh | 0.09193 | 0.000124 | 0.004304 | 3.443324 |
| LOC103383290 | 5.911196 | 0.000125 | 0.004304 | 2.56345 |
| LOC103391662 | 0.190207 | 0.000124 | 0.004304 | 2.394357 |
| apbb3 | 30.33468 | 0.000126 | 0.004316 | 4.922896 |
| LOC103399250 | 22.50629 | 0.000126 | 0.004316 | 4.492257 |
| LOC103393713 | 5.944331 | 0.000125 | 0.004316 | 2.571515 |
| glul | 0.225858 | 0.000126 | 0.004316 | 2.146515 |
| LOC103391426 | 118.6117 | 0.000129 | 0.004321 | 6.890103 |
| LOC103379551 | 0.010895 | 0.00013 | 0.004321 | 6.520227 |
| LOC103387785 | 37.15718 | 0.000127 | 0.004321 | 5.215569 |
| LOC103390661 | 34.39804 | 0.000127 | 0.004321 | 5.104255 |
| LOC103394094 | 15.53623 | 0.000129 | 0.004321 | 3.957564 |
| LOC103391689 | 0.084551 | 0.00013 | 0.004321 | 3.564035 |
| LOC112488486 | 9.008731 | 0.00013 | 0.004321 | 3.171324 |
| rad23b | 6.17927 | 0.000128 | 0.004321 | 2.627436 |
| gfra1 | 5.854823 | 0.000127 | 0.004321 | 2.549625 |
| bcas3 | 5.617392 | 0.00013 | 0.004321 | 2.4899 |
| ubfd1 | 4.001198 | 0.000129 | 0.004321 | 2.000432 |
| LOC103385043 | 0.046398 | 0.000132 | 0.004346 | 4.42979 |
| LOC103395090 | 16.54084 | 0.000132 | 0.004346 | 4.04796 |
| LOC103391157 | 0.191373 | 0.000132 | 0.004346 | 2.385539 |
| LOC103381225 | 5.205934 | 0.000132 | 0.004346 | 2.380157 |
| LOC103376895 | 0.197097 | 0.000132 | 0.004346 | 2.343022 |
| LOC112487650 | 0.215114 | 0.000132 | 0.004346 | 2.216824 |
| fgf5 | 21.76531 | 0.000134 | 0.004373 | 4.443959 |
| LOC103394206 | 0.158867 | 0.000135 | 0.004409 | 2.654108 |
| lmod1 | 14.55134 | 0.000136 | 0.004428 | 3.86308 |
| LOC103387242 | 19.94304 | 0.000137 | 0.004433 | 4.317814 |
| LOC103379260 | 21.17284 | 0.000138 | 0.004449 | 4.404143 |
| LOC103396798 | 0.022964 | 0.000138 | 0.004452 | 5.444474 |
| LOC103392382 | 67.76511 | 0.00014 | 0.004457 | 6.082471 |
| LOC103390244 | 0.0203 | 0.00014 | 0.004457 | 5.622401 |
| kiaa1257 | 27.35276 | 0.000139 | 0.004457 | 4.773615 |
| LOC103380736 | 7.502126 | 0.00014 | 0.004457 | 2.907299 |
| baz1a | 4.154265 | 0.00014 | 0.004457 | 2.054593 |
| LOC107988082 | 15.95296 | 0.000141 | 0.004466 | 3.995752 |
| LOC112488518 | 6.052392 | 0.000141 | 0.004466 | 2.597505 |
| LOC103399961 | 0.220812 | 0.000141 | 0.004466 | 2.179113 |
| LOC103390204 | 7.510143 | 0.000142 | 0.004495 | 2.90884 |
| map7d2 | 35.13296 | 0.000143 | 0.004514 | 5.134753 |
| vrtn | 0.081335 | 0.000143 | 0.004514 | 3.619987 |
| crat | 4.822187 | 0.000143 | 0.004514 | 2.269688 |
| LOC103396219 | 24.23602 | 0.000144 | 0.004523 | 4.599081 |
| mmp17 | 0.187093 | 0.000145 | 0.004538 | 2.418173 |
| LOC103396851 | 25.21105 | 0.000147 | 0.004562 | 4.655984 |
| LOC103394181 | 23.41387 | 0.000147 | 0.004562 | 4.549292 |
| pdyn | 18.79452 | 0.000147 | 0.004562 | 4.23224 |
| LOC103396997 | 0.077711 | 0.000147 | 0.004562 | 3.685741 |
| LOC103383961 | 8.119374 | 0.000146 | 0.004562 | 3.021369 |
| nqo1 | 7.731991 | 0.000147 | 0.004562 | 2.95084 |
| efl1 | 5.676739 | 0.000147 | 0.004562 | 2.505062 |
| LOC103378815 | 4.629845 | 0.000148 | 0.004568 | 2.210964 |
| LOC107990253 | 30.32816 | 0.00015 | 0.004604 | 4.922586 |
| kiaa1211 | 0.062977 | 0.000149 | 0.004604 | 3.989025 |
| ppme1 | 4.052799 | 0.00015 | 0.004607 | 2.018919 |
| LOC103396584 | 0.099106 | 0.00015 | 0.004608 | 3.334889 |
| LOC103377360 | 62.58605 | 0.000152 | 0.00465 | 5.967769 |
| LOC103386765 | 30.85318 | 0.000153 | 0.004666 | 4.947347 |
| vgll2 | 8.011457 | 0.000154 | 0.004666 | 3.002065 |
| LOC103388635 | 35.20259 | 0.000154 | 0.004667 | 5.13761 |
| LOC103397052 | 0.071995 | 0.000154 | 0.004667 | 3.795964 |
| LOC112487046 | 12.45388 | 0.000155 | 0.004672 | 3.638523 |
| patl2 | 0.089578 | 0.000156 | 0.004681 | 3.480719 |
| clcc1 | 0.09817 | 0.000156 | 0.004681 | 3.348568 |
| kif1a | 0.041409 | 0.000156 | 0.004686 | 4.593898 |
| sdc3 | 0.088769 | 0.000157 | 0.004688 | 3.493808 |
| LOC103387397 | 21.16255 | 0.000159 | 0.004728 | 4.403442 |
| LOC103397704 | 0.130071 | 0.000159 | 0.004728 | 2.942625 |
| LOC103388010 | 0.165505 | 0.00016 | 0.004745 | 2.595052 |
| LOC103380362 | 29.41933 | 0.000162 | 0.00477 | 4.878692 |
| si | 8.17365 | 0.000162 | 0.00477 | 3.030981 |
| tcirg1 | 0.16341 | 0.000162 | 0.00477 | 2.613436 |
| LOC112487889 | 0.111729 | 0.000163 | 0.004784 | 3.161924 |
| mtr | 0.164101 | 0.000164 | 0.004795 | 2.607341 |
| cfap69 | 22.73885 | 0.000165 | 0.004824 | 4.507088 |
| erg28 | 0.119163 | 0.000166 | 0.00483 | 3.068994 |
| LOC103378375 | 4.782591 | 0.000167 | 0.004841 | 2.257792 |
| LOC103388319 | 0.13527 | 0.000167 | 0.004845 | 2.886084 |
| LOC112488406 | 49.34666 | 0.000167 | 0.004855 | 5.624881 |
| LOC103384280 | 0.083283 | 0.000168 | 0.004855 | 3.585826 |
| slc24a3 | 6.897603 | 0.000169 | 0.004857 | 2.786095 |
| eef1akmt3 | 0.148248 | 0.000168 | 0.004857 | 2.753916 |
| LOC103383893 | 0.029375 | 0.000169 | 0.004859 | 5.08927 |
| LOC103395956 | 24.69643 | 0.00017 | 0.004872 | 4.626231 |
| LOC103384242 | 9.516706 | 0.000171 | 0.004896 | 3.250462 |
| LOC103384248 | 30.36301 | 0.000171 | 0.004897 | 4.924243 |
| st3gal4 | 7.190784 | 0.000171 | 0.004898 | 2.846149 |
| LOC103376858 | 22.55896 | 0.000173 | 0.004905 | 4.495628 |
| LOC103394607 | 0.097574 | 0.000172 | 0.004905 | 3.357357 |
| LOC103392203 | 7.100915 | 0.000172 | 0.004905 | 2.828005 |
| LOC107989474 | 6.785617 | 0.000172 | 0.004905 | 2.76248 |
| LOC107988069 | 28.77488 | 0.000174 | 0.004924 | 4.846738 |
| oser1 | 4.184207 | 0.000174 | 0.004924 | 2.064954 |
| LOC107989490 | 8.333744 | 0.000175 | 0.004943 | 3.058965 |
| LOC103384469 | 17.97319 | 0.000176 | 0.004959 | 4.167774 |
| serpine2 | 0.200112 | 0.000177 | 0.004974 | 2.321118 |
| ufd1 | 6.178478 | 0.000178 | 0.00498 | 2.627251 |
| bmp15 | 0.139546 | 0.000178 | 0.004985 | 2.841191 |
| evx1 | 0.159349 | 0.000179 | 0.004985 | 2.649735 |
| usp4 | 4.813786 | 0.000179 | 0.004985 | 2.267172 |
| LOC103389835 | 18.35584 | 0.000181 | 0.005009 | 4.198167 |
| LOC103392434 | 0.246864 | 0.000181 | 0.005009 | 2.018209 |
| LOC103395149 | 20.35792 | 0.000181 | 0.005015 | 4.347518 |
| LOC103395869 | 20.1827 | 0.000183 | 0.00502 | 4.335047 |
| LOC103385446 | 0.053746 | 0.000183 | 0.00502 | 4.2177 |
| LOC103377701 | 14.5663 | 0.000184 | 0.00502 | 3.864562 |
| LOC103389263 | 13.38926 | 0.000184 | 0.00502 | 3.743005 |
| LOC103389985 | 0.080721 | 0.000184 | 0.00502 | 3.630912 |
| LOC107988371 | 8.036107 | 0.000182 | 0.00502 | 3.006497 |
| lsm11 | 0.187473 | 0.000184 | 0.00502 | 2.415248 |
| LOC103392639 | 18.60574 | 0.000186 | 0.005031 | 4.217676 |
| LOC103397794 | 5.549429 | 0.000185 | 0.005031 | 2.472339 |
| hoxc12 | 0.144039 | 0.000186 | 0.005034 | 2.795466 |
| plcz1 | 26.68385 | 0.000188 | 0.005047 | 4.737895 |
| LOC103386601 | 18.43309 | 0.000188 | 0.005047 | 4.204226 |
| LOC103391622 | 0.221575 | 0.000188 | 0.005047 | 2.174134 |
| acyp1 | 5.907442 | 0.000188 | 0.005049 | 2.562534 |
| LOC103385211 | 43.45803 | 0.00019 | 0.005082 | 5.441551 |
| fam187a | 12.38653 | 0.00019 | 0.005082 | 3.6307 |
| LOC103389581 | 24.51006 | 0.00019 | 0.005088 | 4.615302 |
| stox1 | 0.212164 | 0.00019 | 0.005088 | 2.23675 |
| LOC103393637 | 17.58007 | 0.000191 | 0.005088 | 4.135869 |
| ctsa | 0.134037 | 0.000192 | 0.005115 | 2.899297 |
| LOC112488602 | 17.25983 | 0.000195 | 0.00515 | 4.109347 |
| fst | 0.160555 | 0.000196 | 0.005152 | 2.63886 |
| LOC103392079 | 0.153128 | 0.000196 | 0.005154 | 2.707188 |
| LOC103381895 | 46.36528 | 0.000196 | 0.00516 | 5.534973 |
| LOC103390132 | 14.45035 | 0.000199 | 0.005195 | 3.853033 |
| LOC103387667 | 0.13632 | 0.000198 | 0.005195 | 2.874928 |
| LOC103388745 | 24.65049 | 0.000199 | 0.0052 | 4.623544 |
| tp73 | 10.53651 | 0.0002 | 0.0052 | 3.397325 |
| LOC103383808 | 0.192404 | 0.0002 | 0.0052 | 2.37779 |
| LOC103391020 | 4.306655 | 0.000199 | 0.0052 | 2.106568 |
| LOC103393550 | 0.113458 | 0.0002 | 0.005203 | 3.139773 |
| LOC107989498 | 24.02236 | 0.000201 | 0.005203 | 4.586306 |
| LOC103395889 | 8.488121 | 0.000201 | 0.005203 | 3.085445 |
| LOC103383574 | 0.238135 | 0.000201 | 0.005203 | 2.070149 |
| LOC103397574 | 0.239646 | 0.000204 | 0.005235 | 2.061021 |
| LOC107990159 | 8.401627 | 0.000205 | 0.00524 | 3.070669 |
| osbpl7 | 0.159239 | 0.000205 | 0.005243 | 2.650732 |
| LOC103377514 | 0.013369 | 0.000206 | 0.005246 | 6.224968 |
| LOC103383697 | 11.27753 | 0.000206 | 0.005246 | 3.495379 |
| LOC112487640 | 4.012795 | 0.000206 | 0.005249 | 2.004607 |
| LOC103377699 | 13.58889 | 0.000208 | 0.005275 | 3.764356 |
| fras1 | 0.192043 | 0.000208 | 0.005275 | 2.380496 |
| kiaa0408 | 0.22331 | 0.000208 | 0.005275 | 2.162878 |
| LOC103379418 | 11.95788 | 0.000209 | 0.005275 | 3.57989 |
| npas3 | 14.0887 | 0.000211 | 0.005278 | 3.816466 |
| agbl5 | 13.89429 | 0.00021 | 0.005278 | 3.79642 |
| slco2a1 | 0.098198 | 0.000211 | 0.005278 | 3.348158 |
| LOC103381373 | 9.851109 | 0.00021 | 0.005278 | 3.300286 |
| LOC103379036 | 9.820959 | 0.00021 | 0.005278 | 3.295864 |
| LOC103389744 | 0.17222 | 0.00021 | 0.005278 | 2.537673 |
| cnpy2 | 0.179909 | 0.000211 | 0.005278 | 2.474659 |
| isca1 | 4.435618 | 0.00021 | 0.005278 | 2.149135 |
| coil | 0.242044 | 0.000211 | 0.005278 | 2.046661 |
| LOC103385823 | 0.023946 | 0.000212 | 0.005282 | 5.3841 |
| adsl | 0.190841 | 0.000212 | 0.005282 | 2.389553 |
| LOC103390386 | 0.012817 | 0.000214 | 0.005304 | 6.285847 |
| fbxo43 | 0.097097 | 0.000214 | 0.005307 | 3.364428 |
| LOC103387724 | 19.83372 | 0.000215 | 0.005322 | 4.309883 |
| LOC103379682 | 37.21974 | 0.000216 | 0.005349 | 5.217996 |
| sobp | 0.238221 | 0.000216 | 0.005349 | 2.069628 |
| LOC103397550 | 15.50952 | 0.000217 | 0.00535 | 3.955082 |
| LOC112487928 | 18.411 | 0.000217 | 0.005362 | 4.202496 |
| nin | 0.249497 | 0.000218 | 0.005362 | 2.002905 |
| gdf9 | 0.051064 | 0.000221 | 0.00539 | 4.291559 |
| ttc9c | 5.090535 | 0.00022 | 0.00539 | 2.347817 |
| zmynd10 | 8.573892 | 0.000221 | 0.005402 | 3.09995 |
| LOC103391244 | 0.134046 | 0.000222 | 0.005404 | 2.899204 |
| fbxw9 | 5.592723 | 0.000222 | 0.005404 | 2.483551 |
| LOC103394758 | 0.155222 | 0.000222 | 0.005407 | 2.6876 |
| LOC103389570 | 0.072261 | 0.000223 | 0.005412 | 3.790648 |
| LOC103377596 | 0.195881 | 0.000223 | 0.005412 | 2.351949 |
| LOC103390721 | 13.13049 | 0.000223 | 0.005414 | 3.714849 |
| sh3glb2 | 0.099903 | 0.000224 | 0.005414 | 3.323335 |
| synpo | 0.22548 | 0.000225 | 0.005431 | 2.148926 |
| LOC103392979 | 12.075 | 0.000225 | 0.005436 | 3.593951 |
| smtnl2 | 4.226534 | 0.000226 | 0.005437 | 2.079475 |
| LOC107989881 | 20.87422 | 0.000227 | 0.005451 | 4.38365 |
| LOC103379675 | 10.27476 | 0.000228 | 0.00546 | 3.361033 |
| LOC107988365 | 45.41745 | 0.000231 | 0.005474 | 5.505175 |
| LOC103390507 | 26.16048 | 0.00023 | 0.005474 | 4.709317 |
| LOC103384378 | 0.096859 | 0.000232 | 0.005474 | 3.367974 |
| c14h8orf74 | 5.662145 | 0.000232 | 0.005474 | 2.501349 |
| dnmt1 | 0.176824 | 0.000229 | 0.005474 | 2.49961 |
| LOC103384726 | 0.187944 | 0.00023 | 0.005474 | 2.411627 |
| mindy3 | 4.397122 | 0.000232 | 0.005474 | 2.13656 |
| nsun6 | 4.340326 | 0.000231 | 0.005474 | 2.117804 |
| LOC103384184 | 5.555234 | 0.000232 | 0.005478 | 2.473848 |
| LOC103384193 | 0.15197 | 0.000233 | 0.005486 | 2.71814 |
| qtrt1 | 0.22613 | 0.000236 | 0.005524 | 2.144775 |
| LOC103386493 | 36.79405 | 0.00024 | 0.005526 | 5.201401 |
| LOC107989471 | 20.91101 | 0.000239 | 0.005526 | 4.386191 |
| LOC103394237 | 0.08285 | 0.00024 | 0.005526 | 3.593357 |
| mdm1 | 6.693808 | 0.000239 | 0.005526 | 2.742827 |
| zfp36l2 | 0.167771 | 0.00024 | 0.005526 | 2.575437 |
| nup54 | 0.178877 | 0.000237 | 0.005526 | 2.48296 |
| LOC103389693 | 4.814719 | 0.000237 | 0.005526 | 2.267451 |
| LOC103379359 | 0.228616 | 0.000239 | 0.005526 | 2.128999 |
| dtwd2 | 0.239485 | 0.000238 | 0.005526 | 2.061992 |
| LOC103391990 | 0.105744 | 0.000241 | 0.005527 | 3.241359 |
| LOC103379920 | 0.132686 | 0.000241 | 0.005529 | 2.913916 |
| LOC103388931 | 6.316943 | 0.000242 | 0.00553 | 2.659227 |
| anks4b | 6.195523 | 0.000242 | 0.00553 | 2.631226 |
| LOC103393547 | 17.88413 | 0.000244 | 0.005533 | 4.160608 |
| dzank1 | 17.42999 | 0.000245 | 0.005533 | 4.1235 |
| LOC103391654 | 0.161632 | 0.000243 | 0.005533 | 2.629214 |
| ccdc25 | 5.940869 | 0.000245 | 0.005533 | 2.570674 |
| slc35e4 | 5.902592 | 0.000245 | 0.005533 | 2.561349 |
| nt5c3a | 5.121894 | 0.000244 | 0.005533 | 2.356677 |
| LOC103395673 | 52.78582 | 0.000245 | 0.005533 | 5.722079 |
| LOC103390502 | 145.2277 | 0.000246 | 0.005557 | 7.182173 |
| LOC107990061 | 48.14258 | 0.000248 | 0.00556 | 5.589242 |
| LOC103385229 | 17.54015 | 0.000249 | 0.00556 | 4.132589 |
| otol1 | 0.096613 | 0.000249 | 0.00556 | 3.371637 |
| manf | 5.960987 | 0.00025 | 0.00556 | 2.575551 |
| LOC103386870 | 0.188225 | 0.00025 | 0.00556 | 2.409473 |
| LOC103397007 | 0.204176 | 0.000248 | 0.00556 | 2.292115 |
| irx3 | 0.207293 | 0.00025 | 0.00556 | 2.270256 |
| rnf31 | 0.22672 | 0.000248 | 0.00556 | 2.14102 |
| LOC103392030 | 0.067075 | 0.000251 | 0.005574 | 3.898081 |
| LOC103384456 | 0.095169 | 0.000251 | 0.005574 | 3.393361 |
| LOC103382960 | 0.136198 | 0.000252 | 0.005577 | 2.876225 |
| LOC103393784 | 0.087406 | 0.000252 | 0.005578 | 3.516122 |
| LOC103389436 | 0.22301 | 0.000253 | 0.005594 | 2.164819 |
| LOC112487899 | 0.206216 | 0.000253 | 0.005599 | 2.277773 |
| LOC103387951 | 21.26496 | 0.000254 | 0.005602 | 4.410406 |
| LOC103386655 | 20.01894 | 0.000254 | 0.005602 | 4.323294 |
| vash1 | 11.42201 | 0.000254 | 0.005602 | 3.513745 |
| trnal-aag | 5.914009 | 0.000254 | 0.005602 | 2.564136 |
| ice1 | 6.154671 | 0.000255 | 0.005604 | 2.621682 |
| LOC103377557 | 25.96981 | 0.000258 | 0.005642 | 4.698764 |
| LOC103396062 | 0.069643 | 0.000258 | 0.005646 | 3.843888 |
| LOC103382331 | 0.173527 | 0.000259 | 0.005662 | 2.526764 |
| hydin | 12.23184 | 0.000261 | 0.005679 | 3.612569 |
| LOC103386027 | 8.405706 | 0.000261 | 0.005679 | 3.071369 |
| morn2 | 10.07856 | 0.000262 | 0.005698 | 3.333217 |
| LOC103381019 | 0.151241 | 0.000263 | 0.005718 | 2.725081 |
| LOC112488358 | 5.936075 | 0.000267 | 0.005785 | 2.569509 |
| LOC103385461 | 0.149814 | 0.000268 | 0.005795 | 2.738755 |
| rcn2 | 15.87282 | 0.000271 | 0.005844 | 3.988487 |
| arid3a | 5.83694 | 0.000271 | 0.005844 | 2.545212 |
| LOC103380140 | 4.805715 | 0.000272 | 0.005858 | 2.264751 |
| inha | 0.223875 | 0.000273 | 0.005858 | 2.159234 |
| LOC103390836 | 11.84784 | 0.000273 | 0.005869 | 3.566553 |
| dcst2 | 16.74773 | 0.000277 | 0.00592 | 4.065893 |
| LOC103376867 | 72.05841 | 0.000278 | 0.005926 | 6.171095 |
| mgll | 8.179328 | 0.00028 | 0.005926 | 3.031982 |
| igdcc3 | 0.152564 | 0.00028 | 0.005926 | 2.712516 |
| chac2 | 4.999731 | 0.000281 | 0.005926 | 2.321851 |
| LOC103386363 | 0.217497 | 0.00028 | 0.005926 | 2.200932 |
| LOC103397996 | 9.285073 | 0.000282 | 0.005941 | 3.214913 |
| LOC103394710 | 31.89576 | 0.000285 | 0.005947 | 4.995293 |
| LOC103399864 | 21.0293 | 0.000285 | 0.005947 | 4.394329 |
| LOC103380095 | 5.391253 | 0.000286 | 0.005947 | 2.430621 |
| c11h20orf85 | 5.382607 | 0.000284 | 0.005947 | 2.428305 |
| LOC103378748 | 0.21905 | 0.000286 | 0.005947 | 2.190668 |
| kcnk12 | 0.230678 | 0.000283 | 0.005947 | 2.11605 |
| LOC112488625 | 4.275559 | 0.000285 | 0.005947 | 2.096113 |
| csnk2a2 | 0.238164 | 0.000284 | 0.005947 | 2.069971 |
| LOC103398303 | 0.240355 | 0.000283 | 0.005947 | 2.056764 |
| LOC103393541 | 14.38679 | 0.000287 | 0.005969 | 3.846673 |
| cfap298 | 5.385776 | 0.000289 | 0.005984 | 2.429154 |
| LOC103388007 | 0.242989 | 0.000289 | 0.005984 | 2.041036 |
| LOC103383221 | 5.873266 | 0.00029 | 0.005989 | 2.554163 |
| LOC103388006 | 4.294433 | 0.00029 | 0.005994 | 2.102468 |
| LOC103383436 | 25.2029 | 0.000291 | 0.005999 | 4.655518 |
| LOC103393357 | 66.74994 | 0.000291 | 0.006003 | 6.060695 |
| LOC103383625 | 0.204089 | 0.000292 | 0.006003 | 2.292728 |
| LOC103399895 | 50.11869 | 0.000297 | 0.006055 | 5.647277 |
| LOC103378789 | 19.50671 | 0.000298 | 0.006055 | 4.285899 |
| LOC103376831 | 16.39017 | 0.000297 | 0.006055 | 4.034759 |
| LOC103382007 | 8.384828 | 0.000296 | 0.006055 | 3.067781 |
| vegfd | 0.121759 | 0.000297 | 0.006055 | 3.037902 |
| LOC103382140 | 5.259063 | 0.000296 | 0.006055 | 2.394806 |
| dnajb6 | 4.814375 | 0.000296 | 0.006055 | 2.267349 |
| LOC112488305 | 15.3074 | 0.000301 | 0.006063 | 3.936157 |
| LOC103389417 | 7.296276 | 0.0003 | 0.006063 | 2.86716 |
| pim3 | 5.665827 | 0.000301 | 0.006063 | 2.502287 |
| mtch2 | 5.916339 | 0.000301 | 0.00607 | 2.564705 |
| LOC103392972 | 21.22252 | 0.000304 | 0.006092 | 4.407524 |
| tram1 | 0.183397 | 0.000303 | 0.006092 | 2.446955 |
| LOC112488150 | 7.458518 | 0.000304 | 0.006094 | 2.898889 |
| LOC103393850 | 4.618557 | 0.000306 | 0.006113 | 2.207442 |
| LOC103384272 | 27.34948 | 0.000309 | 0.006142 | 4.773441 |
| ccr6 | 8.203977 | 0.000309 | 0.006142 | 3.036323 |
| LOC103383004 | 21.79906 | 0.000309 | 0.006147 | 4.446194 |
| LOC103390712 | 10.11122 | 0.000309 | 0.006147 | 3.337885 |
| LOC103390508 | 21.13 | 0.000313 | 0.006208 | 4.401221 |
| ubxn11 | 22.45693 | 0.000314 | 0.006209 | 4.489089 |
| LOC103380025 | 0.154103 | 0.000315 | 0.00623 | 2.698037 |
| LOC103378354 | 29.14535 | 0.000318 | 0.006233 | 4.865194 |
| LOC103381495 | 0.046331 | 0.000315 | 0.006233 | 4.43188 |
| farsa | 0.08529 | 0.000318 | 0.006233 | 3.551487 |
| ttll10 | 0.112836 | 0.000316 | 0.006233 | 3.1477 |
| LOC103394984 | 0.115725 | 0.000318 | 0.006233 | 3.111232 |
| sgsm2 | 0.120114 | 0.000316 | 0.006233 | 3.057526 |
| LOC103379644 | 0.123984 | 0.000318 | 0.006233 | 3.011775 |
| tent5b | 0.242004 | 0.000317 | 0.006233 | 2.046895 |
| LOC103376852 | 11.49197 | 0.000319 | 0.006238 | 3.522555 |
| LOC112487741 | 9.667989 | 0.00032 | 0.006238 | 3.273216 |
| slc6a20 | 0.110163 | 0.00032 | 0.006238 | 3.182287 |
| syde1 | 0.210301 | 0.00032 | 0.006238 | 2.249474 |
| LOC103379726 | 4.134209 | 0.000319 | 0.006238 | 2.047611 |
| rsph10b | 11.24416 | 0.000323 | 0.00624 | 3.491104 |
| LOC103393669 | 9.313467 | 0.000323 | 0.00624 | 3.219318 |
| LOC103384957 | 0.147677 | 0.000323 | 0.00624 | 2.759481 |
| LOC103377591 | 0.239732 | 0.000323 | 0.00624 | 2.060507 |
| LOC107988601 | 25.42154 | 0.000327 | 0.006271 | 4.66798 |
| LOC103380154 | 0.144712 | 0.000327 | 0.006271 | 2.788746 |
| trnam-cau | 5.002622 | 0.000328 | 0.006277 | 2.322684 |
| LOC103389925 | 0.114027 | 0.000329 | 0.00629 | 3.132558 |
| LOC103389655 | 17.78031 | 0.000331 | 0.006299 | 4.152208 |
| igsf8 | 0.097432 | 0.000332 | 0.006305 | 3.359455 |
| LOC103392636 | 5.675828 | 0.000332 | 0.006305 | 2.504831 |
| ifi30 | 0.071051 | 0.000334 | 0.006327 | 3.814994 |
| LOC103397745 | 4.260864 | 0.000335 | 0.006341 | 2.091146 |
| LOC107989136 | 9.418428 | 0.000336 | 0.006356 | 3.235486 |
| cdkn1b | 0.169601 | 0.000339 | 0.006385 | 2.559782 |
| parp12 | 0.232496 | 0.00034 | 0.006393 | 2.104725 |
| LOC103397827 | 0.242428 | 0.00034 | 0.006393 | 2.044372 |
| bmp6 | 0.134088 | 0.000341 | 0.006399 | 2.898751 |
| LOC103394727 | 0.097452 | 0.000342 | 0.006401 | 3.359169 |
| LOC103390511 | 5.147625 | 0.000342 | 0.006401 | 2.363907 |
| LOC103378127 | 0.18346 | 0.000343 | 0.006417 | 2.446465 |
| acvr1b | 0.231464 | 0.000343 | 0.006417 | 2.111141 |
| plpp2 | 7.153046 | 0.000345 | 0.00644 | 2.838558 |
| LOC103381661 | 6.935152 | 0.000347 | 0.006461 | 2.793927 |
| LOC103381648 | 0.164605 | 0.000347 | 0.006461 | 2.602923 |
| LOC103378790 | 9.802708 | 0.000348 | 0.006468 | 3.29318 |
| LOC103386032 | 4.686684 | 0.000348 | 0.006468 | 2.228567 |
| rab3b | 4.123935 | 0.000348 | 0.006468 | 2.044021 |
| LOC103394251 | 0.177934 | 0.00035 | 0.00649 | 2.490584 |
| LOC103396786 | 5.564687 | 0.000351 | 0.006502 | 2.4763 |
| acp5 | 0.029682 | 0.000354 | 0.006507 | 5.074259 |
| LOC103393108 | 30.72866 | 0.000356 | 0.006507 | 4.941513 |
| bphl | 19.56724 | 0.000355 | 0.006507 | 4.290368 |
| LOC103395675 | 17.24588 | 0.000353 | 0.006507 | 4.10818 |
| LOC103378794 | 13.17217 | 0.000352 | 0.006507 | 3.719421 |
| LOC103394833 | 5.98511 | 0.000356 | 0.006507 | 2.581378 |
| LOC103389709 | 0.168727 | 0.000355 | 0.006507 | 2.56724 |
| pet117 | 0.186491 | 0.000354 | 0.006507 | 2.422822 |
| aup1 | 4.97331 | 0.000355 | 0.006507 | 2.314206 |
| LOC103383437 | 4.655822 | 0.000355 | 0.006507 | 2.219036 |
| phf3 | 4.46757 | 0.000354 | 0.006507 | 2.15949 |
| LOC103388516 | 0.234543 | 0.000353 | 0.006507 | 2.092076 |
| LOC103396366 | 10.39685 | 0.000357 | 0.006517 | 3.378075 |
| LOC103382319 | 7.244129 | 0.000357 | 0.006522 | 2.856812 |
| LOC103379798 | 0.200319 | 0.00036 | 0.006547 | 2.319625 |
| LOC103397081 | 0.183222 | 0.000363 | 0.00657 | 2.448339 |
| LOC103386879 | 7.530567 | 0.000365 | 0.006592 | 2.912758 |
| izumo1 | 18.38567 | 0.000366 | 0.006597 | 4.20051 |
| LOC103394580 | 42.21294 | 0.000366 | 0.006608 | 5.399613 |
| LOC103377898 | 11.88489 | 0.000368 | 0.006624 | 3.571057 |
| LOC103384034 | 14.37504 | 0.00037 | 0.006659 | 3.845494 |
| LOC103394857 | 57.67926 | 0.000376 | 0.006686 | 5.849981 |
| LOC103380483 | 13.71583 | 0.000375 | 0.006686 | 3.77777 |
| LOC103379606 | 9.457866 | 0.000375 | 0.006686 | 3.241515 |
| LOC103381949 | 0.186241 | 0.000375 | 0.006686 | 2.424754 |
| LOC103389474 | 0.215464 | 0.000374 | 0.006686 | 2.214479 |
| LOC103378246 | 4.361155 | 0.000375 | 0.006686 | 2.12471 |
| faah2 | 0.241971 | 0.000373 | 0.006686 | 2.047094 |
| LOC103385247 | 9.063419 | 0.000378 | 0.006702 | 3.180055 |
| LOC103383506 | 0.135439 | 0.000381 | 0.006728 | 2.88428 |
| relt | 4.458819 | 0.000382 | 0.006741 | 2.156661 |
| cacnb2 | 12.92765 | 0.000384 | 0.006756 | 3.692389 |
| LOC103398066 | 6.79811 | 0.000384 | 0.006756 | 2.765134 |
| insig1 | 0.190453 | 0.000386 | 0.006773 | 2.392493 |
| LOC103382807 | 0.143069 | 0.000387 | 0.006788 | 2.805218 |
| LOC103386902 | 22.30049 | 0.000388 | 0.006791 | 4.479003 |
| map1lc3a | 7.298966 | 0.000389 | 0.006792 | 2.867692 |
| LOC103384036 | 21.90418 | 0.00039 | 0.006799 | 4.453134 |
| LOC103379658 | 11.38326 | 0.00039 | 0.006801 | 3.508842 |
| LOC103397217 | 12.95603 | 0.000392 | 0.006807 | 3.695552 |
| dqx1 | 4.225232 | 0.000391 | 0.006807 | 2.07903 |
| armc3 | 27.92301 | 0.000392 | 0.00681 | 4.803383 |
| tyro3 | 0.235802 | 0.000392 | 0.00681 | 2.084354 |
| sall4 | 0.07902 | 0.000396 | 0.006837 | 3.661642 |
| mcm4 | 0.10113 | 0.000397 | 0.006837 | 3.305722 |
| ppt2 | 7.020618 | 0.000397 | 0.006837 | 2.811598 |
| LOC103388894 | 0.184739 | 0.000395 | 0.006837 | 2.436438 |
| atg101 | 4.181214 | 0.000396 | 0.006837 | 2.063922 |
| LOC103385599 | 12.39457 | 0.0004 | 0.006864 | 3.631637 |
| miga1 | 4.082545 | 0.0004 | 0.006864 | 2.029469 |
| LOC112488012 | 5.750093 | 0.0004 | 0.006865 | 2.523585 |
| c16h2orf40 | 26.66253 | 0.000402 | 0.006877 | 4.736742 |
| wdr93 | 15.25897 | 0.000403 | 0.006883 | 3.931586 |
| dcst1 | 22.31114 | 0.000407 | 0.006921 | 4.479693 |
| LOC103380363 | 5.121307 | 0.000407 | 0.006921 | 2.356512 |
| pomgnt2 | 9.254606 | 0.000409 | 0.006937 | 3.210172 |
| LOC103396953 | 8.439873 | 0.00041 | 0.006937 | 3.077221 |
| LOC103397023 | 7.680554 | 0.000411 | 0.006937 | 2.94121 |
| LOC103378134 | 0.15277 | 0.00041 | 0.006937 | 2.710569 |
| LOC103377856 | 13.0275 | 0.000412 | 0.006941 | 3.703488 |
| LOC103385600 | 10.40709 | 0.000411 | 0.006941 | 3.379495 |
| LOC112487665 | 7.462059 | 0.000412 | 0.006941 | 2.899574 |
| LOC103385199 | 4.140848 | 0.000412 | 0.006941 | 2.049926 |
| LOC103398448 | 5.327916 | 0.000416 | 0.006968 | 2.413571 |
| rmi2 | 0.135951 | 0.000416 | 0.006973 | 2.878841 |
| LOC103385678 | 4.139427 | 0.000416 | 0.006973 | 2.049431 |
| LOC103393536 | 15.80397 | 0.000418 | 0.006983 | 3.982215 |
| LOC103378772 | 0.145621 | 0.000418 | 0.006983 | 2.779708 |
| LOC103378738 | 0.185633 | 0.000418 | 0.006983 | 2.429477 |
| LOC103395758 | 4.100196 | 0.00042 | 0.007002 | 2.035693 |
| adgrb2 | 0.196917 | 0.00042 | 0.007003 | 2.344342 |
| klc2 | 0.238414 | 0.000422 | 0.007021 | 2.068458 |
| LOC103380083 | 0.219894 | 0.000423 | 0.007027 | 2.18512 |
| LOC103392037 | 0.187927 | 0.000424 | 0.007036 | 2.411753 |
| pkhd1l1 | 0.072854 | 0.000427 | 0.007059 | 3.778846 |
| greb1 | 4.975151 | 0.00043 | 0.007088 | 2.31474 |
| aplp1 | 0.24582 | 0.000434 | 0.007142 | 2.024325 |
| trnar-acg | 42.48047 | 0.000437 | 0.007151 | 5.408728 |
| LOC103394568 | 18.90322 | 0.000435 | 0.007151 | 4.24056 |
| LOC103382288 | 0.164041 | 0.000436 | 0.007151 | 2.607869 |
| vangl2 | 0.213491 | 0.000436 | 0.007151 | 2.22775 |
| cfap65 | 8.152719 | 0.000437 | 0.007151 | 3.027281 |
| slc30a3 | 0.152721 | 0.000439 | 0.007155 | 2.711027 |
| rabl3 | 5.551916 | 0.000439 | 0.007155 | 2.472986 |
| LOC103390961 | 0.193478 | 0.000439 | 0.007155 | 2.369757 |
| LOC112486853 | 13.59427 | 0.000441 | 0.007158 | 3.764927 |
| LOC103395116 | 0.073317 | 0.000444 | 0.007197 | 3.769702 |
| cfap70 | 20.81832 | 0.000446 | 0.0072 | 4.379781 |
| LOC103381741 | 10.19738 | 0.000445 | 0.0072 | 3.350127 |
| trnaw-cca | 5.792546 | 0.000447 | 0.007206 | 2.534198 |
| LOC103383547 | 33.34174 | 0.000448 | 0.007207 | 5.059257 |
| LOC103393819 | 9.937826 | 0.000448 | 0.007207 | 3.31293 |
| prkcb | 0.123057 | 0.000448 | 0.007207 | 3.022605 |
| LOC103389678 | 6.942989 | 0.000449 | 0.007209 | 2.795557 |
| fgfr1op | 4.393468 | 0.00045 | 0.007224 | 2.13536 |
| LOC103384043 | 5.528671 | 0.000453 | 0.007247 | 2.466933 |
| LOC103395902 | 0.135589 | 0.000455 | 0.007258 | 2.882689 |
| kdm1b | 5.378279 | 0.000454 | 0.007258 | 2.427145 |
| dcx | 0.247286 | 0.000455 | 0.007258 | 2.015746 |
| LOC103381859 | 8.943259 | 0.000459 | 0.007297 | 3.160801 |
| LOC103377492 | 8.515987 | 0.000458 | 0.007297 | 3.090174 |
| cpeb3 | 4.113909 | 0.00046 | 0.007298 | 2.04051 |
| LOC107989495 | 10.01197 | 0.00046 | 0.007298 | 3.323655 |
| LOC103397616 | 0.071321 | 0.000463 | 0.007324 | 3.80953 |
| LOC103397606 | 0.126033 | 0.000463 | 0.007324 | 2.988126 |
| LOC112487763 | 0.195869 | 0.000464 | 0.007333 | 2.352039 |
| LOC103384322 | 0.136343 | 0.000465 | 0.007337 | 2.874691 |
| LOC103398147 | 7.182057 | 0.000467 | 0.00735 | 2.844397 |
| kif14 | 0.225588 | 0.000468 | 0.007352 | 2.14824 |
| LOC112488619 | 24.58189 | 0.000473 | 0.007366 | 4.619524 |
| LOC103379629 | 6.408383 | 0.000472 | 0.007366 | 2.67996 |
| LOC103394540 | 0.215058 | 0.00047 | 0.007366 | 2.217204 |
| LOC103388391 | 4.284098 | 0.00047 | 0.007366 | 2.098991 |
| LOC103386115 | 0.223651 | 0.000474 | 0.007386 | 2.160681 |
| LOC103383703 | 10.79493 | 0.000476 | 0.007401 | 3.432282 |
| LOC103389810 | 5.002063 | 0.000476 | 0.007401 | 2.322523 |
| LOC103382905 | 15.40137 | 0.000478 | 0.007412 | 3.944987 |
| gabarap | 4.91876 | 0.000479 | 0.007412 | 2.298295 |
| morc2 | 0.239016 | 0.000479 | 0.007412 | 2.064822 |
| LOC103398034 | 5.939277 | 0.00048 | 0.007413 | 2.570287 |
| LOC103394879 | 29.70336 | 0.000483 | 0.007444 | 4.892554 |
| rgs14 | 0.103539 | 0.000485 | 0.007444 | 3.271755 |
| LOC112488400 | 6.485014 | 0.000485 | 0.007444 | 2.69711 |
| cdc6 | 0.182083 | 0.000484 | 0.007444 | 2.457336 |
| LOC103396281 | 0.199318 | 0.000484 | 0.007444 | 2.326857 |
| LOC103388276 | 4.752554 | 0.000485 | 0.007444 | 2.248703 |
| LOC103393369 | 0.230473 | 0.000485 | 0.007444 | 2.11733 |
| LOC103393911 | 0.190857 | 0.000488 | 0.007453 | 2.389433 |
| LOC103385812 | 0.223033 | 0.000487 | 0.007453 | 2.164668 |
| LOC103396746 | 0.229091 | 0.000488 | 0.007453 | 2.12601 |
| LOC103389347 | 4.124279 | 0.000488 | 0.007453 | 2.044142 |
| LOC103393537 | 7.310657 | 0.000489 | 0.007461 | 2.870001 |
| LOC107989243 | 12.79774 | 0.00049 | 0.00747 | 3.677817 |
| ppox | 0.242358 | 0.000491 | 0.00748 | 2.044787 |
| ninl | 0.177732 | 0.000493 | 0.007486 | 2.492226 |
| snap91 | 0.105261 | 0.000495 | 0.007493 | 3.247962 |
| LOC103378277 | 0.071606 | 0.000497 | 0.007497 | 3.803783 |
| LOC103391728 | 0.11935 | 0.000496 | 0.007497 | 3.066726 |
| fmn1 | 8.074719 | 0.000497 | 0.007497 | 3.013412 |
| LOC112488023 | 22.37487 | 0.000497 | 0.007497 | 4.483807 |
| LOC103377925 | 14.905 | 0.000499 | 0.007514 | 3.897725 |
| LOC103392786 | 4.562852 | 0.0005 | 0.007521 | 2.189936 |
| LOC107988498 | 7.69539 | 0.000501 | 0.007526 | 2.943994 |
| LOC103380971 | 0.065841 | 0.000502 | 0.00753 | 3.924871 |
| LOC103395279 | 27.32946 | 0.000503 | 0.007535 | 4.772385 |
| dnaaf2 | 13.42901 | 0.000503 | 0.007535 | 3.747281 |
| LOC103387271 | 0.227158 | 0.000505 | 0.00755 | 2.138232 |
| trnap-cgg | 9.599281 | 0.000512 | 0.007623 | 3.262926 |
| LOC103381411 | 4.307763 | 0.000512 | 0.007628 | 2.106939 |
| etv1 | 8.306328 | 0.000515 | 0.007652 | 3.054211 |
| LOC103392359 | 0.177885 | 0.000515 | 0.007658 | 2.490982 |
| slc7a14 | 0.085935 | 0.000519 | 0.007665 | 3.540611 |
| tmem150b | 7.238887 | 0.000519 | 0.007665 | 2.855768 |
| LOC103378717 | 0.138218 | 0.000517 | 0.007665 | 2.854986 |
| LOC103380607 | 7.225865 | 0.00052 | 0.007665 | 2.85317 |
| LOC103398216 | 0.162471 | 0.000519 | 0.007665 | 2.621749 |
| LOC103385771 | 5.425891 | 0.00052 | 0.007665 | 2.43986 |
| LOC103378645 | 0.224239 | 0.00052 | 0.007665 | 2.15689 |
| cacul1 | 9.426295 | 0.000521 | 0.00767 | 3.236691 |
| LOC103384016 | 4.253143 | 0.000521 | 0.00767 | 2.088529 |
| LOC103396417 | 0.135061 | 0.000523 | 0.007682 | 2.888313 |
| LOC103391171 | 55.77146 | 0.000527 | 0.007687 | 5.801455 |
| LOC103387079 | 54.36438 | 0.000526 | 0.007687 | 5.76459 |
| naa60 | 8.203925 | 0.000525 | 0.007687 | 3.036314 |
| akip1 | 5.709157 | 0.000524 | 0.007687 | 2.513278 |
| LOC103385485 | 5.549258 | 0.000532 | 0.007738 | 2.472295 |
| LOC103396843 | 5.042268 | 0.000534 | 0.007744 | 2.334073 |
| LOC103377937 | 14.53062 | 0.000536 | 0.007754 | 3.861024 |
| LOC103392524 | 42.66264 | 0.000541 | 0.007805 | 5.414901 |
| atg4b | 6.407696 | 0.00054 | 0.007805 | 2.679806 |
| podxl | 0.217973 | 0.000541 | 0.007805 | 2.197779 |
| LOC103378320 | 7.147259 | 0.000543 | 0.007813 | 2.83739 |
| orc4 | 0.194665 | 0.000547 | 0.007861 | 2.360931 |
| dpy19l3 | 0.222195 | 0.000548 | 0.007866 | 2.170102 |
| LOC103387589 | 0.133148 | 0.000551 | 0.007887 | 2.908899 |
| ndrg2 | 0.156356 | 0.000553 | 0.007898 | 2.67709 |
| LOC103381346 | 0.19819 | 0.000552 | 0.007898 | 2.335046 |
| heatr1 | 0.137893 | 0.000555 | 0.00792 | 2.858375 |
| LOC103397429 | 0.132077 | 0.000558 | 0.007946 | 2.920554 |
| adprm | 0.179994 | 0.000564 | 0.008007 | 2.473978 |
| adgra1 | 0.200025 | 0.000565 | 0.008012 | 2.321751 |
| csrnp1 | 0.10504 | 0.000566 | 0.008012 | 3.250995 |
| LOC103384251 | 8.665817 | 0.000567 | 0.008012 | 3.115336 |
| rpusd2 | 0.170218 | 0.000568 | 0.008012 | 2.554543 |
| nxpe3 | 4.378294 | 0.000567 | 0.008012 | 2.130369 |
| LOC103389486 | 0.24737 | 0.000567 | 0.008012 | 2.015255 |
| LOC103383089 | 4.40965 | 0.000572 | 0.008056 | 2.140664 |
| spata17 | 11.89857 | 0.000574 | 0.008068 | 3.572716 |
| LOC103379809 | 19.82222 | 0.000581 | 0.008136 | 4.309047 |
| nufip1 | 0.197651 | 0.000584 | 0.008153 | 2.338971 |
| LOC103395430 | 4.618197 | 0.000594 | 0.008256 | 2.20733 |
| cfap47 | 6.190643 | 0.000595 | 0.008265 | 2.630089 |
| LOC103396241 | 19.66119 | 0.000597 | 0.008284 | 4.297279 |
| zc3h3 | 4.301108 | 0.000597 | 0.008284 | 2.104708 |
| LOC103377545 | 8.811165 | 0.000599 | 0.008306 | 3.139333 |
| LOC112487808 | 10.03383 | 0.0006 | 0.008306 | 3.326801 |
| prph | 0.24134 | 0.000601 | 0.008319 | 2.050861 |
| atg13 | 5.114776 | 0.00061 | 0.008408 | 2.354671 |
| LOC103393060 | 5.695609 | 0.000613 | 0.008427 | 2.50985 |
| fkbp8 | 4.96282 | 0.000613 | 0.008427 | 2.31116 |
| LOC107990080 | 14.46487 | 0.000614 | 0.008435 | 3.854482 |
| LOC103394946 | 44.15575 | 0.000614 | 0.00844 | 5.464529 |
| LOC112487464 | 0.217068 | 0.000617 | 0.00846 | 2.203783 |
| LOC103388329 | 0.186098 | 0.000618 | 0.008463 | 2.425869 |
| ky | 14.33764 | 0.000619 | 0.008469 | 3.841736 |
| aste1 | 8.820659 | 0.000623 | 0.008488 | 3.140886 |
| LOC103383590 | 0.208837 | 0.000622 | 0.008488 | 2.259554 |
| ermp1 | 4.070956 | 0.000622 | 0.008488 | 2.025368 |
| faxdc2 | 5.940886 | 0.000624 | 0.008492 | 2.570678 |
| LOC103376640 | 4.951691 | 0.000624 | 0.008492 | 2.307921 |
| LOC103395674 | 19.38426 | 0.00063 | 0.008542 | 4.276813 |
| LOC103395983 | 6.201089 | 0.000631 | 0.008544 | 2.632522 |
| LOC103397032 | 5.449349 | 0.000631 | 0.008544 | 2.446084 |
| ebp | 0.130229 | 0.000636 | 0.008573 | 2.94088 |
| creg2 | 0.161626 | 0.000637 | 0.008587 | 2.629271 |
| LOC112487913 | 4.976815 | 0.000637 | 0.008587 | 2.315223 |
| LOC103388321 | 0.181114 | 0.00064 | 0.008612 | 2.465028 |
| trnar-acg | 23.44087 | 0.000642 | 0.008621 | 4.550954 |
| LOC103389650 | 41.5157 | 0.000643 | 0.008624 | 5.375585 |
| LOC103379046 | 0.165698 | 0.000644 | 0.008629 | 2.593376 |
| hsd3b7 | 14.62628 | 0.000646 | 0.008637 | 3.87049 |
| LOC103381121 | 11.20415 | 0.000647 | 0.008637 | 3.485961 |
| arl2bp | 11.01483 | 0.000646 | 0.008637 | 3.461375 |
| LOC103385984 | 5.370582 | 0.000646 | 0.008637 | 2.425078 |
| LOC103388880 | 11.2823 | 0.000649 | 0.008648 | 3.49599 |
| eva1a | 5.612067 | 0.00065 | 0.008648 | 2.488532 |
| LOC103383792 | 0.22695 | 0.00065 | 0.008648 | 2.139555 |
| spast | 4.119105 | 0.000648 | 0.008648 | 2.042331 |
| LOC103387105 | 4.302049 | 0.000652 | 0.008669 | 2.105024 |
| akap13 | 4.694523 | 0.000656 | 0.008673 | 2.230978 |
| LOC112487839 | 0.229267 | 0.000655 | 0.008673 | 2.124899 |
| LOC103384676 | 0.132955 | 0.000659 | 0.008696 | 2.910988 |
| LOC103394383 | 5.937348 | 0.000665 | 0.008727 | 2.569819 |
| LOC103384195 | 5.60015 | 0.000667 | 0.008741 | 2.485466 |
| LOC103386166 | 4.034572 | 0.000671 | 0.008784 | 2.012416 |
| LOC103376631 | 0.056176 | 0.000679 | 0.008854 | 4.15389 |
| wdr43 | 0.225861 | 0.000684 | 0.008904 | 2.146495 |
| LOC103382005 | 0.122346 | 0.000687 | 0.008916 | 3.03096 |
| LOC112487990 | 0.162021 | 0.000687 | 0.008916 | 2.625747 |
| c2h8orf82 | 4.698931 | 0.000688 | 0.008916 | 2.232333 |
| LOC103398027 | 28.5473 | 0.000696 | 0.008953 | 4.835282 |
| LOC103388349 | 5.789805 | 0.000695 | 0.008953 | 2.533515 |
| LOC103394116 | 0.180673 | 0.000695 | 0.008953 | 2.468549 |
| LOC103395405 | 4.951073 | 0.000692 | 0.008953 | 2.307741 |
| slc38a9 | 4.074778 | 0.000692 | 0.008953 | 2.026722 |
| xrn2 | 4.693439 | 0.000701 | 0.008987 | 2.230645 |
| gemin5 | 4.449392 | 0.000702 | 0.008993 | 2.153608 |
| lrsam1 | 5.712019 | 0.000707 | 0.009025 | 2.514001 |
| LOC103396359 | 0.186637 | 0.000711 | 0.009063 | 2.421691 |
| LOC112487058 | 5.015421 | 0.000712 | 0.009075 | 2.326371 |
| ttbk2 | 4.049605 | 0.000715 | 0.009093 | 2.017781 |
| LOC103384233 | 24.29722 | 0.000715 | 0.009093 | 4.60272 |
| tmem138 | 4.999527 | 0.000719 | 0.009124 | 2.321791 |
| LOC103379602 | 7.311251 | 0.00072 | 0.009135 | 2.870118 |
| id2 | 0.090686 | 0.000724 | 0.009149 | 3.462978 |
| LOC103386954 | 0.162543 | 0.000725 | 0.009149 | 2.621103 |
| kcnj2 | 0.180942 | 0.000724 | 0.009149 | 2.466399 |
| LOC103395883 | 0.132829 | 0.000726 | 0.009152 | 2.91236 |
| LOC103396920 | 6.138569 | 0.000726 | 0.009152 | 2.617902 |
| LOC103388934 | 4.602128 | 0.000727 | 0.009154 | 2.202301 |
| LOC103376940 | 0.044218 | 0.000729 | 0.009159 | 4.499229 |
| LOC103390348 | 0.151204 | 0.000729 | 0.009159 | 2.725436 |
| LOC103388562 | 5.414964 | 0.00073 | 0.009159 | 2.436952 |
| LOC103382183 | 10.99416 | 0.000733 | 0.009184 | 3.458666 |
| LOC103381450 | 9.990382 | 0.000734 | 0.009184 | 3.32054 |
| LOC103390542 | 0.191062 | 0.000734 | 0.009184 | 2.387887 |
| phf6 | 0.149862 | 0.00074 | 0.009227 | 2.738297 |
| prkar1b | 6.255473 | 0.000743 | 0.009238 | 2.645119 |
| LOC103382986 | 6.495057 | 0.000745 | 0.009258 | 2.699342 |
| LOC103384033 | 93.34873 | 0.00075 | 0.009296 | 6.544558 |
| LOC103384754 | 11.90063 | 0.000751 | 0.0093 | 3.572966 |
| LOC103378551 | 100.1957 | 0.000758 | 0.009324 | 6.646677 |
| LOC103382631 | 8.603147 | 0.000757 | 0.009324 | 3.104864 |
| LOC103377404 | 8.564037 | 0.000759 | 0.009327 | 3.098291 |
| LOC103386304 | 10.99384 | 0.00076 | 0.009328 | 3.458623 |
| naaa | 0.119675 | 0.00076 | 0.009328 | 3.062802 |
| LOC107988589 | 17.07514 | 0.000761 | 0.009331 | 4.093825 |
| LOC103389615 | 0.171919 | 0.000764 | 0.009349 | 2.540202 |
| LOC112487973 | 4.803844 | 0.000766 | 0.009363 | 2.264189 |
| LOC103397562 | 0.209986 | 0.000767 | 0.009363 | 2.251632 |
| LOC103395794 | 11.82869 | 0.000769 | 0.009375 | 3.564218 |
| LOC103397061 | 0.185132 | 0.00077 | 0.009378 | 2.433376 |
| LOC103382891 | 4.852615 | 0.00077 | 0.009378 | 2.278762 |
| hmga1 | 0.154065 | 0.000773 | 0.009393 | 2.69839 |
| LOC103391190 | 0.123396 | 0.000775 | 0.009395 | 3.018633 |
| cfap54 | 7.378582 | 0.000774 | 0.009395 | 2.883344 |
| sh2d5 | 0.180365 | 0.000776 | 0.009395 | 2.471008 |
| tcf4 | 0.203237 | 0.00078 | 0.009429 | 2.298768 |
| LOC103384359 | 16.24581 | 0.000787 | 0.009485 | 4.021995 |
| LOC103392534 | 0.168334 | 0.000787 | 0.009487 | 2.570601 |
| LOC103384633 | 30.56754 | 0.000789 | 0.009493 | 4.933929 |
| scpep1 | 0.07917 | 0.00079 | 0.009494 | 3.658895 |
| LOC107989489 | 10.78054 | 0.00079 | 0.009494 | 3.430358 |
| ric8a | 5.002169 | 0.000791 | 0.009495 | 2.322554 |
| mos | 0.063507 | 0.000792 | 0.009502 | 3.976942 |
| LOC103377325 | 0.062214 | 0.000794 | 0.009508 | 4.006607 |
| LOC103386842 | 4.854551 | 0.000801 | 0.009566 | 2.279338 |
| sema6d | 11.53744 | 0.000804 | 0.009577 | 3.528251 |
| LOC103393527 | 4.986177 | 0.000807 | 0.009609 | 2.317934 |
| LOC112487040 | 18.4499 | 0.000809 | 0.009612 | 4.205541 |
| LOC103386518 | 6.834698 | 0.000809 | 0.009612 | 2.772878 |
| LOC103385726 | 0.181448 | 0.000814 | 0.009651 | 2.462373 |
| LOC112487758 | 8.931026 | 0.000818 | 0.009693 | 3.158826 |
| LOC103382298 | 11.76115 | 0.00082 | 0.009709 | 3.555958 |
| LOC103396820 | 5.977704 | 0.000821 | 0.009709 | 2.579591 |
| cd34 | 0.187689 | 0.000825 | 0.009738 | 2.413581 |
| LOC103382499 | 0.177731 | 0.00083 | 0.009763 | 2.492235 |
| tubb | 0.181239 | 0.000828 | 0.009763 | 2.464038 |
| prdm12 | 4.51402 | 0.000829 | 0.009763 | 2.174413 |
| LOC103396111 | 5.69127 | 0.000834 | 0.009796 | 2.508751 |
| tes | 0.092608 | 0.000835 | 0.0098 | 3.43272 |
| kdf1 | 0.240639 | 0.00084 | 0.009833 | 2.055056 |
| LOC103388044 | 0.143023 | 0.000841 | 0.00984 | 2.805676 |
| faim | 4.853686 | 0.000842 | 0.009844 | 2.279081 |
| tmem81 | 7.385383 | 0.000843 | 0.00985 | 2.884673 |
| LOC103391218 | 16.13569 | 0.000846 | 0.009866 | 4.012183 |
| LOC103381101 | 0.109554 | 0.000845 | 0.009866 | 3.190288 |
| LOC103392525 | 11.57847 | 0.00085 | 0.009889 | 3.533373 |
| LOC103399832 | 13.872 | 0.000851 | 0.009889 | 3.794104 |
| caprin2 | 0.176935 | 0.000851 | 0.00989 | 2.498712 |
| LOC103384881 | 4.67818 | 0.000855 | 0.009904 | 2.225947 |
| LOC103384460 | 13.38545 | 0.000862 | 0.009908 | 3.742594 |
| LOC103382528 | 7.821975 | 0.000858 | 0.009908 | 2.967533 |
| LOC103388644 | 6.233661 | 0.000857 | 0.009908 | 2.64008 |
| txndc9 | 4.282796 | 0.000861 | 0.009908 | 2.098553 |
| c6h16orf70 | 4.180798 | 0.000862 | 0.009908 | 2.063778 |
| LOC103384447 | 0.215159 | 0.000866 | 0.009932 | 2.216528 |
| LOC103387240 | 8.152289 | 0.000867 | 0.00994 | 3.027205 |
| LOC103378423 | 7.738554 | 0.000867 | 0.00994 | 2.952064 |
| LOC103390456 | 12.69242 | 0.000871 | 0.009952 | 3.665895 |
| LOC103395377 | 4.547144 | 0.000875 | 0.009974 | 2.184961 |
| brinp3 | 0.201847 | 0.000877 | 0.009978 | 2.308667 |
| LOC103393893 | 0.227309 | 0.000878 | 0.009979 | 2.137272 |
| LOC107990165 | 5.850596 | 0.000884 | 0.010015 | 2.548584 |
| LOC103382067 | 7.383268 | 0.000894 | 0.010079 | 2.88426 |
| slc2a10 | 4.984281 | 0.000897 | 0.010092 | 2.317385 |
| trappc9 | 0.196183 | 0.000903 | 0.010141 | 2.349728 |
| LOC103383535 | 7.817962 | 0.000909 | 0.010168 | 2.966793 |
| LOC103387820 | 5.520025 | 0.000911 | 0.010182 | 2.464675 |
| LOC112488190 | 0.203075 | 0.000912 | 0.010182 | 2.299916 |
| ube2ql1 | 22.24957 | 0.000921 | 0.010244 | 4.475706 |
| LOC103382596 | 4.966169 | 0.000921 | 0.010244 | 2.312133 |
| ccnj | 0.244619 | 0.000921 | 0.010244 | 2.03139 |
| LOC103397017 | 6.085094 | 0.000923 | 0.010244 | 2.60528 |
| hdac11 | 5.036368 | 0.000925 | 0.010245 | 2.332384 |
| LOC103382903 | 7.612971 | 0.000927 | 0.010253 | 2.92846 |
| LOC103380118 | 0.197455 | 0.000927 | 0.010253 | 2.340403 |
| phf21a | 4.514552 | 0.000927 | 0.010253 | 2.174583 |
| ddo | 0.220054 | 0.000931 | 0.010254 | 2.18407 |
| LOC103379452 | 8.955945 | 0.000932 | 0.010255 | 3.162846 |
| ttc27 | 0.147202 | 0.000936 | 0.010274 | 2.764129 |
| LOC103379509 | 10.90168 | 0.000938 | 0.010279 | 3.446479 |
| LOC103384513 | 0.184383 | 0.00094 | 0.010279 | 2.439223 |
| braf | 0.225735 | 0.000939 | 0.010279 | 2.147295 |
| LOC103396385 | 4.065077 | 0.000939 | 0.010279 | 2.023283 |
| LOC103383361 | 0.220079 | 0.000951 | 0.010362 | 2.18391 |
| tra2b | 7.536898 | 0.000952 | 0.010364 | 2.913971 |
| LOC103395671 | 14.49196 | 0.000958 | 0.010398 | 3.857181 |
| rsph9 | 12.08572 | 0.000968 | 0.01046 | 3.595231 |
| LOC103378351 | 0.046404 | 0.00097 | 0.010468 | 4.429598 |
| LOC112487980 | 0.092483 | 0.000975 | 0.010504 | 3.434673 |
| col9a2 | 11.0614 | 0.000978 | 0.010531 | 3.467462 |
| LOC107988305 | 15.47053 | 0.000982 | 0.010551 | 3.951451 |
| LOC103387168 | 8.101108 | 0.000983 | 0.010551 | 3.018119 |
| rnf43 | 7.392807 | 0.000982 | 0.010551 | 2.886122 |
| glt8d2 | 6.766217 | 0.000983 | 0.010551 | 2.75835 |
| LOC103396193 | 0.238321 | 0.000983 | 0.010551 | 2.069021 |
| LOC103393414 | 0.112275 | 0.000984 | 0.010553 | 3.15489 |
| c19h11orf54 | 0.205119 | 0.000986 | 0.010562 | 2.285465 |
| LOC103376681 | 4.901682 | 0.000988 | 0.010565 | 2.293277 |
| abhd16a | 4.284089 | 0.000992 | 0.010581 | 2.098989 |
| zfc3h1 | 4.449944 | 0.000994 | 0.010591 | 2.153787 |
| otor | 0.223799 | 0.000996 | 0.0106 | 2.159726 |
| LOC103395914 | 4.300829 | 0.000999 | 0.010616 | 2.104615 |
| LOC103394036 | 0.208085 | 0.001 | 0.01062 | 2.264753 |
| LOC107988558 | 0.241217 | 0.001001 | 0.010623 | 2.051594 |
| LOC103384614 | 0.188601 | 0.001005 | 0.010662 | 2.406592 |
| LOC103388459 | 12.73236 | 0.001005 | 0.010662 | 3.670428 |
| crtam | 6.516738 | 0.001007 | 0.010668 | 2.70415 |
| LOC103386949 | 5.871975 | 0.001011 | 0.010682 | 2.553846 |
| LOC103387312 | 4.307123 | 0.00101 | 0.010682 | 2.106724 |
| tbl2 | 0.243957 | 0.001012 | 0.010682 | 2.035304 |
| rgs22 | 4.091215 | 0.001012 | 0.010682 | 2.032529 |
| LOC112488542 | 17.44508 | 0.001015 | 0.010694 | 4.124748 |
| aldh3b1 | 0.126041 | 0.001015 | 0.010694 | 2.988031 |
| usp12 | 4.167333 | 0.001018 | 0.01071 | 2.059124 |
| LOC103394223 | 6.63264 | 0.001018 | 0.010713 | 2.729583 |
| LOC112486867 | 5.135445 | 0.00102 | 0.010717 | 2.360489 |
| LOC103396027 | 0.201533 | 0.001025 | 0.010747 | 2.310914 |
| LOC103393778 | 18.02869 | 0.001026 | 0.010751 | 4.172223 |
| LOC103384247 | 4.053682 | 0.001027 | 0.010752 | 2.019233 |
| slc24a4 | 0.066106 | 0.00103 | 0.010766 | 3.919069 |
| LOC103382292 | 0.123554 | 0.001031 | 0.010767 | 3.016786 |
| LOC103377694 | 7.942723 | 0.001035 | 0.010788 | 2.989634 |
| LOC103386312 | 5.844694 | 0.001035 | 0.010788 | 2.547128 |
| LOC103389673 | 0.071918 | 0.001045 | 0.01085 | 3.7975 |
| LOC103381128 | 8.869017 | 0.001045 | 0.01085 | 3.148774 |
| trnar-ucg | 6.843035 | 0.001046 | 0.01085 | 2.774636 |
| LOC103378947 | 0.239767 | 0.001046 | 0.01085 | 2.060293 |
| LOC103392800 | 0.247688 | 0.001046 | 0.01085 | 2.013407 |
| LOC103385575 | 8.479288 | 0.001051 | 0.010888 | 3.083943 |
| cep44 | 0.184122 | 0.001055 | 0.01089 | 2.441263 |
| dock11 | 0.205619 | 0.001055 | 0.01089 | 2.281952 |
| LOC103379613 | 0.128722 | 0.001056 | 0.010896 | 2.957669 |
| zdhhc21 | 5.303125 | 0.001058 | 0.010907 | 2.406843 |
| LOC103386591 | 9.176213 | 0.001059 | 0.010909 | 3.197899 |
| LOC103388542 | 0.230354 | 0.001061 | 0.010912 | 2.118078 |
| LOC103389108 | 5.147915 | 0.001065 | 0.010936 | 2.363988 |
| LOC112487019 | 4.072553 | 0.001067 | 0.010945 | 2.025933 |
| LOC103394292 | 8.488908 | 0.001068 | 0.010947 | 3.085579 |
| LOC103399858 | 0.237627 | 0.001068 | 0.010947 | 2.073232 |
| spef2 | 10.17832 | 0.001072 | 0.01097 | 3.347427 |
| LOC103395174 | 22.05881 | 0.001083 | 0.011002 | 4.463283 |
| LOC103397251 | 15.57764 | 0.001082 | 0.011002 | 3.961405 |
| LOC103384446 | 6.952124 | 0.001082 | 0.011002 | 2.797454 |
| larp6 | 0.1098 | 0.001085 | 0.011012 | 3.187056 |
| spag1 | 6.799011 | 0.001091 | 0.011055 | 2.765325 |
| LOC103386926 | 4.805663 | 0.001093 | 0.011055 | 2.264735 |
| zbtb1 | 0.210276 | 0.001093 | 0.011055 | 2.249642 |
| LOC103377788 | 6.5417 | 0.001098 | 0.011087 | 2.709666 |
| prrt1 | 10.17626 | 0.0011 | 0.011087 | 3.347136 |
| LOC103379018 | 0.058756 | 0.001106 | 0.011131 | 4.08913 |
| LOC103396326 | 6.000299 | 0.001106 | 0.011131 | 2.585034 |
| LOC103378290 | 6.58043 | 0.00111 | 0.011139 | 2.718182 |
| LOC103389898 | 0.142797 | 0.001114 | 0.011145 | 2.80796 |
| LOC103394786 | 0.238383 | 0.001117 | 0.011166 | 2.068649 |
| LOC103394183 | 0.144044 | 0.001121 | 0.011191 | 2.795422 |
| kcnh8 | 4.660252 | 0.001122 | 0.011193 | 2.220408 |
| dmrt1 | 5.085543 | 0.001124 | 0.011206 | 2.346402 |
| LOC103388102 | 8.330517 | 0.00113 | 0.011238 | 3.058406 |
| yod1 | 4.057495 | 0.001134 | 0.011254 | 2.020589 |
| LOC103393500 | 38.05504 | 0.001138 | 0.011259 | 5.250016 |
| ctxnd1 | 12.97071 | 0.00114 | 0.011276 | 3.697186 |
| LOC103378457 | 0.186116 | 0.00114 | 0.011276 | 2.425726 |
| LOC103389489 | 6.606154 | 0.001148 | 0.011335 | 2.723811 |
| LOC103397924 | 5.485028 | 0.001149 | 0.011335 | 2.455499 |
| slc8b1 | 0.096621 | 0.001152 | 0.011342 | 3.371522 |
| togaram1 | 10.25906 | 0.001152 | 0.011342 | 3.358827 |
| LOC103379772 | 0.168722 | 0.001152 | 0.011342 | 2.567279 |
| amt | 0.155134 | 0.001162 | 0.011404 | 2.688409 |
| map2k2 | 0.216546 | 0.001166 | 0.011436 | 2.207252 |
| arl1 | 4.714893 | 0.00117 | 0.011459 | 2.237225 |
| LOC103385354 | 0.180147 | 0.001181 | 0.011502 | 2.472751 |
| LOC103396811 | 0.167217 | 0.001183 | 0.011514 | 2.580206 |
| ak1 | 7.334385 | 0.0012 | 0.011603 | 2.874676 |
| LOC103377603 | 7.305888 | 0.0012 | 0.011603 | 2.86906 |
| errfi1 | 0.176512 | 0.0012 | 0.011603 | 2.502165 |
| LOC103397814 | 0.148851 | 0.001204 | 0.011627 | 2.748055 |
| cdt1 | 0.102693 | 0.001206 | 0.011634 | 3.283587 |
| LOC112486483 | 6.318804 | 0.001209 | 0.011653 | 2.659652 |
| LOC103381660 | 0.176221 | 0.001213 | 0.011681 | 2.504544 |
| LOC103389815 | 4.099223 | 0.001223 | 0.011714 | 2.03535 |
| nrdc | 4.062875 | 0.001223 | 0.011714 | 2.022501 |
| tiparp | 0.20265 | 0.001224 | 0.011717 | 2.30294 |
| LOC103394289 | 25.27406 | 0.001228 | 0.011732 | 4.659585 |
| parp1 | 0.207283 | 0.001231 | 0.011745 | 2.270327 |
| kank1 | 0.189767 | 0.001234 | 0.011759 | 2.3977 |
| tesk1 | 0.060605 | 0.001237 | 0.011766 | 4.04441 |
| LOC103397689 | 0.220174 | 0.001236 | 0.011766 | 2.183283 |
| phospho1 | 5.541853 | 0.001244 | 0.011802 | 2.470368 |
| LOC103392857 | 0.218272 | 0.001248 | 0.011824 | 2.195802 |
| cyhr1 | 9.895711 | 0.001251 | 0.011851 | 3.306803 |
| LOC103382282 | 5.119542 | 0.001252 | 0.011853 | 2.356015 |
| soga3 | 0.197104 | 0.001254 | 0.011863 | 2.34297 |
| LOC103378032 | 4.925801 | 0.001254 | 0.011863 | 2.300358 |
| LOC103389228 | 0.241007 | 0.001255 | 0.011864 | 2.052854 |
| LOC103393202 | 0.235713 | 0.001273 | 0.011973 | 2.084894 |
| LOC103383507 | 0.149333 | 0.001276 | 0.011988 | 2.743392 |
| LOC103377886 | 0.221823 | 0.001279 | 0.012001 | 2.172522 |
| fam219b | 4.182793 | 0.001287 | 0.012033 | 2.064466 |
| kctd14 | 0.137486 | 0.001291 | 0.01206 | 2.86264 |
| LOC103378873 | 16.27498 | 0.001293 | 0.012064 | 4.024584 |
| medag | 0.183636 | 0.001299 | 0.012093 | 2.445078 |
| radil | 0.249927 | 0.001299 | 0.012093 | 2.000419 |
| LOC103377781 | 12.92937 | 0.001304 | 0.012128 | 3.69258 |
| LOC103380040 | 0.129624 | 0.00131 | 0.012145 | 2.947599 |
| LOC103379725 | 4.913855 | 0.001311 | 0.012145 | 2.296855 |
| rb1cc1 | 4.039459 | 0.001314 | 0.012168 | 2.014162 |
| LOC103378288 | 25.52843 | 0.001315 | 0.012172 | 4.674033 |
| rbfox3 | 0.176055 | 0.001323 | 0.012201 | 2.505898 |
| kcnc4 | 0.230835 | 0.001331 | 0.012239 | 2.115069 |
| LOC103397415 | 0.227786 | 0.001334 | 0.012262 | 2.134247 |
| LOC103394835 | 8.976552 | 0.001341 | 0.012289 | 3.166161 |
| slc6a4 | 0.24212 | 0.001341 | 0.012289 | 2.046205 |
| dhrs3 | 0.096581 | 0.001344 | 0.012298 | 3.372121 |
| LOC103396140 | 7.007637 | 0.001344 | 0.012298 | 2.808928 |
| LOC103396287 | 0.147621 | 0.001355 | 0.012337 | 2.760027 |
| snx25 | 5.68274 | 0.001355 | 0.012337 | 2.506587 |
| LOC103385444 | 6.826487 | 0.001364 | 0.012381 | 2.771143 |
| ctsc | 0.147993 | 0.001364 | 0.012381 | 2.756401 |
| LOC103384489 | 16.23914 | 0.001372 | 0.012425 | 4.021404 |
| LOC103391271 | 5.53755 | 0.001373 | 0.012428 | 2.469248 |
| LOC107989156 | 30.38511 | 0.001375 | 0.012437 | 4.925292 |
| LOC103382505 | 4.061589 | 0.001377 | 0.012451 | 2.022044 |
| mindy1 | 4.268152 | 0.001382 | 0.012476 | 2.093611 |
| LOC103378594 | 11.67177 | 0.001387 | 0.012494 | 3.544952 |
| LOC103389659 | 9.982387 | 0.001387 | 0.012494 | 3.319385 |
| pus7l | 7.474984 | 0.00139 | 0.012517 | 2.90207 |
| fech | 0.238965 | 0.001393 | 0.012522 | 2.06513 |
| LOC103385063 | 34.7503 | 0.0014 | 0.012556 | 5.118953 |
| fam189a1 | 0.235332 | 0.001401 | 0.012556 | 2.08723 |
| pdcd11 | 0.185449 | 0.001402 | 0.012561 | 2.430903 |
| tbce | 4.87775 | 0.001402 | 0.012561 | 2.286216 |
| LOC107988359 | 6.583062 | 0.001405 | 0.012578 | 2.718759 |
| LOC103392637 | 6.070598 | 0.001408 | 0.012586 | 2.601839 |
| spaca6 | 11.03765 | 0.001409 | 0.01259 | 3.464361 |
| LOC103384218 | 0.245332 | 0.001409 | 0.01259 | 2.027194 |
| LOC103386083 | 0.221435 | 0.001414 | 0.012617 | 2.175046 |
| LOC103392785 | 5.557546 | 0.001433 | 0.012696 | 2.474448 |
| LOC103395264 | 4.044929 | 0.001434 | 0.012696 | 2.016114 |
| LOC103386405 | 11.08245 | 0.001437 | 0.012704 | 3.470205 |
| pdss1 | 0.247134 | 0.001453 | 0.012798 | 2.016636 |
| LOC103392216 | 0.192616 | 0.001459 | 0.01282 | 2.376202 |
| LOC112487583 | 15.00567 | 0.00146 | 0.01282 | 3.907435 |
| LOC103390657 | 0.198448 | 0.00146 | 0.01282 | 2.333167 |
| LOC103385066 | 9.155323 | 0.001469 | 0.012868 | 3.194611 |
| nsf | 5.504642 | 0.001476 | 0.012903 | 2.460649 |
| spag17 | 12.82313 | 0.001481 | 0.012924 | 3.680676 |
| LOC103382514 | 8.20522 | 0.001482 | 0.012924 | 3.036542 |
| LOC103394204 | 12.94993 | 0.001487 | 0.012957 | 3.694873 |
| panx1 | 5.695183 | 0.001488 | 0.012957 | 2.509742 |
| LOC103385543 | 7.551703 | 0.001495 | 0.012999 | 2.916802 |
| tmem51 | 0.1651 | 0.001494 | 0.012999 | 2.598589 |
| LOC112487152 | 0.23622 | 0.001496 | 0.013 | 2.081797 |
| LOC103393217 | 0.191453 | 0.001499 | 0.013013 | 2.384941 |
| phf24 | 4.220294 | 0.001501 | 0.013027 | 2.077343 |
| LOC103376825 | 4.85781 | 0.001502 | 0.01303 | 2.280306 |
| LOC103388948 | 0.220061 | 0.001505 | 0.013049 | 2.184022 |
| LOC103379724 | 0.154436 | 0.001507 | 0.013051 | 2.694922 |
| asxl2 | 6.534698 | 0.001511 | 0.013068 | 2.708121 |
| LOC103394620 | 0.116581 | 0.001512 | 0.013074 | 3.100599 |
| leprotl1 | 4.3429 | 0.001518 | 0.013094 | 2.118659 |
| foxred1 | 4.384112 | 0.001521 | 0.013096 | 2.132285 |
| atad2 | 7.496311 | 0.001523 | 0.013097 | 2.906181 |
| LOC103376847 | 81.3505 | 0.001527 | 0.013111 | 6.346079 |
| LOC103394933 | 0.248802 | 0.00153 | 0.013125 | 2.006933 |
| LOC103385930 | 5.532812 | 0.001536 | 0.013139 | 2.468013 |
| dtl | 0.228962 | 0.00154 | 0.013153 | 2.126819 |
| c17h11orf88 | 5.94929 | 0.001547 | 0.013194 | 2.572718 |
| LOC103397671 | 0.228383 | 0.001546 | 0.013194 | 2.130471 |
| rsph1 | 80.22434 | 0.001553 | 0.01323 | 6.325968 |
| LOC112486254 | 0.206645 | 0.001567 | 0.013291 | 2.274776 |
| LOC112486784 | 29.57115 | 0.001572 | 0.013321 | 4.886118 |
| LOC107988571 | 5.036482 | 0.00158 | 0.013356 | 2.332416 |
| LOC103385166 | 7.725601 | 0.001581 | 0.013357 | 2.949647 |
| siva1 | 0.228439 | 0.001594 | 0.013431 | 2.130121 |
| acsl6 | 5.627584 | 0.001595 | 0.01344 | 2.492516 |
| luzp2 | 0.160687 | 0.001597 | 0.013443 | 2.637675 |
| gabrd | 6.18147 | 0.001602 | 0.013467 | 2.62795 |
| LOC103392616 | 5.172873 | 0.001604 | 0.013467 | 2.370966 |
| LOC103398036 | 0.208312 | 0.001612 | 0.013489 | 2.263184 |
| LOC103395822 | 0.094532 | 0.001617 | 0.013508 | 3.403055 |
| c18h1orf43 | 4.120731 | 0.00162 | 0.013525 | 2.0429 |
| LOC103384774 | 19.88199 | 0.00162 | 0.013527 | 4.31339 |
| LOC103381105 | 13.04437 | 0.001626 | 0.013549 | 3.705355 |
| LOC103382762 | 4.379471 | 0.00163 | 0.013558 | 2.130757 |
| LOC103385593 | 8.953827 | 0.001633 | 0.013568 | 3.162504 |
| lrguk | 5.6096 | 0.001634 | 0.013568 | 2.487898 |
| snorc | 0.203933 | 0.001632 | 0.013568 | 2.293831 |
| LOC103378788 | 5.642051 | 0.001642 | 0.013608 | 2.49622 |
| g6pc3 | 4.83733 | 0.00164 | 0.013608 | 2.274211 |
| tph2 | 4.237675 | 0.001643 | 0.013611 | 2.083273 |
| LOC103394294 | 0.108644 | 0.001645 | 0.01362 | 3.202321 |
| LOC103394873 | 0.138175 | 0.001645 | 0.01362 | 2.855431 |
| trnaa-ugc | 10.44612 | 0.001647 | 0.013626 | 3.384895 |
| LOC103385383 | 7.827358 | 0.001653 | 0.013668 | 2.968525 |
| LOC103388571 | 22.33286 | 0.001657 | 0.013678 | 4.481096 |
| rnf151 | 4.245872 | 0.001658 | 0.013678 | 2.086061 |
| mterf3 | 0.240254 | 0.00166 | 0.013678 | 2.057371 |
| bora | 0.24962 | 0.001671 | 0.013732 | 2.002196 |
| paqr9 | 5.575373 | 0.001672 | 0.013737 | 2.479068 |
| LOC103392215 | 0.232999 | 0.001674 | 0.013743 | 2.101604 |
| lama3 | 0.131684 | 0.001677 | 0.013747 | 2.924844 |
| LOC112487924 | 5.456637 | 0.001691 | 0.013793 | 2.448012 |
| LOC103384104 | 60.56191 | 0.001703 | 0.013847 | 5.920339 |
| LOC112488257 | 4.02626 | 0.001711 | 0.013883 | 2.00944 |
| myrip | 4.231757 | 0.001716 | 0.013888 | 2.081257 |
| LOC103376887 | 0.239759 | 0.001723 | 0.013932 | 2.060344 |
| LOC103393612 | 0.160388 | 0.001726 | 0.013935 | 2.640365 |
| fhod1 | 0.196579 | 0.001726 | 0.013935 | 2.346816 |
| LOC103387355 | 6.134791 | 0.001729 | 0.013944 | 2.617014 |
| LOC103381630 | 0.242862 | 0.001732 | 0.013945 | 2.041791 |
| kiaa0895 | 5.649138 | 0.001736 | 0.013967 | 2.498031 |
| robo2 | 0.196353 | 0.001738 | 0.013967 | 2.348481 |
| gpt | 0.246395 | 0.001737 | 0.013967 | 2.020953 |
| mcm7 | 0.195564 | 0.001748 | 0.013992 | 2.354288 |
| LOC103387225 | 10.38083 | 0.001753 | 0.013995 | 3.37585 |
| LOC103377326 | 5.482776 | 0.001753 | 0.013995 | 2.454907 |
| mrpl17 | 0.142478 | 0.001765 | 0.014052 | 2.81119 |
| LOC103382433 | 0.187498 | 0.001769 | 0.014071 | 2.415055 |
| pax1 | 37.62034 | 0.001774 | 0.01408 | 5.233441 |
| LOC103384243 | 6.267555 | 0.001775 | 0.01408 | 2.647903 |
| LOC103379834 | 0.175333 | 0.001773 | 0.01408 | 2.511828 |
| LOC103378914 | 0.17777 | 0.001778 | 0.014102 | 2.491915 |
| LOC103392904 | 16.70944 | 0.00178 | 0.014103 | 4.062591 |
| LOC103383650 | 0.198868 | 0.001783 | 0.014112 | 2.33012 |
| lypla2 | 4.616179 | 0.001783 | 0.014112 | 2.206699 |
| LOC103378493 | 4.110036 | 0.001796 | 0.014169 | 2.039151 |
| mettl18 | 0.229348 | 0.001802 | 0.014191 | 2.124391 |
| LOC103390710 | 0.135002 | 0.001809 | 0.014223 | 2.88895 |
| sh3yl1 | 0.248708 | 0.001809 | 0.014223 | 2.007477 |
| LOC112486492 | 0.151596 | 0.00182 | 0.014274 | 2.721701 |
| ccnb1 | 0.072332 | 0.001823 | 0.014279 | 3.789222 |
| LOC103396021 | 8.334058 | 0.001823 | 0.014279 | 3.059019 |
| LOC103390813 | 5.77919 | 0.001846 | 0.014402 | 2.530867 |
| LOC103393472 | 5.755759 | 0.001852 | 0.014411 | 2.525006 |
| haus3 | 0.16505 | 0.001854 | 0.014417 | 2.599021 |
| LOC103387334 | 0.246671 | 0.001867 | 0.014456 | 2.019342 |
| ube2j1 | 4.154102 | 0.001874 | 0.014475 | 2.054537 |
| thap4 | 0.17061 | 0.001883 | 0.014505 | 2.551224 |
| LOC103380739 | 4.005881 | 0.001888 | 0.014521 | 2.00212 |
| mkrn1 | 5.746823 | 0.001895 | 0.014552 | 2.522765 |
| LOC103389644 | 5.670988 | 0.001902 | 0.014585 | 2.5036 |
| LOC103399976 | 0.24943 | 0.001907 | 0.014611 | 2.003292 |
| trmt13 | 0.221343 | 0.00191 | 0.014626 | 2.175645 |
| LOC103396541 | 5.827279 | 0.001916 | 0.014659 | 2.542822 |
| LOC103382283 | 4.1948 | 0.001919 | 0.014666 | 2.068602 |
| slc25a10 | 0.183026 | 0.001921 | 0.014675 | 2.449876 |
| LOC103397956 | 0.216941 | 0.001926 | 0.014696 | 2.204627 |
| LOC103398060 | 0.231067 | 0.001929 | 0.014698 | 2.113619 |
| LOC103381481 | 0.220114 | 0.001934 | 0.014725 | 2.183676 |
| LOC103396370 | 0.16402 | 0.00194 | 0.014744 | 2.608057 |
| rnls | 0.143141 | 0.001949 | 0.014793 | 2.804487 |
| LOC103396414 | 4.995032 | 0.00195 | 0.014795 | 2.320494 |
| LOC112488045 | 0.218057 | 0.001955 | 0.014824 | 2.197223 |
| LOC103388334 | 0.159227 | 0.001957 | 0.014827 | 2.650842 |
| LOC103396137 | 4.873308 | 0.001957 | 0.014827 | 2.284901 |
| LOC103393907 | 0.125945 | 0.001969 | 0.014879 | 2.989137 |
| fsd2 | 4.224109 | 0.001969 | 0.014879 | 2.078647 |
| lyg | 4.341035 | 0.001976 | 0.014891 | 2.118039 |
| LOC103393908 | 0.192084 | 0.001981 | 0.014905 | 2.380188 |
| rexo5 | 0.225997 | 0.001986 | 0.014918 | 2.145622 |
| LOC103388422 | 0.22587 | 0.001994 | 0.014959 | 2.146436 |
| LOC103395985 | 4.559277 | 0.002006 | 0.015022 | 2.188805 |
| LOC103392347 | 0.115352 | 0.002008 | 0.015027 | 3.11588 |
| LOC103388821 | 4.210913 | 0.002011 | 0.015043 | 2.074133 |
| vldlr | 0.033656 | 0.002022 | 0.015077 | 4.892994 |
| LOC103394738 | 6.74629 | 0.002022 | 0.015077 | 2.754094 |
| trnal-uag | 5.808322 | 0.00202 | 0.015077 | 2.538121 |
| LOC107989470 | 4.17938 | 0.002032 | 0.015103 | 2.063289 |
| LOC103390313 | 4.609698 | 0.002033 | 0.015105 | 2.204672 |
| epg5 | 4.301642 | 0.002039 | 0.015121 | 2.104887 |
| LOC103385550 | 8.757362 | 0.002054 | 0.015185 | 3.130496 |
| LOC103399292 | 24.40766 | 0.002061 | 0.015188 | 4.609262 |
| LOC103383572 | 12.32009 | 0.00206 | 0.015188 | 3.622941 |
| LOC103393243 | 0.199308 | 0.002061 | 0.015188 | 2.326931 |
| LOC103388381 | 0.225888 | 0.002059 | 0.015188 | 2.146322 |
| LOC103384160 | 0.235117 | 0.002056 | 0.015188 | 2.08855 |
| grwd1 | 0.202358 | 0.002066 | 0.015196 | 2.30502 |
| LOC103395452 | 19.62568 | 0.002092 | 0.015321 | 4.294671 |
| nr0b1 | 0.141116 | 0.0021 | 0.015357 | 2.825046 |
| ulk1 | 5.901349 | 0.002107 | 0.015372 | 2.561045 |
| LOC112488053 | 0.244344 | 0.002109 | 0.015372 | 2.033014 |
| elmod1 | 0.202011 | 0.002117 | 0.015392 | 2.307496 |
| armc8 | 4.440067 | 0.002116 | 0.015392 | 2.150581 |
| ppm1k | 4.450878 | 0.002134 | 0.015469 | 2.15409 |
| LOC103381863 | 6.144991 | 0.002145 | 0.015512 | 2.619411 |
| LOC103381863 | 6.144991 | 0.002145 | 0.015512 | 2.619411 |
| LOC103381863 | 6.144991 | 0.002145 | 0.015512 | 2.619411 |
| LOC103381863 | 6.144991 | 0.002145 | 0.015512 | 2.619411 |
| LOC103381863 | 6.144991 | 0.002145 | 0.015512 | 2.619411 |
| LOC103381863 | 6.144991 | 0.002145 | 0.015512 | 2.619411 |
| LOC103381863 | 6.144991 | 0.002145 | 0.015512 | 2.619411 |
| LOC103381863 | 6.144991 | 0.002145 | 0.015512 | 2.619411 |
| LOC103381863 | 6.144991 | 0.002145 | 0.015512 | 2.619411 |
| LOC103381863 | 6.144991 | 0.002145 | 0.015512 | 2.619411 |
| LOC103381863 | 6.144991 | 0.002145 | 0.015512 | 2.619411 |
| LOC103381863 | 6.144991 | 0.002145 | 0.015512 | 2.619411 |
| LOC103381863 | 6.144991 | 0.002145 | 0.015512 | 2.619411 |
| LOC103381863 | 6.144991 | 0.002145 | 0.015512 | 2.619411 |
| LOC103381863 | 6.144991 | 0.002145 | 0.015512 | 2.619411 |
| LOC103381863 | 6.144991 | 0.002145 | 0.015512 | 2.619411 |
| LOC103389933 | 4.023196 | 0.002144 | 0.015512 | 2.008342 |
| LOC103389106 | 0.142523 | 0.002147 | 0.015515 | 2.810732 |
| LOC103379819 | 0.208149 | 0.002151 | 0.015527 | 2.264309 |
| srpx2 | 5.578447 | 0.002156 | 0.015547 | 2.479863 |
| zfp36l1 | 0.220675 | 0.002159 | 0.01555 | 2.180006 |
| LOC103388005 | 12.61394 | 0.002169 | 0.015601 | 3.656947 |
| LOC103378164 | 0.044961 | 0.002172 | 0.015607 | 4.475167 |
| gskip | 4.479913 | 0.002174 | 0.015616 | 2.163471 |
| dmrta2 | 4.078641 | 0.002177 | 0.015631 | 2.028088 |
| pdia4 | 0.211751 | 0.002184 | 0.015654 | 2.239562 |
| eno4 | 8.180083 | 0.002189 | 0.015669 | 3.032115 |
| LOC103390697 | 5.197794 | 0.002188 | 0.015669 | 2.377899 |
| LOC107989436 | 6.510308 | 0.002195 | 0.015685 | 2.702726 |
| camk2a | 5.198243 | 0.002198 | 0.015689 | 2.378024 |
| ccna2 | 0.102171 | 0.002209 | 0.015736 | 3.290941 |
| LOC103382179 | 4.559412 | 0.002216 | 0.01577 | 2.188848 |
| trnah-gug | 4.339783 | 0.002221 | 0.015776 | 2.117623 |
| LOC103386286 | 0.162281 | 0.002227 | 0.01578 | 2.623432 |
| LOC103379536 | 12.11142 | 0.002238 | 0.015845 | 3.598296 |
| elovl7 | 0.135178 | 0.002252 | 0.015911 | 2.887067 |
| LOC103392755 | 0.117315 | 0.002264 | 0.015955 | 3.091543 |
| trnap-ugg | 4.538904 | 0.002274 | 0.015985 | 2.182344 |
| LOC103384841 | 0.242587 | 0.002276 | 0.015993 | 2.043424 |
| ppp1r3c | 0.205472 | 0.002281 | 0.016009 | 2.282985 |
| upp2 | 5.109064 | 0.002297 | 0.016096 | 2.353059 |
| LOC103385014 | 34.08439 | 0.002302 | 0.01611 | 5.091039 |
| ptpn12 | 0.208458 | 0.002306 | 0.016128 | 2.262171 |
| fgfr3 | 0.11025 | 0.002314 | 0.016173 | 3.181149 |
| rreb1 | 4.935019 | 0.002326 | 0.016212 | 2.303056 |
| rwdd4 | 0.233564 | 0.002325 | 0.016212 | 2.098113 |
| atad3a | 0.218676 | 0.002329 | 0.016222 | 2.193132 |
| LOC103395722 | 0.225756 | 0.002348 | 0.016307 | 2.147164 |
| LOC103394588 | 0.230675 | 0.002348 | 0.016307 | 2.116066 |
| LOC112487491 | 0.155161 | 0.002351 | 0.016321 | 2.688164 |
| manba | 0.216068 | 0.00236 | 0.01634 | 2.210444 |
| LOC103388822 | 0.239242 | 0.002359 | 0.01634 | 2.063459 |
| rab38 | 0.237597 | 0.002364 | 0.016346 | 2.073413 |
| mrpl12 | 0.248503 | 0.002371 | 0.016369 | 2.008665 |
| LOC112487024 | 4.673202 | 0.002373 | 0.016374 | 2.224411 |
| LOC103396839 | 12.96525 | 0.002398 | 0.016478 | 3.696578 |
| LOC103388738 | 5.737971 | 0.00242 | 0.016577 | 2.520541 |
| LOC103384557 | 5.427983 | 0.00244 | 0.01664 | 2.440416 |
| clmn | 9.040904 | 0.002456 | 0.016722 | 3.176467 |
| LOC107989488 | 6.036773 | 0.002455 | 0.016722 | 2.593778 |
| LOC103385459 | 0.153041 | 0.002459 | 0.016736 | 2.708008 |
| rngtt | 4.051372 | 0.002463 | 0.01674 | 2.01841 |
| b9d2 | 4.392523 | 0.002472 | 0.016761 | 2.13505 |
| LOC103392217 | 5.960656 | 0.002481 | 0.016797 | 2.575471 |
| LOC103389597 | 0.08456 | 0.002485 | 0.016809 | 3.563875 |
| c5h15orf48 | 11.12228 | 0.002501 | 0.016884 | 3.47538 |
| lacc1 | 0.196978 | 0.002505 | 0.016903 | 2.343894 |
| LOC103381292 | 6.477625 | 0.002512 | 0.016914 | 2.695465 |
| paics | 0.124549 | 0.00252 | 0.016944 | 3.005217 |
| mylip | 0.20166 | 0.00252 | 0.016944 | 2.31 |
| LOC103396561 | 4.007383 | 0.002519 | 0.016944 | 2.002661 |
| LOC103382798 | 8.599298 | 0.002523 | 0.016949 | 3.104219 |
| orc5 | 0.237822 | 0.002522 | 0.016949 | 2.072049 |
| ankrd45 | 5.54176 | 0.002528 | 0.016969 | 2.470344 |
| fam135b | 5.583558 | 0.002536 | 0.016985 | 2.481185 |
| mllt11 | 12.37835 | 0.002554 | 0.017012 | 3.629747 |
| LOC103392579 | 0.235004 | 0.002553 | 0.017012 | 2.089245 |
| LOC103397615 | 7.877599 | 0.00256 | 0.017017 | 2.977756 |
| LOC103392833 | 0.039088 | 0.002568 | 0.017057 | 4.67713 |
| mtap | 0.248191 | 0.002612 | 0.017233 | 2.010476 |
| nelfe | 4.667002 | 0.002614 | 0.017238 | 2.222496 |
| LOC103396040 | 0.182085 | 0.002647 | 0.017384 | 2.457318 |
| LOC103381311 | 4.33436 | 0.002648 | 0.017384 | 2.115819 |
| LOC103382515 | 8.299742 | 0.00265 | 0.017387 | 3.053066 |
| ip6k1 | 9.143767 | 0.002656 | 0.017414 | 3.192789 |
| csrnp2 | 9.812787 | 0.002666 | 0.017465 | 3.294663 |
| cdc45 | 0.215416 | 0.002668 | 0.01747 | 2.214801 |
| trim33 | 4.868507 | 0.002672 | 0.017487 | 2.283479 |
| LOC103385495 | 0.158643 | 0.002675 | 0.017489 | 2.656145 |
| LOC103398951 | 5.069679 | 0.002677 | 0.017495 | 2.341894 |
| LOC103394134 | 5.812061 | 0.002703 | 0.017589 | 2.53905 |
| larp4b | 4.617194 | 0.002727 | 0.017692 | 2.207016 |
| lrp12 | 4.999855 | 0.002742 | 0.017752 | 2.321886 |
| LOC103398003 | 0.218501 | 0.002741 | 0.017752 | 2.19429 |
| LOC103396797 | 0.104221 | 0.002748 | 0.017775 | 3.262288 |
| LOC103392011 | 0.174102 | 0.002756 | 0.017805 | 2.521991 |
| LOC103378771 | 0.106596 | 0.00278 | 0.017921 | 3.229777 |
| LOC103391081 | 13.5417 | 0.002793 | 0.017966 | 3.759337 |
| LOC103389172 | 0.206221 | 0.002795 | 0.017968 | 2.27774 |
| LOC103391031 | 0.236624 | 0.002797 | 0.017976 | 2.079331 |
| oaf | 0.230846 | 0.002798 | 0.017977 | 2.114996 |
| gpam | 5.731357 | 0.002808 | 0.018017 | 2.518877 |
| LOC107989985 | 6.246708 | 0.00282 | 0.018033 | 2.643096 |
| LOC103392675 | 5.238121 | 0.002848 | 0.018151 | 2.389049 |
| LOC103383177 | 4.186659 | 0.002852 | 0.018157 | 2.065799 |
| znrf2 | 4.816239 | 0.002892 | 0.018298 | 2.267907 |
| LOC103379402 | 8.500388 | 0.002915 | 0.018388 | 3.087529 |
| c1ql3 | 0.147718 | 0.002921 | 0.018414 | 2.759084 |
| tmem126a | 5.042419 | 0.002921 | 0.018414 | 2.334116 |
| LOC103385310 | 5.63389 | 0.002934 | 0.018477 | 2.494131 |
| kif3c | 9.767444 | 0.002943 | 0.018498 | 3.287981 |
| LOC103379187 | 0.129597 | 0.002945 | 0.018498 | 2.947897 |
| nop14 | 0.247542 | 0.002944 | 0.018498 | 2.014254 |
| LOC103395379 | 6.267412 | 0.002964 | 0.018572 | 2.64787 |
| ankmy1 | 4.408196 | 0.002972 | 0.01859 | 2.140188 |
| fbxo15 | 10.46934 | 0.002987 | 0.018654 | 3.388099 |
| LOC103390759 | 0.215563 | 0.002989 | 0.018662 | 2.213818 |
| wnt9b | 0.139417 | 0.002996 | 0.018689 | 2.842519 |
| msto1 | 0.231587 | 0.003002 | 0.018716 | 2.110371 |
| LOC103378668 | 0.215751 | 0.003015 | 0.018768 | 2.212563 |
| LOC103396423 | 46.05653 | 0.003024 | 0.018782 | 5.525334 |
| dusp23 | 8.207374 | 0.003023 | 0.018782 | 3.036921 |
| prkag2 | 7.16711 | 0.003023 | 0.018782 | 2.841391 |
| trnad-guc | 5.542891 | 0.003024 | 0.018782 | 2.470639 |
| tmem53 | 0.245366 | 0.003024 | 0.018782 | 2.026992 |
| LOC103379080 | 0.225503 | 0.003041 | 0.018855 | 2.148778 |
| notum | 0.23493 | 0.003046 | 0.018855 | 2.089698 |
| LOC103393288 | 13.14818 | 0.003057 | 0.018901 | 3.716791 |
| LOC103393685 | 0.116924 | 0.003095 | 0.018997 | 3.096359 |
| LOC103377799 | 8.489525 | 0.003081 | 0.018997 | 3.085684 |
| LOC103384648 | 0.170418 | 0.003092 | 0.018997 | 2.552849 |
| LOC103395468 | 6.721335 | 0.003111 | 0.019054 | 2.748748 |
| LOC103381038 | 6.683053 | 0.003129 | 0.019135 | 2.740507 |
| LOC103397721 | 6.378572 | 0.003137 | 0.019159 | 2.673233 |
| LOC103396794 | 0.184371 | 0.003138 | 0.019161 | 2.439313 |
| LOC107988364 | 6.158153 | 0.003163 | 0.019258 | 2.622498 |
| LOC103390737 | 4.369831 | 0.003181 | 0.019284 | 2.127577 |
| baiap2 | 0.142148 | 0.003216 | 0.01945 | 2.814533 |
| LOC103379865 | 4.876475 | 0.003219 | 0.019464 | 2.285839 |
| LOC103385255 | 0.245622 | 0.003222 | 0.019467 | 2.025488 |
| ssh2 | 0.217875 | 0.003233 | 0.019505 | 2.19843 |
| LOC103387257 | 4.289399 | 0.003294 | 0.019754 | 2.100776 |
| LOC103392792 | 5.09776 | 0.003296 | 0.019755 | 2.349864 |
| LOC103394167 | 18.89585 | 0.003301 | 0.019775 | 4.239997 |
| LOC103395231 | 6.808874 | 0.003304 | 0.019779 | 2.767416 |
| LOC103399918 | 0.12666 | 0.003323 | 0.019837 | 2.980962 |
| LOC103399979 | 0.246061 | 0.003325 | 0.019837 | 2.022909 |
| ccdc62 | 5.023881 | 0.003331 | 0.019842 | 2.328802 |
| LOC103395356 | 0.23566 | 0.003342 | 0.019884 | 2.08522 |
| LOC103392282 | 4.221962 | 0.003369 | 0.019976 | 2.077914 |
| lrrc45 | 4.403676 | 0.003388 | 0.020046 | 2.138708 |
| LOC103393524 | 6.225428 | 0.003391 | 0.020059 | 2.638173 |
| gba | 0.201066 | 0.0034 | 0.020094 | 2.31426 |
| LOC103379137 | 4.749106 | 0.003407 | 0.020128 | 2.247656 |
| LOC112486259 | 10.41783 | 0.003418 | 0.020159 | 3.380983 |
| serpinc1 | 7.582342 | 0.003428 | 0.0202 | 2.922643 |
| LOC103388768 | 4.611573 | 0.003433 | 0.020222 | 2.205259 |
| LOC112487245 | 7.05531 | 0.003436 | 0.02023 | 2.818709 |
| LOC103396736 | 0.236559 | 0.003435 | 0.02023 | 2.079729 |
| dpys | 0.228824 | 0.003454 | 0.020299 | 2.127689 |
| LOC103395175 | 19.91093 | 0.003494 | 0.020433 | 4.315488 |
| LOC103388121 | 7.635751 | 0.003519 | 0.020518 | 2.93277 |
| nap1l1 | 0.203962 | 0.003544 | 0.020584 | 2.29363 |
| aldh6a1 | 0.156379 | 0.003566 | 0.020625 | 2.676883 |
| LOC103391927 | 11.29549 | 0.003571 | 0.020639 | 3.497674 |
| LOC103383498 | 0.184999 | 0.003576 | 0.020655 | 2.434407 |
| LOC103392014 | 6.435586 | 0.003628 | 0.020839 | 2.686072 |
| LOC112487034 | 8.557102 | 0.00363 | 0.020843 | 3.097122 |
| LOC103391270 | 4.149153 | 0.003635 | 0.020858 | 2.052817 |
| hnmt | 0.16561 | 0.003659 | 0.020948 | 2.59414 |
| prkcq | 4.228647 | 0.00366 | 0.020948 | 2.080196 |
| LOC103385527 | 4.192909 | 0.003666 | 0.020958 | 2.067952 |
| LOC103380327 | 5.848036 | 0.003684 | 0.02101 | 2.547952 |
| LOC103379523 | 0.184927 | 0.003732 | 0.021191 | 2.43497 |
| atf6 | 4.245969 | 0.003738 | 0.021212 | 2.086094 |
| mex3d | 5.695857 | 0.003761 | 0.02131 | 2.509913 |
| LOC103397552 | 43.4587 | 0.003779 | 0.021371 | 5.441573 |
| LOC103383828 | 0.132406 | 0.003822 | 0.021458 | 2.916965 |
| LOC103385944 | 4.331274 | 0.00382 | 0.021458 | 2.114792 |
| lgr5 | 7.37449 | 0.003844 | 0.021502 | 2.882543 |
| LOC103383714 | 5.287903 | 0.00384 | 0.021502 | 2.402696 |
| LOC103393518 | 0.219539 | 0.003841 | 0.021502 | 2.187451 |
| foxo3 | 5.235673 | 0.003845 | 0.021505 | 2.388375 |
| LOC103379934 | 5.394846 | 0.003908 | 0.021714 | 2.431582 |
| LOC103380163 | 0.199584 | 0.003916 | 0.021735 | 2.32493 |
| LOC107989508 | 18.79936 | 0.003939 | 0.021805 | 4.232611 |
| LOC103385127 | 0.205968 | 0.00394 | 0.021805 | 2.27951 |
| LOC107988478 | 4.837578 | 0.003941 | 0.021805 | 2.274285 |
| LOC103381200 | 0.182955 | 0.003965 | 0.02189 | 2.450437 |
| LOC103397790 | 0.197508 | 0.003986 | 0.021962 | 2.340014 |
| LOC112487023 | 0.231439 | 0.003986 | 0.021962 | 2.111299 |
| LOC103398336 | 4.684018 | 0.004006 | 0.022036 | 2.227747 |
| LOC103383794 | 0.218331 | 0.004012 | 0.022037 | 2.195412 |
| LOC103381129 | 4.184174 | 0.004115 | 0.022346 | 2.064943 |
| LOC103391779 | 7.08346 | 0.004121 | 0.022365 | 2.824454 |
| LOC103388314 | 0.176978 | 0.004122 | 0.022366 | 2.498355 |
| LOC112486249 | 0.193894 | 0.004164 | 0.022543 | 2.366659 |
| LOC103393652 | 0.107382 | 0.004217 | 0.022724 | 3.219171 |
| LOC103383589 | 0.131567 | 0.004226 | 0.022732 | 2.92613 |
| dtx3 | 10.40472 | 0.004246 | 0.02279 | 3.379166 |
| LOC103384892 | 4.576982 | 0.004269 | 0.022839 | 2.194397 |
| LOC103378548 | 6.616642 | 0.00431 | 0.022985 | 2.726099 |
| hspd1 | 0.165845 | 0.004309 | 0.022985 | 2.592094 |
| sytl3 | 4.965839 | 0.004318 | 0.023019 | 2.312037 |
| LOC107990221 | 4.419586 | 0.004318 | 0.023019 | 2.143911 |
| LOC103394982 | 7.638204 | 0.004344 | 0.023101 | 2.933234 |
| LOC103394976 | 43.62392 | 0.00436 | 0.02316 | 5.447047 |
| LOC103383797 | 0.24198 | 0.004368 | 0.023192 | 2.04704 |
| LOC103381247 | 0.17423 | 0.004388 | 0.023255 | 2.520933 |
| LOC103381007 | 8.957307 | 0.004443 | 0.02341 | 3.163065 |
| LOC103382736 | 8.937162 | 0.004507 | 0.023652 | 3.159817 |
| prkar2a | 5.08039 | 0.004517 | 0.023675 | 2.344939 |
| kif5b | 4.049708 | 0.004519 | 0.023679 | 2.017818 |
| LOC112488408 | 4.55014 | 0.00454 | 0.02375 | 2.185911 |
| LOC103395632 | 4.044352 | 0.004571 | 0.023839 | 2.015909 |
| LOC103389949 | 0.165531 | 0.004586 | 0.023884 | 2.594823 |
| LOC103381848 | 4.944381 | 0.004588 | 0.023892 | 2.30579 |
| LOC103376849 | 54.71514 | 0.0046 | 0.023932 | 5.773868 |
| flt3 | 4.671286 | 0.00461 | 0.023951 | 2.22382 |
| LOC103386118 | 0.211784 | 0.004628 | 0.024002 | 2.239333 |
| LOC103396768 | 23.33794 | 0.004634 | 0.024015 | 4.544605 |
| arhgdia | 0.244438 | 0.004654 | 0.024073 | 2.032457 |
| LOC103381213 | 0.232173 | 0.004657 | 0.024082 | 2.106726 |
| anos1 | 0.226714 | 0.004663 | 0.02409 | 2.141054 |
| LOC103386869 | 40.89144 | 0.004676 | 0.024114 | 5.353727 |
| myrf | 0.196268 | 0.004749 | 0.024389 | 2.349107 |
| LOC112488320 | 0.237158 | 0.004777 | 0.024474 | 2.076082 |
| LOC103383042 | 11.00057 | 0.004796 | 0.024555 | 3.459506 |
| klhl11 | 4.484724 | 0.004844 | 0.024664 | 2.165019 |
| ahcyl1 | 5.846997 | 0.004851 | 0.024688 | 2.547696 |
| LOC103376967 | 0.212364 | 0.004859 | 0.024716 | 2.235392 |
| LOC103389309 | 0.214349 | 0.004905 | 0.024873 | 2.221966 |
| lin28b | 0.242071 | 0.004914 | 0.024912 | 2.046497 |
| LOC103390504 | 6.574849 | 0.004935 | 0.024974 | 2.716958 |
| LOC103389357 | 4.264727 | 0.004996 | 0.025149 | 2.092453 |
| LOC103396142 | 21.81879 | 0.005001 | 0.025155 | 4.447499 |
| elf4 | 8.005552 | 0.005 | 0.025155 | 3.001001 |
| inka1 | 0.086603 | 0.005006 | 0.025165 | 3.529442 |
| LOC103393662 | 0.196416 | 0.005033 | 0.025239 | 2.348019 |
| LOC103398121 | 0.216073 | 0.005047 | 0.025273 | 2.210408 |
| LOC112487970 | 0.212062 | 0.005059 | 0.025299 | 2.237444 |
| mettl26 | 0.193583 | 0.005089 | 0.025388 | 2.368979 |
| sox4 | 0.208912 | 0.005121 | 0.025465 | 2.259032 |
| sfi1 | 5.658046 | 0.005126 | 0.025483 | 2.500304 |
| shcbp1 | 0.208056 | 0.005149 | 0.025549 | 2.264956 |
| LOC103383404 | 0.07614 | 0.005164 | 0.025582 | 3.7152 |
| nxn | 0.249891 | 0.005182 | 0.02565 | 2.00063 |
| LOC103380838 | 4.058142 | 0.005187 | 0.025665 | 2.020819 |
| LOC103380775 | 0.127235 | 0.00523 | 0.025832 | 2.974431 |
| efnb2 | 6.876677 | 0.005236 | 0.025851 | 2.781712 |
| LOC107990037 | 4.316766 | 0.005249 | 0.025897 | 2.109951 |
| ascl1 | 4.662421 | 0.00527 | 0.025964 | 2.221079 |
| LOC103388311 | 4.823076 | 0.005284 | 0.026006 | 2.269954 |
| ccdc78 | 0.232792 | 0.005299 | 0.026044 | 2.102886 |
| rassf4 | 4.933152 | 0.005317 | 0.026101 | 2.30251 |
| creg1 | 0.243815 | 0.005319 | 0.026103 | 2.03614 |
| trnac-gca | 5.845876 | 0.00535 | 0.026215 | 2.547419 |
| ccdc148 | 6.714355 | 0.005366 | 0.026277 | 2.747249 |
| LOC103396670 | 4.541427 | 0.005408 | 0.026417 | 2.183146 |
| LOC103391210 | 6.159995 | 0.005464 | 0.026527 | 2.622929 |
| LOC103388537 | 4.042302 | 0.005496 | 0.026615 | 2.015177 |
| LOC103382911 | 0.20007 | 0.0055 | 0.026621 | 2.321424 |
| trnag-ccc | 5.535477 | 0.005508 | 0.026646 | 2.468708 |
| abcc5 | 4.751428 | 0.005508 | 0.026646 | 2.248361 |
| gins3 | 0.230814 | 0.005533 | 0.026735 | 2.115198 |
| eml2 | 0.180448 | 0.00558 | 0.026885 | 2.470344 |
| LOC103381695 | 6.512059 | 0.005635 | 0.027031 | 2.703114 |
| LOC103391023 | 0.213881 | 0.005658 | 0.027061 | 2.225117 |
| LOC103388957 | 4.881726 | 0.005748 | 0.027358 | 2.287391 |
| LOC103389086 | 5.318168 | 0.005816 | 0.027576 | 2.410929 |
| LOC103377407 | 4.638097 | 0.005925 | 0.027896 | 2.213533 |
| LOC103389665 | 0.232286 | 0.005938 | 0.027926 | 2.106029 |
| LOC103377702 | 5.12095 | 0.005964 | 0.028005 | 2.356411 |
| LOC103381312 | 4.512557 | 0.006 | 0.028121 | 2.173945 |
| LOC103395421 | 4.380358 | 0.00601 | 0.028154 | 2.131049 |
| LOC103379779 | 4.898626 | 0.006026 | 0.028212 | 2.292377 |
| LOC103385686 | 0.230387 | 0.006125 | 0.028497 | 2.117868 |
| LOC103391546 | 4.049941 | 0.006141 | 0.028561 | 2.017901 |
| utp25 | 0.242276 | 0.006161 | 0.028598 | 2.045276 |
| LOC103391500 | 4.047839 | 0.006219 | 0.028744 | 2.017152 |
| LOC103387713 | 4.602503 | 0.006323 | 0.029026 | 2.202419 |
| LOC103390645 | 0.116351 | 0.00636 | 0.029141 | 3.103449 |
| c4h3orf80 | 4.790273 | 0.006386 | 0.029209 | 2.260108 |
| LOC103391129 | 4.580797 | 0.006405 | 0.02926 | 2.195599 |
| cfap74 | 4.11664 | 0.006555 | 0.029677 | 2.041467 |
| serpinf2 | 4.504718 | 0.006577 | 0.029763 | 2.171437 |
| LOC112487726 | 0.170064 | 0.006596 | 0.02983 | 2.555847 |
| LOC103377320 | 7.872504 | 0.006618 | 0.029876 | 2.976823 |
| LOC112488544 | 9.520227 | 0.006628 | 0.029898 | 3.250996 |
| LOC103396000 | 0.213579 | 0.006631 | 0.029905 | 2.227159 |
| LOC103376687 | 6.442373 | 0.006695 | 0.030061 | 2.687592 |
| LOC103383200 | 4.790591 | 0.006802 | 0.030406 | 2.260204 |
| LOC103397356 | 8.313853 | 0.006815 | 0.030455 | 3.055517 |
| LOC103380492 | 4.377156 | 0.006844 | 0.030521 | 2.129994 |
| sema5a | 0.168345 | 0.006931 | 0.030736 | 2.57051 |
| ahcy | 0.199946 | 0.007014 | 0.030983 | 2.322319 |
| LOC103384877 | 0.139123 | 0.007019 | 0.030994 | 2.845567 |
| LOC103394990 | 4.847738 | 0.007024 | 0.030996 | 2.277312 |
| LOC103383309 | 4.630114 | 0.007055 | 0.031079 | 2.211048 |
| LOC103395685 | 4.615887 | 0.00706 | 0.031089 | 2.206608 |
| LOC103385982 | 4.11569 | 0.007094 | 0.031182 | 2.041134 |
| LOC103383704 | 7.015417 | 0.007182 | 0.03143 | 2.810529 |
| LOC103393971 | 4.488396 | 0.007206 | 0.031496 | 2.1662 |
| pfas | 0.198592 | 0.007234 | 0.031581 | 2.33212 |
| LOC103378952 | 8.22535 | 0.007295 | 0.031689 | 3.040077 |
| myt1 | 4.513317 | 0.00731 | 0.031728 | 2.174188 |
| ublcp1 | 4.391425 | 0.007319 | 0.03176 | 2.134689 |
| LOC103381022 | 0.220641 | 0.007334 | 0.031812 | 2.180228 |
| LOC107990027 | 4.186838 | 0.007366 | 0.031889 | 2.065861 |
| LOC103394208 | 7.233193 | 0.007452 | 0.03209 | 2.854633 |
| LOC103396790 | 5.316172 | 0.007517 | 0.03225 | 2.410388 |
| LOC103390023 | 4.448453 | 0.007525 | 0.032274 | 2.153304 |
| LOC103393122 | 7.935778 | 0.00757 | 0.032421 | 2.988372 |
| pygl | 0.191763 | 0.00769 | 0.032785 | 2.382603 |
| LOC103391293 | 6.717408 | 0.007692 | 0.032789 | 2.747905 |
| hspa4l | 4.031375 | 0.007706 | 0.032813 | 2.011272 |
| LOC112487631 | 6.793971 | 0.007754 | 0.032879 | 2.764255 |
| LOC107988781 | 4.491831 | 0.00774 | 0.032879 | 2.167304 |
| LOC103377896 | 10.63777 | 0.0078 | 0.032973 | 3.411124 |
| LOC103392695 | 0.163555 | 0.007812 | 0.033017 | 2.612157 |
| gmnn | 0.15835 | 0.007819 | 0.033032 | 2.658807 |
| rrm2 | 0.191145 | 0.007882 | 0.033191 | 2.387261 |
| LOC103393706 | 0.163025 | 0.007994 | 0.033472 | 2.616832 |
| LOC103380461 | 0.062227 | 0.008031 | 0.033534 | 4.006324 |
| LOC103381561 | 0.165021 | 0.008045 | 0.033563 | 2.599277 |
| ckb | 0.059033 | 0.008102 | 0.033746 | 4.082327 |
| gtse1 | 0.2096 | 0.008104 | 0.033749 | 2.254287 |
| prr18 | 0.083072 | 0.008115 | 0.033766 | 3.5895 |
| LOC103396454 | 4.803159 | 0.008123 | 0.033789 | 2.263984 |
| LOC103383082 | 4.778577 | 0.008163 | 0.033894 | 2.256581 |
| LOC103395689 | 4.147834 | 0.008258 | 0.03419 | 2.052358 |
| fat1 | 0.238551 | 0.008303 | 0.034297 | 2.067628 |
| wnt16 | 4.402142 | 0.008319 | 0.034338 | 2.138206 |
| LOC103386434 | 0.234992 | 0.008417 | 0.034559 | 2.089319 |
| LOC103389202 | 4.851856 | 0.008549 | 0.034931 | 2.278537 |
| LOC103395013 | 0.14773 | 0.008551 | 0.034934 | 2.758968 |
| LOC103390999 | 0.248192 | 0.008575 | 0.035003 | 2.010473 |
| tmem41b | 4.041438 | 0.008586 | 0.035039 | 2.014869 |
| fermt1 | 0.227306 | 0.008633 | 0.035154 | 2.137293 |
| LOC103398128 | 0.167841 | 0.008655 | 0.035209 | 2.574832 |
| trnai-uau | 5.758768 | 0.008817 | 0.035692 | 2.52576 |
| LOC103387463 | 0.163058 | 0.008822 | 0.0357 | 2.616546 |
| LOC107989257 | 34.13363 | 0.008979 | 0.036101 | 5.093122 |
| LOC103385015 | 40.34783 | 0.009004 | 0.036162 | 5.334419 |
| LOC107988714 | 9.217414 | 0.009071 | 0.036305 | 3.204362 |
| LOC103389380 | 0.179385 | 0.009113 | 0.036444 | 2.478865 |
| LOC103391331 | 9.427963 | 0.009154 | 0.036509 | 3.236946 |
| sigmar1 | 0.156339 | 0.009163 | 0.036527 | 2.677248 |
| LOC103396247 | 0.230635 | 0.009237 | 0.036681 | 2.116316 |
| LOC103392198 | 0.18718 | 0.009252 | 0.036724 | 2.417503 |
| LOC103382513 | 6.807783 | 0.009342 | 0.03699 | 2.767185 |
| LOC103397020 | 5.182137 | 0.009457 | 0.037276 | 2.373547 |
| fam161b | 4.859133 | 0.009517 | 0.037454 | 2.280699 |
| LOC103394224 | 4.049027 | 0.009534 | 0.037493 | 2.017575 |
| LOC107990072 | 11.71451 | 0.0096 | 0.037655 | 3.550225 |
| LOC107989201 | 0.201693 | 0.009683 | 0.037852 | 2.309764 |
| LOC103396356 | 0.237293 | 0.009721 | 0.037922 | 2.075256 |
| LOC103396079 | 0.246549 | 0.009748 | 0.037985 | 2.020055 |
| LOC103386741 | 0.2368 | 0.009973 | 0.038538 | 2.078256 |
| LOC103382188 | 0.232979 | 0.010078 | 0.038759 | 2.10173 |
| LOC103393298 | 4.171707 | 0.010118 | 0.038823 | 2.060638 |
| nupr1 | 6.497313 | 0.01017 | 0.038929 | 2.699843 |
| gpc2 | 4.664005 | 0.010238 | 0.03909 | 2.221569 |
| pdgfrl | 5.378929 | 0.010258 | 0.039161 | 2.427319 |
| LOC112487559 | 6.435511 | 0.010398 | 0.039417 | 2.686055 |
| LOC103398324 | 0.244498 | 0.010586 | 0.039868 | 2.032105 |
| rasl11b | 5.317499 | 0.010599 | 0.039882 | 2.410748 |
| LOC103390007 | 0.144156 | 0.010684 | 0.040112 | 2.794293 |
| LOC107990069 | 6.303744 | 0.010739 | 0.04024 | 2.656209 |
| prkaa2 | 4.125734 | 0.010799 | 0.040415 | 2.044651 |
| LOC103388283 | 4.255635 | 0.01083 | 0.040502 | 2.089374 |
| LOC103398010 | 4.387153 | 0.011049 | 0.041045 | 2.133285 |
| LOC103396574 | 0.245079 | 0.011052 | 0.041045 | 2.028679 |
| LOC103388794 | 6.959363 | 0.011174 | 0.041319 | 2.798955 |
| trnar-ucu | 10.24489 | 0.011264 | 0.041542 | 3.356833 |
| ap4s1 | 4.013146 | 0.011441 | 0.04199 | 2.004733 |
| adam23 | 4.678518 | 0.011457 | 0.042023 | 2.226051 |
| LOC103387069 | 0.17355 | 0.011555 | 0.042234 | 2.526574 |
| LOC103377036 | 34.84815 | 0.011612 | 0.042375 | 5.12301 |
| LOC103385375 | 6.529853 | 0.011698 | 0.042554 | 2.70705 |
| LOC103380581 | 6.004264 | 0.01201 | 0.043297 | 2.585987 |
| fkbp14 | 4.265854 | 0.01206 | 0.043411 | 2.092834 |
| LOC103380425 | 0.15643 | 0.012196 | 0.043712 | 2.676408 |
| LOC112487518 | 0.170144 | 0.012241 | 0.043819 | 2.555169 |
| fam69b | 4.637554 | 0.012272 | 0.043899 | 2.213364 |
| cdadc1 | 4.312001 | 0.012353 | 0.044105 | 2.108357 |
| LOC103383281 | 0.095741 | 0.012439 | 0.044328 | 3.384716 |
| LOC103391287 | 0.119762 | 0.012538 | 0.044588 | 3.061764 |
| cdh15 | 8.120943 | 0.012622 | 0.044781 | 3.021647 |
| orc1 | 0.248391 | 0.012895 | 0.04546 | 2.009315 |
| il4i1 | 4.36709 | 0.013042 | 0.045802 | 2.126672 |
| LOC103384094 | 0.115448 | 0.0131 | 0.04592 | 3.114686 |
| nr2c2ap | 0.245128 | 0.01315 | 0.046045 | 2.028394 |
| ngb | 4.085067 | 0.013598 | 0.047094 | 2.03036 |
| serpini1 | 7.859291 | 0.013824 | 0.047602 | 2.974399 |
| trnan-guu | 4.073512 | 0.013915 | 0.047783 | 2.026273 |
| zar1l | 0.185583 | 0.013953 | 0.047846 | 2.429867 |
| deptor | 4.092312 | 0.014071 | 0.048127 | 2.032916 |
| bmp4 | 0.247735 | 0.014265 | 0.048509 | 2.013128 |
| olig3 | 5.19682 | 0.014663 | 0.049387 | 2.377629 |
| epcam | 0.151851 | 0.014922 | 0.049974 | 2.719268 |
