# Supplementary material for: Integrative analysis reveals pathways associated with sex reversal in Cynoglossus semilaevis
Source: PeerJ. 2020 Mar 19;8:e8801. doi: 10.7717/peerj.8801 (PMC7085895; doi:10.7717/peerj.8801)
Supplement: Table S2 [file peerj-08-8801-s004.docx]

**Supplement Table S2. List of all differentially expressed proteins.**

| Accession | FC | Pvalue |
| --- | --- | --- |
| A0A1A7W7W4 | 0.194661 | 3.38E-10 |
| H2LPK6 | 0.220151 | 8.33E-10 |
| G3N9L2 | 0.221506 | 2.44E-07 |
| A0A0F8C3K6 | 0.19047 | 1.28E-09 |
| A0A0F8CX77 | 0.279335 | 1.66E-05 |
| A0A087YKL5 | 0.296357 | 2.41E-07 |
| M3ZPQ1 | 0.155901 | 6.83E-07 |
| B7SVK2 | 4.77942 | 9.59E-05 |
| H2ULB2 | 0.255881 | 1.31E-07 |
| G8G8Y1 | 0.586731 | 6.32E-09 |
| A0A0F8CCG0 | 0.47078 | 1.09E-05 |
| I3IZU1 | 0.351785 | 9.67E-09 |
| A0A087XA45 | 0.601987 | 2.03E-09 |
| Q76CT4 | 0.450033 | 1.04E-07 |
| H2N320 | 1.663458 | 7.13E-07 |
| B1B560 | 0.566743 | 2.99E-09 |
| Q8JIM7 | 0.58501 | 1.78E-08 |
| H2U6I1 | 1.518759 | 9.03E-08 |
| C0SW46 | 0.393926 | 3.16E-09 |
| H2LN74 | 1.596691 | 6.84E-10 |
| M4AP69 | 0.623635 | 8.67E-10 |
| Q4SJW2 | 1.727506 | 2.88E-08 |
| A0A1A8D0E5 | 1.578576 | 8.37E-08 |
| A0A1A8AGB9 | 1.593099 | 3.99E-08 |
| A0A146NUM7 | 0.318108 | 4.12E-11 |
| A0A0F8AXL8 | 0.587958 | 3.16E-11 |
| A0A0S7G029 | 1.586569 | 3.59E-06 |
| T2B507 | 1.511119 | 1.76E-05 |
| A0A0N9H780 | 1.668067 | 9.48E-09 |
| S4T781 | 1.596241 | 6.5E-06 |
| H2MBE4 | 0.528535 | 3.72E-06 |
| I3JU24 | 0.400618 | 0.000539 |
| E2JF88 | 0.433817 | 1.5E-08 |
| A0A0F8AQN4 | 0.269299 | 1.03E-09 |
| A0A1A7XB59 | 1.562456 | 2.31E-07 |
| A0ELV8 | 1.683227 | 0.0002 |
| H2MFN0 | 1.59754 | 6.8E-05 |
| A0A087X562 | 1.708198 | 2.61E-08 |
| H3DIE5 | 0.342711 | 1.26E-08 |
| R9WN33 | 1.715331 | 1.38E-05 |
| A0A0S7JM13 | 1.795516 | 5.62E-08 |
| Q4S3P2 | 1.543702 | 1.52E-06 |
| H2MB55 | 1.958658 | 2.63E-08 |
| A0A1A8LH22 | 0.237872 | 1.71E-10 |
| C7EP36 | 0.285754 | 5.93E-05 |
| P83299 | 2.815634 | 1.83E-07 |
| A0A1A7XM15 | 1.628799 | 7.09E-08 |
| H2MCC8 | 1.507926 | 4.88E-07 |
| I3KEZ4 | 1.558431 | 4E-05 |
| A0A1A7YPI0 | 2.487828 | 7.65E-07 |
| A0A146NCY1 | 0.333194 | 4.53E-05 |
| A0A1A7WHV4 | 0.451939 | 3.19E-06 |
| H3CMB6 | 1.706727 | 8.01E-06 |
| G3P6S0 | 1.552 | 1.07E-08 |
| I3KEZ0 | 1.938909 | 4.68E-05 |
| H2U6U7 | 1.637355 | 0.003481 |
| H2TZ37 | 0.455847 | 8.45E-06 |
| A0A146MKB7 | 1.707093 | 1.29E-07 |
| E6ZH56 | 0.501133 | 1.03E-08 |
| Q8JG53 | 0.547525 | 4.16E-06 |
| A0A1A8CZC6 | 0.402698 | 0.005516 |
| A0A087XC31 | 1.623957 | 6.6E-06 |
| H2MMD3 | 1.564158 | 2.46E-05 |
| H2U937 | 1.710129 | 1.78E-06 |
| I3KGB1 | 2.754114 | 6.67E-07 |
| A0A146NN61 | 2.112827 | 5.37E-10 |
| G3Q2W6 | 0.517884 | 0.000126 |
| G3P7L3 | 0.648322 | 7.79E-09 |
| C3KHW5 | 0.366365 | 2.02E-06 |
| H2TGY6 | 0.570624 | 1.73E-05 |
| Q9DEA8 | 5.700387 | 1.33E-06 |
| H2SBN2 | 0.308571 | 3.72E-10 |
| A0A1A7XYL2 | 1.566004 | 0.000217 |
| H2T8N0 | 0.163361 | 4.87E-06 |
| I3J4Y9 | 1.891277 | 5.64E-09 |
| B9V3U1 | 0.325809 | 1.77E-09 |
| A0A087YMQ9 | 0.628994 | 4.78E-10 |
| H2UY95 | 1.507692 | 5.51E-06 |
| H2TT71 | 0.544207 | 1.07E-06 |
| D9I8D0 | 2.261938 | 1.69E-06 |
| A0A1A8EVF9 | 0.478185 | 7.76E-05 |
| U3M7T0 | 0.611441 | 1.27E-07 |
| K0A0D5 | 0.337564 | 1.33E-06 |
| B6VC67 | 0.549139 | 6.28E-07 |
| A0A096LVF7 | 0.628071 | 7.12E-07 |
| A0A146NFI9 | 1.51603 | 8.54E-06 |
| A0A0S7LN17 | 2.062273 | 5.03E-10 |
| G3NYU5 | 1.559971 | 1.24E-08 |
| H6UPM1 | 0.21052 | 1.75E-05 |
| I3JMP3 | 0.502579 | 5.93E-05 |
| S4TLM3 | 0.592371 | 3.42E-06 |
| A0A1A8GXF4 | 1.507136 | 4E-08 |
| A0A1B0SZU7 | 2.257256 | 8.27E-09 |
| Q4SP49 | 2.072874 | 8.7E-05 |
| A5JV29 | 0.620965 | 5.99E-09 |
| H2M098 | 0.493176 | 0.003007 |
| X2JGB1 | 4.254682 | 2.04E-10 |
| H2MU32 | 0.636783 | 9.6E-07 |
| I3KRX0 | 0.312543 | 2.73E-06 |
| G3NGQ4 | 0.666417 | 1.85E-05 |
| G3PET4 | 1.582774 | 7.52E-08 |
| A0A1A8FSV6 | 1.530793 | 4.21E-09 |
| B7U3X3 | 0.209787 | 2.68E-10 |
| A0A0S7F6C7 | 0.499535 | 0.038672 |
| A0A087Y8I7 | 1.502717 | 2.83E-09 |
| H2T855 | 0.533865 | 1.29E-07 |
| I3K850 | 0.415759 | 1.23E-08 |
| A0A147A8H0 | 1.671652 | 2.58E-07 |
| I3KHP9 | 0.501911 | 5.03E-06 |
| A0A023I4N4 | 2.686644 | 3.43E-08 |
| A0A1A8F8I9 | 1.507032 | 2.73E-07 |
| A0A1A7X824 | 1.580623 | 3.51E-07 |
| A0A087XCG6 | 0.285364 | 2.07E-05 |
| A0A146ZWI9 | 0.538475 | 9.36E-09 |
| A0A146N239 | 0.635484 | 1.24E-07 |
| H2LEW8 | 1.631124 | 1.87E-05 |
| A0A0S7LMA0 | 0.590158 | 5.36E-08 |
| A0A087XAG8 | 2.270402 | 1.57E-08 |
| I3KU28 | 0.184214 | 2.24E-07 |
| A0A0F8AR61 | 0.647696 | 4.51E-08 |
| Q4RRA7 | 0.584303 | 5.89E-07 |
| H2TKD8 | 0.45029 | 8.55E-08 |
| A4UYK4 | 0.57769 | 7.5E-08 |
| A0A146N1E5 | 2.193999 | 2.1E-08 |
| G3QCB5 | 1.544518 | 5.42E-05 |
| H3D5S1 | 0.481054 | 1.17E-07 |
| A0A0S7LE19 | 1.605842 | 5.07E-09 |
| A0A1A8JQT7 | 0.51434 | 4.62E-07 |
| A0A087XS77 | 1.771135 | 5.86E-05 |
| A0A087X9U0 | 0.614051 | 0.000101 |
| A0A0S7HWZ5 | 0.487602 | 4.73E-07 |
| B9V2Y7 | 0.184972 | 6.81E-07 |
| H2LCL1 | 2.11061 | 3.38E-09 |
| H2T463 | 1.586667 | 4.22E-08 |
| A0A0F8AIE0 | 1.738383 | 2.5E-11 |
| A0A0F8B3A1 | 0.658043 | 1.65E-05 |
| G3PVS6 | 2.649812 | 5.07E-07 |
| M4AZF6 | 1.689996 | 5.13E-08 |
| Q4T1Z1 | 1.806119 | 0.000978 |
| Q4RRW7 | 1.818146 | 6.81E-07 |
| H3D398 | 1.604024 | 1.08E-07 |
| H2MEF1 | 0.125457 | 2.31E-09 |
| A0A146TQU2 | 0.664121 | 4.79E-07 |
| G3N724 | 1.86145 | 7.24E-06 |
| G3NIZ5 | 1.5681 | 4.81E-05 |
| A0A146Y5H0 | 1.67483 | 1.2E-05 |
| A0A146NN45 | 1.815313 | 5.36E-05 |
| A0A1A7XLK6 | 0.57622 | 0.000532 |
| A0A146NP97 | 0.587642 | 3.15E-06 |
| A0A147AWY8 | 1.957143 | 1.56E-08 |
| H2TXA1 | 2.205597 | 2.79E-07 |
| A0A146ZXL3 | 2.176975 | 4.58E-08 |
| A0A0F8B2E3 | 1.52134 | 9.88E-07 |
| A0A0S7GY16 | 0.522059 | 1.63E-07 |
| M9QRZ6 | 2.313877 | 1.07E-07 |
| A0A146UX93 | 0.270602 | 1.4E-06 |
| H2UZZ9 | 1.845392 | 6.18E-09 |
| A0A1A8QR89 | 1.508165 | 4.2E-08 |
| A0A146XVJ6 | 1.533333 | 2.52E-08 |
| A0A146ZY27 | 0.211949 | 2.72E-07 |
| I3JGP6 | 3.382353 | 3.34E-08 |
| G3Q4H9 | 1.600432 | 3.65E-07 |
| Q4SKR4 | 1.707034 | 1.54E-08 |
| G3P7M3 | 1.815063 | 2.63E-05 |
| A0A146MPK8 | 1.807583 | 1.78E-06 |
| I3KFQ6 | 1.604429 | 4.13E-05 |
| G3NYU8 | 1.943515 | 1.81E-05 |
| C3KHF5 | 1.637028 | 0.000163 |
| G3Q9L0 | 1.558914 | 0.000661 |
| A0A146VXA7 | 0.508076 | 9.74E-06 |
| A0A147B2D5 | 0.387796 | 2.88E-05 |
| A0A146X2I2 | 0.649669 | 0.000122 |
| A0A0F8AK62 | 1.595596 | 1.42E-07 |
| I3IXM7 | 0.619779 | 1.79E-05 |
| A0A0F8ATD5 | 1.754422 | 0.000202 |
| I3JTG4 | 0.579982 | 4.04E-06 |
| F8QR94 | 0.558987 | 0.00201 |
| H2MRP5 | 0.468706 | 9.85E-10 |
| A0A0F8CHP8 | 1.53062 | 0.000155 |
| E6ZIR2 | 1.850325 | 4.46E-07 |
| A0A0F8B866 | 1.555288 | 5.3E-07 |
| M4AV02 | 1.534615 | 2.19E-05 |
| A0A146UZ57 | 0.560246 | 6.37E-05 |
| Q4RMZ0 | 0.098008 | 6.58E-08 |
| A0A0C5I1M5 | 1.71635 | 1.05E-05 |
| A0A0F8BSB8 | 1.614951 | 2.39E-06 |
| G3P2U9 | 2.460993 | 1.47E-07 |
| A0A146QYH4 | 1.572947 | 8.86E-06 |
| A0A146ZX14 | 1.791213 | 7.57E-07 |
| I3KGU8 | 0.579135 | 7.38E-08 |
| H2LCD6 | 1.669087 | 0.00092 |
| H2V355 | 1.724481 | 8.29E-05 |
| G3PIA0 | 0.4926 | 4.6E-05 |
| I3KXU5 | 1.605728 | 9.1E-07 |
| H2SCJ4 | 1.563452 | 1.04E-08 |
| A0A146MPH3 | 0.598315 | 4.63E-06 |
| A0A087X7Y5 | 0.446792 | 2.08E-08 |
| A0A0B4KJI1 | 1.6958 | 0.005953 |
| A0A096LU01 | 1.649279 | 2.56E-06 |
| A0A087X410 | 0.629551 | 1.07E-06 |
| A0A087XZU6 | 1.939208 | 1.31E-06 |
| A0A1A8F833 | 0.26915 | 4.6E-09 |
| A0A1A7YAX1 | 1.973515 | 3.53E-05 |
| A0A1A8AUF7 | 1.521634 | 4.52E-06 |
| A0A0F8CJN4 | 0.402309 | 4.41E-06 |
| A0A1A8UZC8 | 3.4755 | 1.22E-07 |
| A0A096M3S7 | 0.205014 | 2.85E-09 |
| A0A146NT87 | 0.555272 | 5.39E-08 |
| A0A0F8AVG0 | 4.213492 | 1.58E-09 |
| A0A1A7WGQ0 | 2.554832 | 9.48E-10 |
| A0A087XRT6 | 1.509487 | 6.88E-05 |
| A0A146Y9C3 | 2.075119 | 1.18E-05 |
| A0A147AQR1 | 1.605329 | 2.23E-07 |
| A0A1A8E5K4 | 1.67523 | 0.000508 |
| I3J4P9 | 0.21215 | 1.65E-06 |
| I3IU49 | 0.641799 | 5.05E-05 |
| A0A146NBU1 | 1.678051 | 1.49E-06 |
| H2SC39 | 2.340483 | 1.8E-09 |
| G3NN59 | 0.489643 | 5.64E-09 |
| Q4RNU4 | 0.593739 | 0.0001 |
| A6BMG5 | 0.160687 | 1.01E-11 |
| G3NQ45 | 0.639169 | 3.79E-05 |
| H2VC08 | 1.804764 | 5.54E-07 |
| D2KQG1 | 3.905066 | 6.45E-06 |
| I3J065 | 0.53884 | 3.55E-08 |
| A0A1A8ANY3 | 1.650747 | 0.000181 |
| I3JFK2 | 1.512796 | 0.002937 |
| C6GKU5 | 2.536127 | 0.000357 |
| A0A087XGK3 | 0.647811 | 2.02E-05 |
| I1SRJ0 | 0.518323 | 2.74E-08 |
| A0A146VCV7 | 1.822276 | 0.000498 |
| Q4RZ58 | 1.646729 | 2.13E-06 |
| A0A0F8AP91 | 1.699962 | 0.001303 |
| A0A146NWD3 | 1.684711 | 0.000191 |
| E6ZID3 | 1.540243 | 0.042385 |
| H3CUE1 | 0.459052 | 1.67E-05 |
| A0A0F8B3W8 | 0.544327 | 6.13E-08 |
| H2MQD3 | 0.340667 | 9.74E-09 |
| I3JXF4 | 0.323703 | 5.77E-06 |
| A0A0F8BCY5 | 2.145602 | 0.001571 |
| H2SCA6 | 1.874946 | 1.6E-06 |
| I3JNH6 | 1.550018 | 7.88E-05 |
| C3KJP1 | 0.631964 | 4.17E-06 |
| A0A147B0G5 | 1.563701 | 1.62E-05 |
| A0A096LQ73 | 1.878294 | 6.28E-05 |
| H3CVU7 | 0.401713 | 0.001599 |
| A0A1A8BEA5 | 2.063136 | 3.67E-08 |
| I3JQ89 | 0.484487 | 4.92E-07 |
| A0A0S7LPQ0 | 1.552696 | 3.19E-08 |
| H2L3I5 | 0.215623 | 6.49E-10 |
| A0A147ABM7 | 1.756366 | 1.45E-07 |
| A0A087XPH2 | 1.570437 | 0.000309 |
| A0A0M6A8S9 | 0.650784 | 5.11E-06 |
| G3NL30 | 0.423099 | 0.000207 |
| I3IUQ3 | 0.611859 | 3.18E-06 |
| A0A146V5P7 | 1.601203 | 7.24E-05 |
| H2ULX8 | 4.361098 | 2.27E-07 |
| E6ZII0 | 1.824061 | 0.000721 |
| D6PVQ7 | 0.211773 | 3.14E-07 |
| A0A0F8AEF4 | 1.608471 | 1.69E-05 |
| I3KUP1 | 0.60696 | 1.82E-07 |
| A0A087YNW8 | 0.187284 | 2.55E-07 |
| A0A087X9R3 | 0.628167 | 3.27E-07 |
| A0A146ZMN1 | 3.285597 | 2.28E-06 |
| A0A146X619 | 1.678434 | 0.00012 |
| H3BY31 | 1.628844 | 3.81E-05 |
| I3J105 | 1.611188 | 8.93E-07 |
| G3NML3 | 1.957348 | 1.31E-05 |
| A0A096MGR1 | 1.540109 | 3.14E-06 |
| A0A0F8CE61 | 1.64838 | 6.09E-07 |
| A0A1A8F3H5 | 0.572863 | 1.19E-07 |
| G3NRD6 | 0.584873 | 8.58E-07 |
| A0A146X988 | 1.579315 | 6.91E-07 |
| H2T4S6 | 0.389631 | 1.33E-07 |
| H2MXX7 | 1.989506 | 1.07E-05 |
| A0A146NQH4 | 1.930979 | 1.61E-07 |
| A0A0F8C342 | 1.678559 | 0.000633 |
| H3C539 | 2.580958 | 1.58E-05 |
| A0A0F8CTY6 | 2.195309 | 7.27E-08 |
| M4AJA7 | 0.497686 | 0.001092 |
| A0A146NKZ7 | 1.832266 | 0.000113 |
| H2S9H0 | 0.645858 | 0.000163 |
| A0A146ZXK5 | 2.237879 | 6.88E-07 |
| A0A096MDF5 | 2.863874 | 0.000999 |
| A0A0F8B6X1 | 0.613157 | 0.001899 |
| A0A146WEC7 | 1.555193 | 0.000665 |
| G3PDN6 | 1.967092 | 1.06E-06 |
| A0A0S7KUC6 | 2.1793 | 8.63E-07 |
| A0A087XXL1 | 3.376251 | 1.49E-07 |
| H2TXA4 | 1.843955 | 2.94E-07 |
| A0A147AU82 | 1.523256 | 0.000111 |
| Q4SXM5 | 0.60725 | 5.65E-07 |
| G3P095 | 2.332842 | 1.75E-06 |
| G3Q6B2 | 2.159732 | 8.8E-08 |
| H3CRT8 | 0.5967 | 2.42E-06 |
| A0A0F8C2I3 | 1.520216 | 4.65E-05 |
| A0A1A7Z4H5 | 1.893685 | 1.53E-06 |
| H2SC71 | 2.72716 | 6.05E-08 |
| A0A1A8SDD2 | 1.791147 | 2.06E-07 |
| E6ZHU2 | 1.550823 | 6.23E-06 |
| A0A0F8BQ77 | 2.737912 | 1.27E-05 |
| H3CP29 | 3.189767 | 2.74E-07 |
| A0A087XKD8 | 2.138488 | 1.7E-06 |
| H2SKB5 | 0.545894 | 2E-06 |
| M3ZYX5 | 0.249603 | 1.93E-10 |
| A0A146NDW6 | 0.350302 | 7.87E-07 |
| I3JTG8 | 3.080283 | 4.68E-06 |
| I3KRD2 | 0.626652 | 4.82E-06 |
| A0A0F8AJH2 | 0.236541 | 2.01E-07 |
| I3KGW2 | 3.881553 | 9.13E-09 |
| A0A147A1Z9 | 0.55771 | 1.33E-06 |
| A0A0F8BY36 | 1.541953 | 0.000188 |
| A0A146NU39 | 1.843498 | 7.23E-07 |
| H2T455 | 1.542977 | 3.58E-05 |
| A0A0F8C8F1 | 1.776006 | 5.9E-08 |
| A0A146NQ87 | 0.486107 | 3.45E-06 |
| A0A146WJJ2 | 1.581737 | 0.001441 |
| Q4RPX9 | 2.095316 | 2.26E-07 |
| A0A0F8CPI0 | 2.284466 | 4.11E-07 |
| A0A146X253 | 0.463959 | 2.79E-06 |
| H2V901 | 1.83522 | 6.56E-06 |
| A0A087XVX5 | 0.577409 | 1.36E-06 |
| A0A147ATV2 | 1.554079 | 7.02E-05 |
| A0A0F8ADC0 | 1.760845 | 0.000641 |
